# Introducing affinity and selectivity into galectin-targeting nanoparticles with fluorinated glycan ligands†

**DOI:** 10.1039/d0sc05360k

**Published:** 2020-11-16

**Authors:** Sarah-Jane Richards, Tessa Keenan, Jean-Baptiste Vendeville, David E. Wheatley, Harriet Chidwick, Darshita Budhadev, Claire E. Council, Claire S. Webster, Helene Ledru, Alexander N. Baker, Marc Walker, M. Carmen Galan, Bruno Linclau, Martin A. Fascione, Matthew I. Gibson

**Affiliations:** Department of Chemistry, University of Warwick CV4 7AL UK m.i.gibson@warwick.ac.uk; Warwick Medical School, University of Warwick CV4 7AL UK; Department of Physics, University of Warwick CV4 7AL UK; Department of Chemistry, University of York Heslington York YO10 5DD UK martin.fascione@york.ac.uk; School of Chemistry, University of Southampton Highfield Southampton SO171BJ UK bruno.linclau@soton.ac.uk; School of Chemistry, University of Bristol Cantock's Close Bristol BS8 1TS UK

## Abstract

Galectins are potential biomarkers and therapeutic targets. However, galectins display broad affinity towards β-galactosides meaning glycan-based (nano)biosensors lack the required selectivity and affinity. Using a polymer-stabilized nanoparticle biosensing platform, we herein demonstrate that the specificity of immobilised lacto-*N*-biose towards galectins can be ‘turned on/off’ by using site-specific glycan fluorination and in some cases reversal of specificity can be achieved. The panel of fluoro-glycans were obtained by a chemoenzymatic approach, exploiting BiGalK and BiGalHexNAcP enzymes from *Bifidobacterium infantis* which are shown to tolerate fluorinated glycans, introducing structural diversity which would be very laborious by chemical methods alone. These results demonstrate that integrating non-natural, fluorinated glycans into nanomaterials can encode unprecedented selectivity with potential applications in biosensing.

## Abstract 2

A chemo-enzymatic site-specific fluorination strategy is employed to obtain glyco-nanoparticles with tuneable selectivity towards galectins.

## Graphical abstract

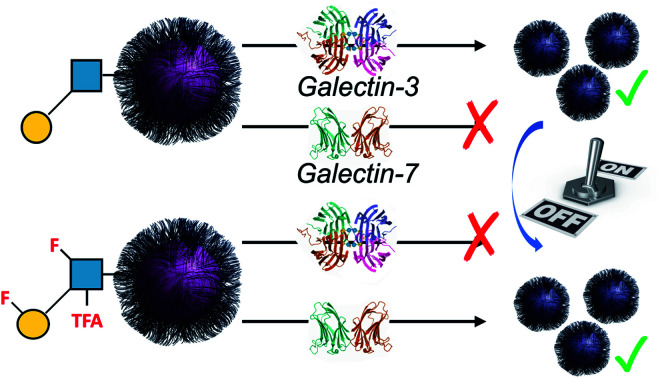

## Introduction

Galectins are a large group of soluble β-galactoside binding proteins which are targets for therapy and diagnostics, compared to other human lectin families which are typically membrane-bound.^1–3^ Galectin-3 for example is overexpressed in prostate cancers^4^ leading to endothelial cell adhesion,^5^ nanomolar glycopeptide inhibitors of Galectin-3 have been shown to suppress metastasis^6^ and several galectin-binders have advanced to clinical trials.^7^ However, as all galectins bind terminal β-galactosides to some extent, it is a significant challenge to selectively target individual galectins.^2^ Percec and co-workers have employed dendrimeric scaffolds to probe how multivalent presentation of glycans affects galectin binding showing how topology and ligand density can be used to tune affinity.^8,9^ Despite the promise of using glycans to detect analytes, antibody reagents remain the main clinical tools used in ELISA,^10^ lateral flow^11^ or flow cytometry assays.

The installation of glycans onto polymer-coated gold nanoparticles is a powerful technology to probe lectin binding.^12,13^ The polymer coating provides steric stabilization to prevent aggregation in complex media, and the incorporation of multiple copies of a glycan at the polymer chain ends, increases affinity due to the cluster glycoside effect.^14^ Gold nanoparticles have unique optical properties,^15,16^ which enables signal generation through aggregation^13,17–19^ in lateral flow devices,^20,21^ and also in surface enhanced Raman spectroscopy.^22^ However, most studies with multivalent glycans involve mono/di-saccharides which have shown limited selectivity so far.^23^ There is therefore a knowledge and technological gap, to develop synthetically-accessible multivalent probes, which are also endowed with selectivity.^24^

Fluorination of glycans influences their physicochemical properties and hence modulates their biological function.^25–28^ While fluorine substitution has little effect on glycan conformation,^29,30^ it can influence hydrogen bonding properties of adjacent hydroxyl groups,^31,32^ and fluorine itself is a weak hydrogen bond acceptor but not a hydrogen bond donor.^33,34^ Furthermore, fluorine atoms can form attractive multipolar interactions with proteins,^35,36^ and these have been observed with fluorinated carbohydrate derivatives,^37^ including galectin binders.^38^ Fluorinated sialyl oligosaccharides displayed significantly higher binding affinities for the *Toxoplasma gondii* lectin, TgMIC1 in comparison to their non-fluorinated counterparts.^39^ Similarly, fluorinated MUC-1 antigens displayed enhanced immunogenicity and differential binding affinity to mouse antisera, making them useful tools for probing humoral immune responses.^40^ Fluorinated glycans have also proven effective for probing carbohydrate–lectin structure–activity relationships. For example, Glcα1–3ManαMe analogues fluorinated around the Glc moiety revealed that the 2- and 3-OH group of Glc were important for calreticulin binding, but not the 6-OH.^41^ Similarly, the 6-OH group of the α-1,6-branched mannose in the Man3GlcNAc2 glycan, was shown to be important for Concanavalin A binding.^42^

A powerful route to diversify unnatural glycans is to incorporate an enzymatic step. By using promiscuous enzymes for glycosidic bond formation,^27^ which are capable of accepting chemically accessible fluorinated glycans, building blocks can be combined, producing anomerically pure compounds, facilitating purification.

Herein we report a chemoenzymatic route to selectively fluorinated lacto-*N*-biose (Gal-β1–3-GlcNAc) glycans, including fluorination at both sugar residues, and their integration into a multivalent glyconanoparticle platform. We demonstrate that site-selective fluorination enables modulation of the affinity and introduces high selectivity towards Galectins 3 and 7 which is not possible using native glycans. This approach demonstrates the potential for the translation of glyconanomaterials to applications in therapy and biosensing.

## Results and discussion

**Fig. 1:**
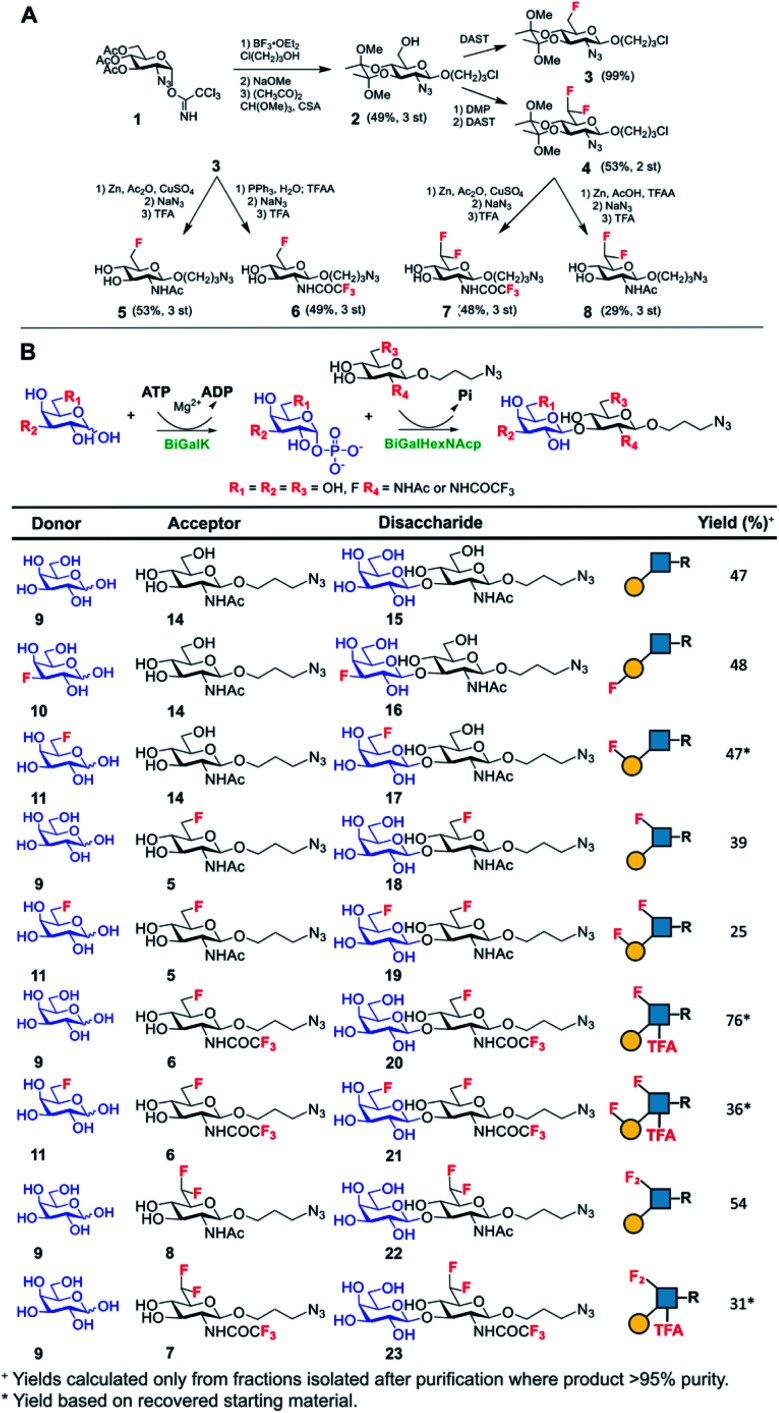
(A) Chemical syntheses of fluorinated acceptors. (B) Fluorinated lacto-*N*-biose analogues prepared using a chemoenzymatic strategy with BiGalK and BiGalHexNAcP. TFA = trifluoroacetyl.

Lacto-*N*-biose has confirmed affinity towards Galectin-3,^43^ so a library of nine fluorinated lacto-*N*-biose derivatives was synthesised, using a modular chemoenzymatic approach (Fig. 1 and ESI†). Glycans were designed with an azido-propyl tether for subsequent nanoparticle immobilization.^17,44^ This strategy introduces diversity through the chemical fluorination of the individual monosaccharide building blocks, galactose (Gal) and *N*-acetylglucosamine (GlcNAc), prior to enzymatic glycosylation using a one-pot, two enzyme strategy. In this system, the kinase BiGalK^45^ catalyses the formation of galactose-1-phosphates (Gal-1Ps), before the phosphorylase BiGalHexNAcP^46^-catalyzed transfer of Gal-1Ps to GlcNAc acceptors, by reverse phosphorylysis.^47^ As several fluorinated Gal derivatives are commercially available, we focused on chemical diversification of the GlcNAc acceptor. BiGalHexNAcP was previously shown to be highly tolerant to modifications at the 2- and 6-positions of GlcNAc and GalNAc,^46^ so we focused our efforts on introducing fluorine to these positions (Fig. 1A). During a preliminary screen for BiGalHexNAcP donor specificity, we found Gal (9), 3FGal (10) and 6FGal (11) to be suitable donors, while little or no activity was displayed towards 2FGal (12) and 4FGal (13) in the one-pot, two enzyme system, when using GlcNAc-N_3_ (14) as the acceptor (data not shown). Lacto-*N*-biose and fluorinated derivatives were efficiently synthesized on semi-preparative scale using donors 9–11 and acceptors 5–8 & 14 (Fig. 1B and ESI†). Excess amounts of donor sugar (2–10 equiv.) were used to drive the reactions towards disaccharide formation. For the less preferred substrates (*e.g.* 3FGal), extended reaction times (up to 144 h) and the sequential addition of enzyme were used to achieve maximum conversion. As high purity was required, all glycans were subjected to a two-step purification (gel filtration and flash or anion-exchange chromatography). In total, eight fluorinated disaccharides (16–23) bearing azidopropyl linkers were prepared, in addition to lacto-*N*-biose derivative (15), in isolated yields ranging from 25–76%.

**Fig. 2:**
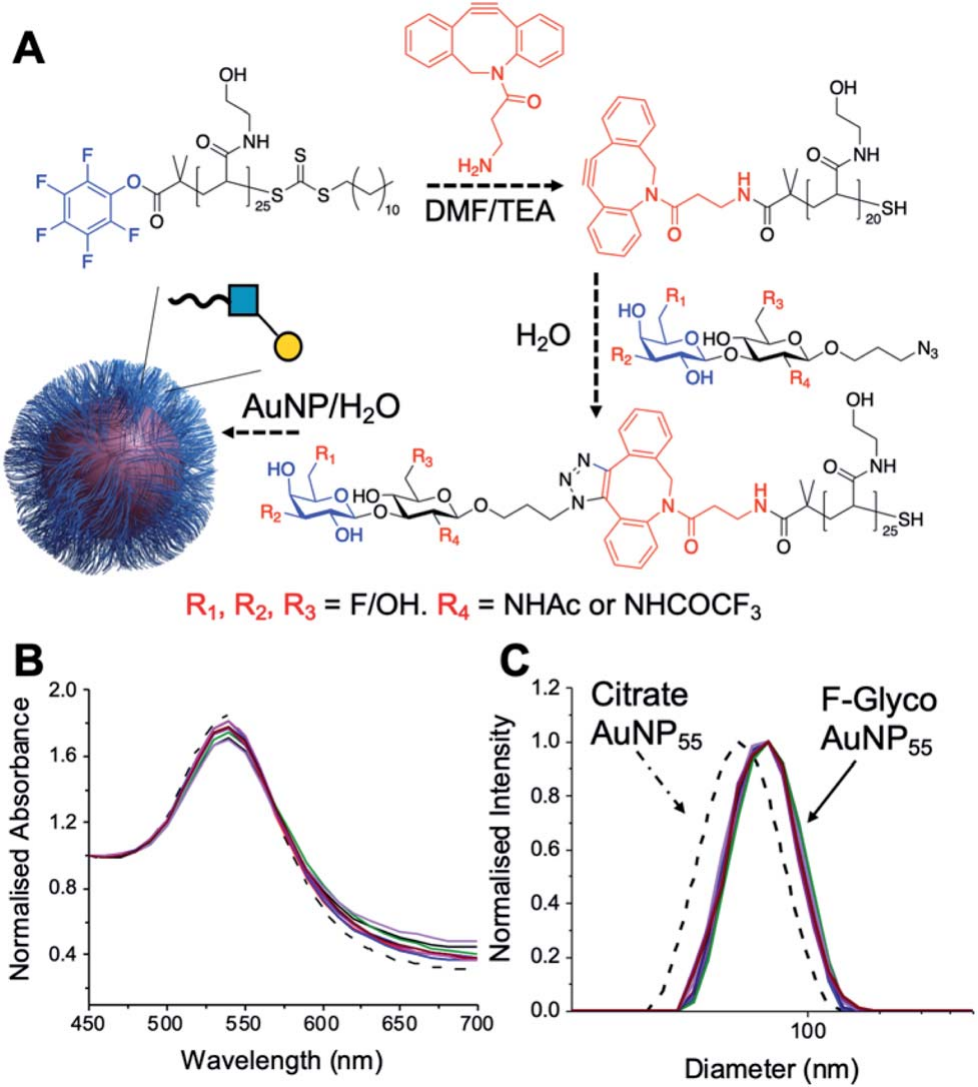
Nanoparticle synthesis and characterization. (A) Synthetic route to conjugate fluoro-glycans onto nanoparticles; (B) UV-Vis traces of all nanoparticles showing colloidal stability; (C) dynamic light scattering (DLS) of all nanoparticles showing size increase upon polymer coating.

PHEA (poly(hydroxylethyl acrylamide)) coated gold nanoparticles were selected for the screening, as these are an established platform for glycan binding analysis.^5,39^ This tool requires small (μg) quantities of glycans and hence is ideal for screening compared to calorimetry or NMR-based approaches which need more material, which is not always available. RAFT (reversible addition–fragmentation chain transfer) polymerization was used to obtain telechelic PHEA ligands bearing a pentafluorophenyl (PFP) group at the α-terminus (Fig. 2A).^44,48^ The PFP was displaced by dibenzocyclooctyne-amine, introducing a handle (validated by ^19^F NMR) to capture the glycosyl azide, by strain promoted azide/alkyne click (SPAAC). By using RAFT, an ω-terminal thiol was also produced enabling assembly of the glycoligands onto 55 nm gold nanoparticles with excess polymer removed by centrifugation/resuspension cycles. The nanoparticle size and polymer chain length (DP25) used were guided by previous work, to give a balance between colloidal stability and aggregation responses.^39^ UV-visible spectroscopy showed the characteristic SPR band (533 nm) and no aggregation (at 700 nm) after polymer coating (Fig. 2B). Dynamic light scattering showed a small increase in hydrodynamic diameter consistent with polymer coating (Fig. 2C). X-ray photoelectron spectroscopy (XPS, in ESI†) confirmed the presence of the polymers and the fluorine from the glycans.

**Fig. 3:**
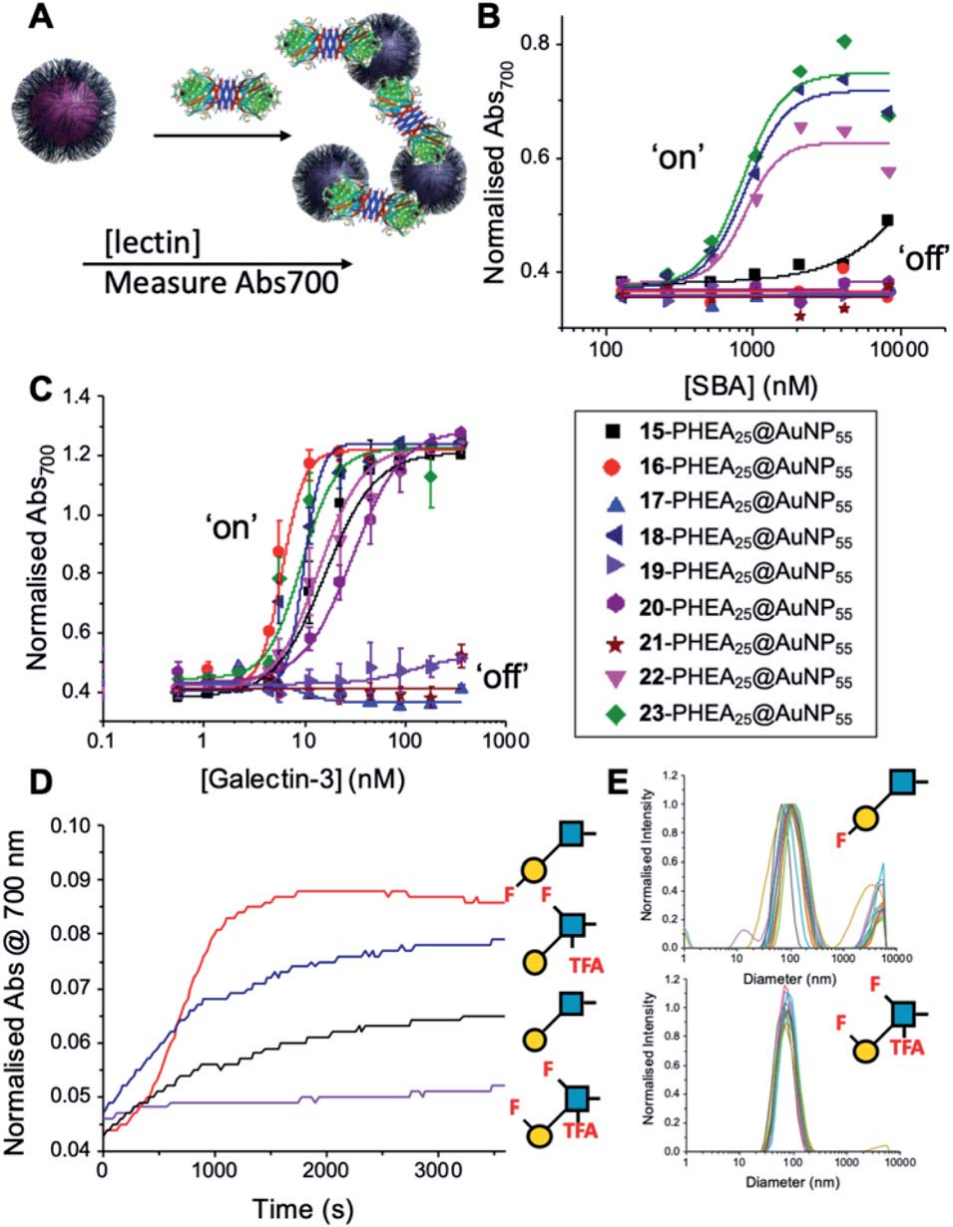
Screening of lectin/F-GlycoNP binding. (A) Schematic of aggregation assay; (B) dose–response to Soybean agglutinin (SBA); (C) dose–response to Galectin-3; (D) aggregation kinetics with Galectin-3; (E) dynamic light scattering with Galectin-3.

With this panel of fluoro-glycan nanoparticles (GlycoAuNPs) in hand, their lectin binding affinity/selectively trends could be evaluated, initially using soybean agglutinin (SBA) which preferentially binds β-d-galactosides.^13,49^ Binding was assessed by exploiting the optical properties of the GlycoAuNPs, whereby SBA binding leads to aggregation of the nanoparticles (Fig. 3A). This results in a red-blue colour shift which can be assessed by UV-visible spectroscopy (Fig. 3B).^17,18,50^ As expected, lacto-*N*-biose (15) showed weak affinity towards SBA (*K*_D_, apparent > 10 μM; *K*_D_ values for multivalent systems are very challenging to determine). Fluorine addition to the GlcNAc unit improved the binding >12-fold, where Gal-β(1,3)-6FGlcNTFA (20, dark blue line), Gal-β(1,3)-6,6diFGlcNAc (22, pink line) and Gal-β(1,3)-6,6diFGlcNTFA (23, green line) all show *K*_D_,apparent values in the range of 0.84–0.89 μM. Furthermore Gal-β(1,3)-6FGlcNAc (18, dark purple line) does not have sufficient fluorine incorporation to see this increase in binding. Fluorination in any position around the galactose ring was not tolerated, resulting in decreased binding affinity in the cases of 6FGal-β(1,3)-6FGlcNTFA (21) compared to Gal-β(1,3)-6FGlcNTFA (20).

Guided by these experiments with SBA, Galectin-3 binding was profiled (Fig. 3C). Galectin-3 has only a single binding site, but is in equilibrium with a pentameric form, and hence can cross-link multivalent glycomaterials.^51^ Lacto-*N*-biose (15) particles bound Galectin-3, agreeing with previous observations from Hsieh *et al.*^43^ However, a number of fluorinated lacto-*N*-biose derivatives bound with a greater affinity to Galectin-3 than native (15), with 3FGal-β(1,3)-GlcNAc (16), Gal-β(1,3)-6,6diFGlcNTFA (23), Gal-β(1,3)-6FGlcNTFA (20) and Gal-β1(1,3)-6,6diFGlcNAc (22) all showing enhanced binding. In contrast, any glycan with a 6FGal derivative, such as 6FGal-β(1,3)-GlcNAc (17), 6FGal-β(1,3)-6FGlcNAc (19) and 6FGal-β(1,3)-6FGlcNTFA (21) completely ‘switched off’ the binding to Galectin-3. Kinetic analysis of aggregation agreed with dose–response (Fig. 3D) data, with 3FGal-β(1,3)-GlcNAc (16) showing the fastest rate. This was confirmed by dynamic light scattering (Fig. 3E) showing that ‘non-binder’ 6FGal-β(1,3)-6FGlcNTFA (21) does not lead to aggregation. This provides strong evidence that subtle site-specific fluorination is a powerful tool to introduce affinity and selectivity into glycans against biomedically relevant lectins, when conjugated to nanoparticles.

**Fig. 4:**
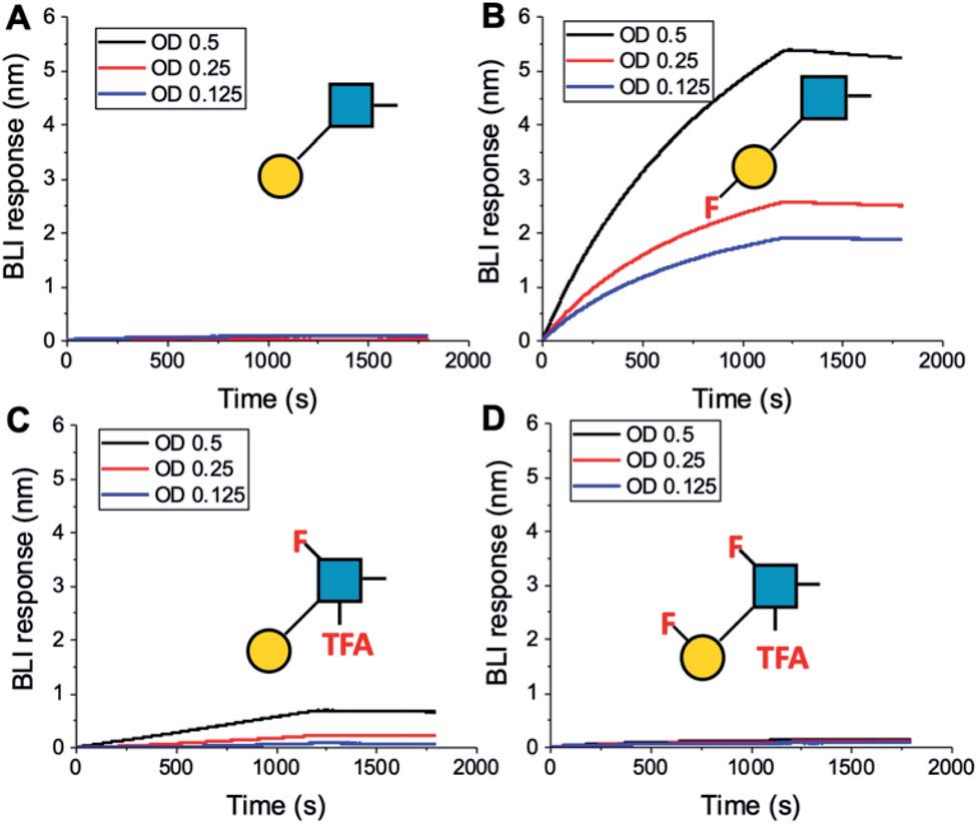
Biolayer interferometry analysis of binding of AuNPs to Galectin-3. (A) Lacto-*N*-biose (15); (B) 3FGal-β(1,3)-GlcNAc (16); (C) Gal-β(1,3)-6FGlcNTFA (20); (D) 6FGal-β(1,3)-6FGlcNTFA (21).

To further validate the aggregation-based assays, biolayer interferometry (BLI) was employed.^12^ Galectin-3 was biotinylated, then immobilized onto streptavidin-functional BLI sensors, and the GlycoAuNPs applied (Fig. 4). Lacto-*N*-biose (15, Fig. 4A) showed little binding due to the concentrations used (to enable enhancements to be observed without saturation). In agreement with the aggregation-based assays, significant binding was observed using 3FGal-β(1,3)-GlcNAc (16, Fig. 4B), and there was some limited binding observed with Gal-β(1,3)-6FGlcNTFA (20, Fig. 4C). Also in line with the aggregation data, no binding was seen for 6FGalβ(1,3)-6FGlcNTFA (21, Fig. 4D). Indeed, consideration of the crystal structure (PDB entry 4XBN^43^) of Galectin-3 with lacto-*N*-biose reveals an interaction of the 6-OH of galactose with residues Asn174A/Glu184A, supporting our observation that 6-OH replacement with fluorine is detrimental for binding. The 3-OH group is not involved in H-bonding interactions and hence fluorination does not diminish binding, and instead appears to increase the overall affinity. Overall, these data conclusively show that site-specific fluorination enables precise modulation of binding affinity and could be used to generate nanoparticle biosensors for rapid detection of this important biomarker.

**Fig. 5:**
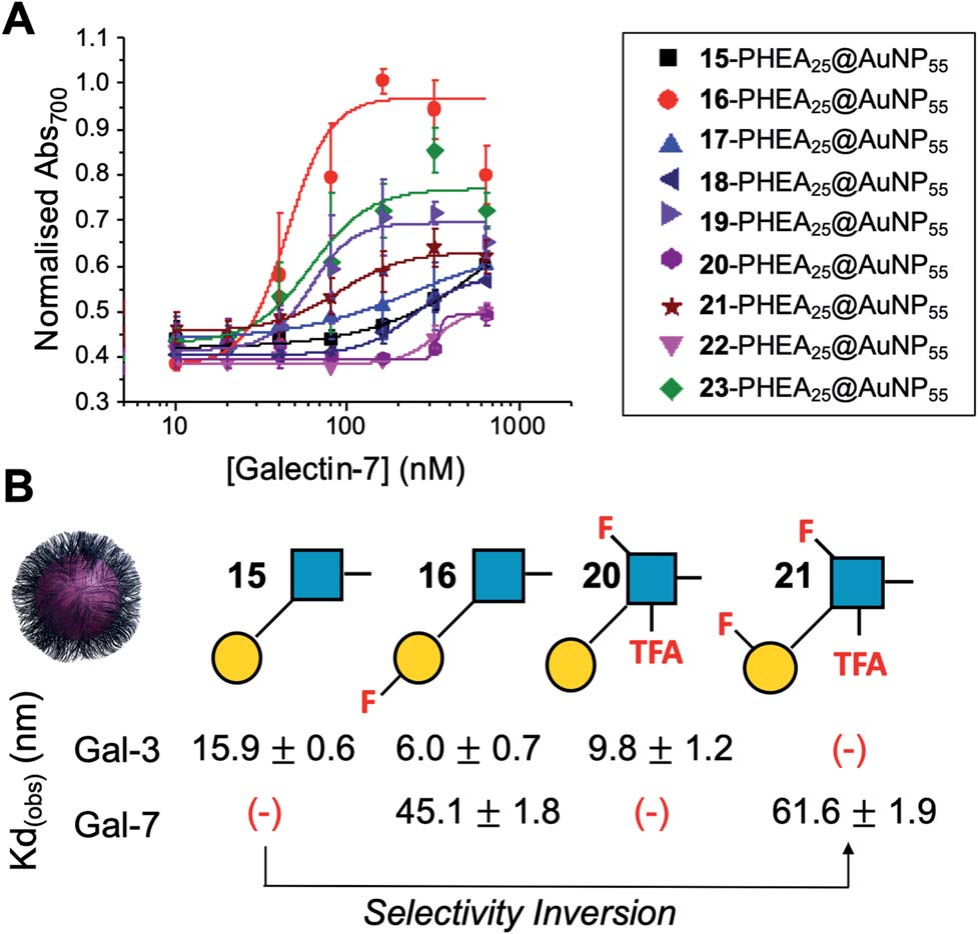
Galectin-7 binding to the F-glyconanoparticle library. (A) Dose–response curve for the AuNP aggregation assay; (B) summary of apparent *K*_D_ (nM) for selected glycans showing the fine-tuning and selectivity inversion. (−) = no binding.

Encouraged by the Galectin-3 binding data, the utility of these unique fluoro-glycan nanoparticles to discriminate between individual galectins was explored, which is often not possible with natural glycans nor with monosaccharide-based glycomaterials. Galectin-7 was chosen as it has previously been reported to have lower affinity towards lacto-*N*-biose (270 μM) than Galectin-3 (93 μM)^43^ and hence offers a robust challenge to explore how fluorination can be used to tune specificity/affinity. Binding of Galectin-7 to the library of particles by the aggregation assay (as described above) was conducted, and Fig. 5 shows the relative affinities as *K*_D_,apparent. Lacto-*N*-biose particles showed preference for Galectin 3 as anticipated, displaying limited binding to Galectin-7 in the concentration range tested. Introduction of fluorine atoms resulted in a variation of the observed *K*_D_'s, but in particular 6FGalβ(1,3)-6FGlcNTFA (21) showed switching of affinity compared to non-fluorinated ligands: this derivative showed no affinity to Galectin-3, but the fluorination results in ‘switching on’ of affinity towards Galectin-7. The extent of aggregation at plateau for 21 was lower than for 15, but clear binding was seen. It is important to highlight that these assays cannot identify if glycans engage the protein in the same manner, or at different (non-canonical) binding sites. This affinity switch shows that the site-specific incorporation of fluorine atoms can overcome the low selectivity of glycans towards their lectin partners and in some cases completely turn off interactions. Additional glycan modifications to a core lactosyl unit in a glycan array have also been reported to modulate galectin binding patterns, which is complementary to the approach taken here.^52^ Such selectivity is essential in the development of glyconano tools for therapy and diagnostics. Furthermore, this chemoenzymatic synthetic approach to glycan libraries may facilitate screening of binding epitopes by methods such as (STD) NMR^53–55^ which require more material and have lower throughput.

## Experimental

Full experimental details are in the electronic ESI.† This includes characterization of all glycans and nanomaterials.

## Conclusions

To conclude, a chemoenzymatic glycosylation strategy was employed for the rapid assembly of a diverse library of (multi)fluorinated lacto-*N*-biose derivatives, which were integrated into nanobiosensors. The efficient one-pot enzymatic glycosylation process confines the protecting group requirements to the chemical synthesis of the fluorinated acceptors, and reveals a large substrate tolerance of the BiGalK and BiGalHexNAcP enzymes. These fluoro-glycans were conjugated to polymer-stabilized gold nanoparticles, which were used to reveal unique binding patterns and significant enhancements in selectivity towards two Galectins. Due to the use of nanoparticles, only very low amounts (μg) of glycan per assay are required in contrast to other methods. It was discovered that a single fluorine at 3-position of the galactose residue dramatically enhanced binding towards Galectin-3. Fluorine at other locations dramatically reduced binding, with 6-fluorination abrogating all binding affinity. Galectin-7 was also screened which does not normally show any significant binding to the native lacto-*N*-biose. It was shown that selective fluorination allowed complete reversal of selectivity such that a pentafluorinated derivative only bound Galectin-7 and all binding to Galectin-3 was removed, which is an unprecedented switch in selectivity. This is notable as glycans normally display a range of binding affinities but here fluorination enables the introduction of binary on/off responses which may be useful in the design of biosensors, and innovative diagnostics. These findings show that subtle fluorination strategies can engineer marked selectivity into immobilized glycans. This will aid the development of new sensing platforms which are not accessible using native mono/disaccharides due to their broad binding affinities, and the development of glycan-diagnostics as alternatives to traditional antibody-based techniques.

## Conflicts of interest

There are no conflicts to declare.

## Supplementary Material

SC-012-D0SC05360K-s001
