# Supplementary material for: Introducing affinity and selectivity into galectin-targeting nanoparticles with fluorinated glycan ligands
Source: Chem Sci. 2020 Nov 16;12(3):905–10. doi: 10.1039/d0sc05360k (PMC8179109; doi:10.1039/d0sc05360k)
Supplement: SC-012-D0SC05360K-s001 [file SC-012-D0SC05360K-s001.pdf]

Electronic Supplementary Material (ESI) for Chemical Science.
This journal is © The Royal Society of Chemistry 2020

Supporting information for

Introducing affinity and selectivity into galectin-targeting nanoparticles with fluorinated glycan ligands.

Sarah-Jane Richards,[a‡] Tessa Keenan,[d‡] Jean-Baptiste Vendeveille,[e] David E. Wheatley,[e] Harriet Chidwick,[d] Darshita Budhadev,[d] Claire E. Council,[e] Clare S. Webster,[f] Helene Ledru,[f] Alexander N. Baker,[a] Marc Walker,[c] M. Carmen Galan,[f] Bruno Linclau,* Martin A. Fascione,[d] * Matthew I. Gibson,[a,b] *

Author affiliations:
a Department of Chemistry, University of Warwick, CV4 7AL, UK
b Warwick Medical School, University of Warwick, CV4 7AL, UK,
c Department of Physics, University of Warwick, CV4 7AL, UK
d Department of Chemistry, University of York, Heslington, YO10 5DD, York, UK
e School of Chemistry, University of Southampton, Highfield, Southampton SO171BJ, UK
f School of Chemistry, University of Bristol, Cantock's Close, Bristol, BS8 1TS, UK
‡ These authors contributed equally.

Emails m.i.gibson@warwick.ac.uk, martin.fascione@york.ac.uk and bruno.linclau@soton.ac.uk

Materials and general procedures.

All chemicals were purchased from Sigma-Aldrich, Fisher Scientific, Fluorochem or VWR and used without further purification, unless otherwise stated. 3-Deoxy-3-fluoro-galactose (**10**) and 6-deoxy-6-fluoro-galactose (**11**) were kindly provided by Carbosynth. BiGalHexNAcP and BiGalK were produced in house exactly as previously described,[1,2] or kindly provided by Prozomix Limited. Anhydrous solvents were purchased from commercial sources or obtained by distillation using standard procedures, or by passage through a column of anhydrous alumina using equipment from Anhydrous Engineering (University of Bristol) based on the Grubbs' design.[3] Reactions requiring anhydrous conditions were performed under N_2 or Ar; glassware and needles were either flame dried immediately prior to use, or placed in an oven (150 °C) for at least 2 h and allowed to cool in a desiccator or under reduced pressure. Liquid reagents, solutions or solvents were added via syringe through rubber septa; solid reagents were added via Schlenk type adapters. Reactions were monitored by TLC on Kieselgel 60F_{254} (Merck), with UV light (254 nm) detection and by staining with basic solution of $KMnO_4$, 10% H_2SO_4/EtOH + 0.4**%** *N*-(1-naphthyl)ethylenediamine dihydrochloride or p-anisaldehyde stain, followed by brief heating. Column chromatography was performed on silica gel (MERCK Geduran 60 Å, particle size 40–63 µm). All reported solvent mixtures are volume measures. Extracts were concentrated in vacuo using both a Heidolph HeiVAP Advantage rotary evaporator (bath temperatures up to 50 °C) at a pressure of 15 mmHg (diaphragm pump) or 0.1 mmHg (oil pump), as appropriate, and a high vacuum line at room temperature. Water soluble compounds were freeze dried on a Lytotrap Plus (LTE Scientific LTD). *N*-Hydroxyethyl acrylamide (97 %), 4,4'-Azobis(4-cyanovaleric acid) (98 %), mesitylene (reagent grade), triethylamine (> 99%), sodium citrate tribasic dihydrate (> 99 %), Gold(III) chloride trihydrate (99.9%), ammonium carbonate (reagent grade) and dibenzocyclooctyne-amine were all purchased from Sigma-Aldrich. 2-(Dodecylthiocarbonothioylthio)-2-methylpropionic acid pentafluorophenyl ester was synthesized as previously outlined by Richards *et al.*[8] Soybean Agglutinin and was purchased from Vector Laboratories. Galectin-3 was purchased from Abcam. Clear half area and black 96-well plates were purchased from Greiner Bio-one. Streptavidin (SA) biosensors were purchased from Forte Bio. Lectins and hemagglutinins were biotinylated using EZ-Link Sulfo-NHS-LC-Biotin reagent from Thermo Fisher Scientific using standard procedure (20-fold molar excess of biotin reagent, conjugation performed in PBS buffer and isolated using Amicon Ultra-0.5 mL 3000 MWCO centrifugal filters from Merck Millipore).

Physical and analytical procedures

NMR, IR and optical rotations

^{1}H, ^{13}C and ^{19}F NMR spectra were measured using either a Bruker 500-MR spectrometer at the University of York Centre for Magnetic Resonance, Bruker Ultrashield 400 or 500 MHz spectrometers at the University of Southampton, or with Varian or Bruker spectrometers operating at field strengths listed at the University of Bristol. The chemical shift (δ) is given in ppm using the residual solvent peak as an internal standard. Resonances were assigned where possible, using COSY, HSQC and HMBC experiments. TopSpin 3.5pl7, ACD/Labs (2018.1.1, ver S80S41) and MestReNova (v 11.0)

were primarily used for processing spectral data. IR spectra were recorded in the range 4000-400 cm^{-1} on Thermo Scientific Nicolet iS5 or Perkin Elmer Spectrum spectrometers as films or solids, and absorption peaks are given in cm^{-1}. Optical rotations were collected on an Optical Activity PolAAr 2001 machine.

Mass spectrometry

HRMS spectra were obtained by the University of York Centre of Excellence in Mass Spectrometry (CoEMS) and analysed on a Bruker Daltonics microTOF spectrometer, the University of Bristol mass spectrometry service by electrospray ionisation (ESI) or matrix assisted laser desorption ionisation (MALDI) modes or at the University of Southampton on a Bruker Daltonics MaXis time-of-flight (TOF) mass spectrometer. Low resolution electrospray mass spectra were recorded with a Waters Acquity TDQ mass tandem quadrupole mass spectrometer.

X-ray Photoelectron spectroscopy

The x-ray photoelectron spectroscopy (XPS) data was collected at the Warwick Photoemission Facility, University of Warwick. The samples were attached to electrically-conductive carbon tape, mounted on to a sample bar and loaded in to a Kratos Axis Ultra DLD spectrometer which possesses a base pressure below 1 x 10^{-10} mbar. Measurements were performed in the main analysis chamber, with the sample being illuminated using a monochromated Al Kα x-ray source. The measurements were conducted at room temperature and at a take-off angle of 90° with respect to the surface parallel. The core level spectra were recorded using a pass energy of 20 eV (resolution approx. 0.4 eV), from an analysis area of 300 μm x 700 μm. The spectrometer work function and binding energy scale of the spectrometer were calibrated using the Fermi edge and $3d_{5/2}$ peak recorded from a polycrystalline Ag sample prior to the commencement of the experiments. In order to prevent surface charging the surface was flooded with a beam of low energy electrons throughout the experiment and this necessitated recalibration of the binding energy scale. To achieve this, the C-C/C-H component of the C 1*s* spectrum was referenced to 285.0 eV. The data was analysed in the CasaXPS package, using Shirley backgrounds and mixed Gaussian-Lorentzian (Voigt) lineshapes. For compositional analysis, the analyser transmission function has been determined using clean metallic foils to determine the detection efficiency across the full binding energy range.

Chemical synthesis

Synthesis of fluorinated acceptors

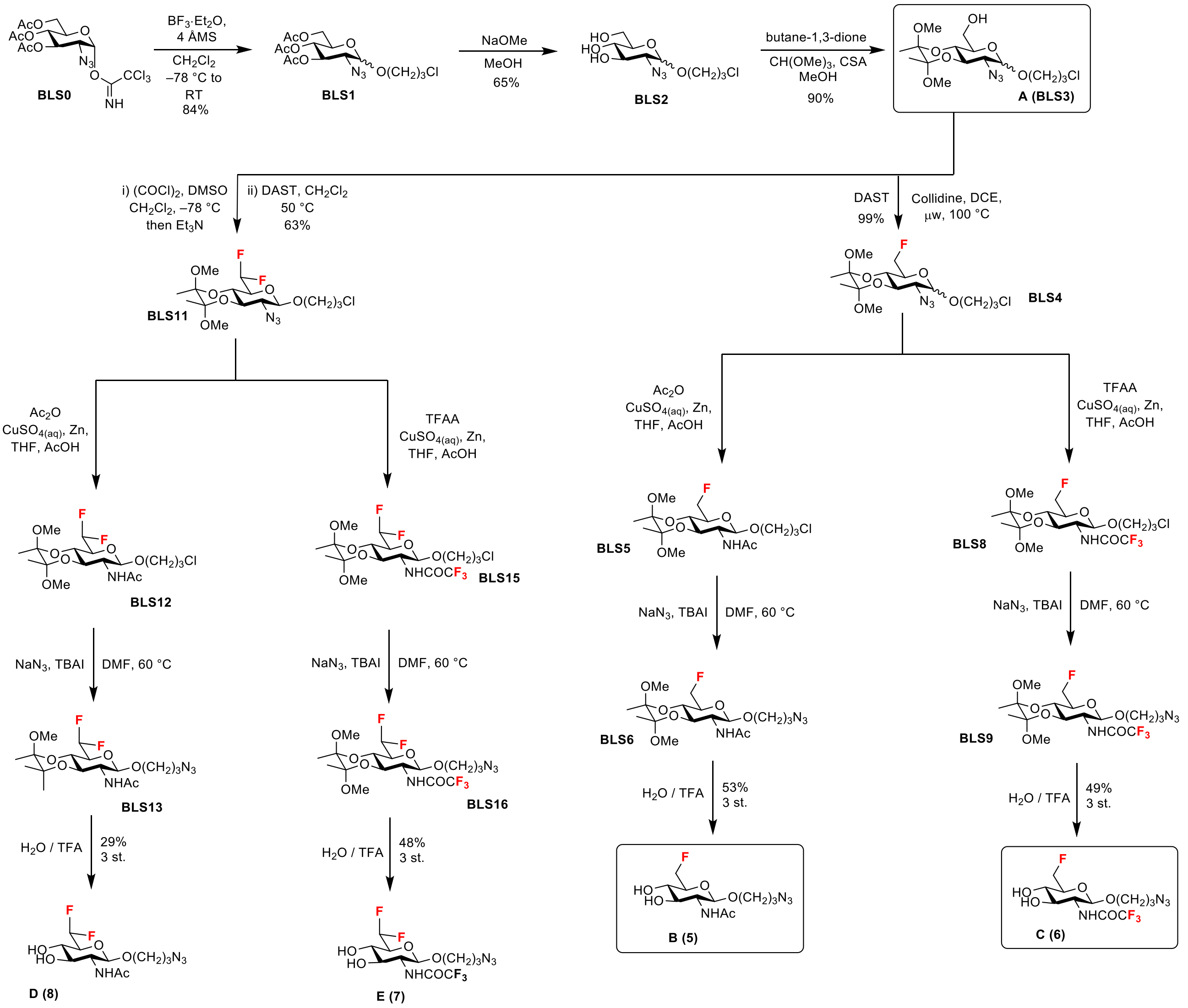

General procedures

General procedure A for the reduction of azide to NHAc / NHTFA

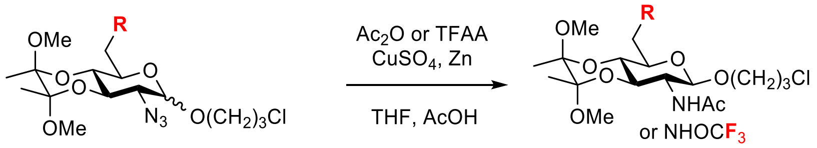

To a solution of azido sugar (1 equiv.) in THF (0.08 M) was added Zn dust (12 equiv.) and AcOH (23 equiv.). The solution was cooled to 0 °C, and 10% $CuSO_{4(aq)}$ (0.1 equiv.) was then added dropwise. The solution was allowed to warm to RT and stirred until completion (as indicated by TLC analysis). The mixture was filtered through Celite® and the filter cake washed with 10% MeOH/CH_2Cl_2. The filtrate was concentrated *in vacuo* to afford the crude amine, which was re-dissolved in THF (0.04 M)

and cooled to 0 °C. The desired anhydride (10 equiv.) was added, and the reaction warmed to RT and stirred for 16 h. The reaction was quenched with Et_3N and diluted with EtOAc. The solution was washed with sat. $NaHCO_{3(aq)}$, brine, dried ($MgSO_4$) and concentrated *in vacuo*. Purification by chromatography (silica, 20% EtOAc/hexane) afforded the amido sugars.

General procedure B for the conversion of the chloropropyl to the azidopropyl group

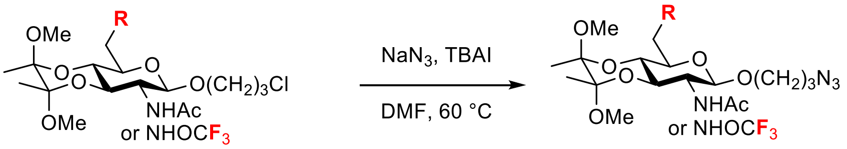

To a solution of alkyl chloride (2.20 g, 4.70 mmol) in DMF (0.2 M) was added NaN_3 (5 equiv.) and TBAI (0.1 equiv.). The solution was heated to 60 °C and stirred for 24 h, then cooled to RT. The solution was diluted with EtOAc and brine. The aqueous phase was separated and extracted with EtOAc. The combined organic layers were dried ($MgSO_4$) and concentrated *in vacuo*. Purification by chromatography (silica, 25% acetone/hexane) afforded the alkyl azides.

General procedure C for the deprotection of the BDA group

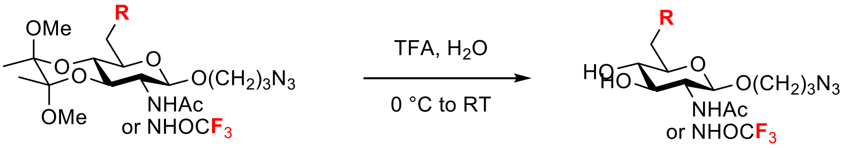

A suspension of the BDA-protected sugar (1 equiv.) in H_2O/TFA (1:7, 0.3 M) at 0 °C was warmed to RT and stirred for 45 min before being concentrated *in vacuo*. Purification by chromatography (silica, 60% acetone/CH_2Cl_2 for GlcNAc or 40% acetone/hexane for GlcNTFA) afforded the deprotected sugar.

Synthesis of A

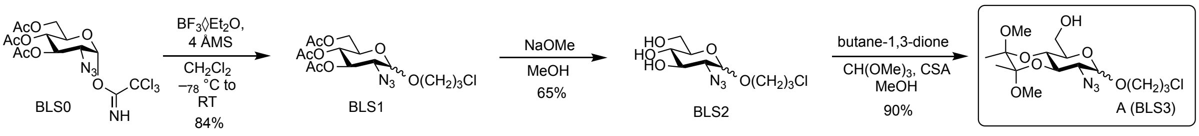

Synthesis of 3-chloropropyl 3,4,6-tri-*O*-acetyl-2-deoxy-2-azido-D-glucopyranoside (BLS1)

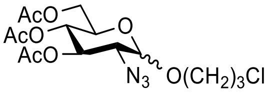

To a solution of **BLS0** [4] (22.9 g, 48.2 mmol) in CH_2Cl_2 (250 mL) at −78 °C was added 4 Å M.S. (30 g) and 1-chloropropan-3-ol (12.1 mL, 144 mmol). The solution was stirred at this temperature for 30 min, then $BF_3{\cdot}OEt_2$ (6.11 mL, 48.2 mmol) was added dropwise over 10 min. The suspension was allowed to warm to RT and stirred for 2 h. The reaction was quenched with Et_3N (6.70 mL, 48.2 mmol), then filtered through Celite® and concentrated *in vacuo* to give a yellow oil. Purification by chromatography (silica, 10% acetone/hexane + 1% Et_3N) afforded **BLS1** as a pale yellow amorphous

solid (16.6 g, 40.6 mmol, 84%, mixture of anomers *α/β* 1:5). A small fraction was further purified for analysis (*α/β* 7:93). **R$_f$:** 0.17 (30% EtOAc / hexane); **FT-IR** (neat) ν_{max}: 3364 (w), 3241 (w), 2962 (w), 2109 (s), 1744 (s), 1378 (m), 1213 (s), 1048 (s), 710 (w), 602 (w) cm^{-1}; **Data for major (β) anomer:** **^{1}H NMR** (500 MHz, $CDCl_3$) δ 5.00-4.92 (2H, m, H-3, H-4), 4.41 (1H, d, *J* = 8.1 Hz, H-1), 4.28 (1H, dd, *J* = 12.3, 4.9 Hz, H-6a), 4.12 (1H, dd, *J* =12.3, 2.4 Hz, H-6b), 4.07 (1H, dt, *J* = 10.0, 5.5 Hz, OC*H*HCH$_2$), 3.78 (1H, ddd, *J* = 10.0, 7.4, 5.0 Hz, OCH*H*CH$_2$), 3.71-3.64 (3H, m, H-5, CH$_2$C*H*$_2$Cl), 3.49 (1H, dd, *J* = 10.2, 8.1 Hz, H-2), 2.15 – 2.00 (2H, m, OCH$_2$C*H*$_2$), 2.09 (3H, s, Ac), 2.08 (3H, s,Ac), 2.02 (3H, s,Ac) ppm; **^{13}C{^{1}H} NMR** (126 MHz, $CDCl_3$) δ = 170.6 (*C*O, Ac), 169.9 (*C*O, Ac), 169.6 (*C*O, Ac), 102.2 (C-1), 72.3 (C-3), 71.8 (C-5), 68.4 (C-4), 67.0 (O*C*H$_2$CH$_2$), 63.8 (C-2), 61.9 (C-6), 41.4 (CH$_2$*C*H$_2$Cl), 32.4 (OCH$_2$*C*H$_2$), 20.7 (*C*H$_3$, Ac), 20.6 (*C*H$_3$, Ac), 20.5 (*C*H$_3$, Ac) ppm; **Selected data for minor (α) anomer: ^{1}H NMR** (500 MHz, $CDCl_3$) δ 3.32 (0.1H, dd, *J* = 10.8, 3.5 Hz, H-2) (Other signals obscured by major anomer) ppm; **LRMS** (ES$^+$) *m/z* 430 [M^{35}Cl+Na]$^+$; **HRMS** (ES$^+$) for $C_{15}H_{22}ClN_3NaO_8$ calcd 430.0988 found 430.0982

Synthesis of 3-chloropropyl 2-deoxy-2-azido-D-glucopyranoside (BLS2)

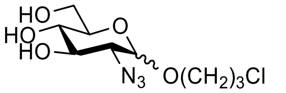

To a solution of **BLS1** (8.85 g, 21.7 mmol) in MeOH (100 mL) at 0 °C was added a solution of NaOMe in MeOH (25% wt, 1.99 mL, 8.68 mmol). The yellow solution was warmed to RT and stirred for 6 h. The reaction was neutralised using Amberlite® IR120 resin (pH 7), the resin beads filtered off and the solution concentrated *in vacuo*. Purification by chromatography (silica, 1% MeOH/EtOAc) afforded **BLS2** as a pale yellow oil (3.97 g, 14.1 mmol, 65%, Mixture of anomers *α/β* 1:10). **R$_f$** 0.35 (2% MeOH/ EtOAc); **FT-IR** (neat) ν_{max}: 3347 (br), 2886 (w), 2107 (s), 1262 (m), 1023 (s) cm^{-1}; **Data for major (β) anomer: ^{1}H NMR** (400 MHz, CD_3OD) δ 4.36 (1H, d, *J* = 8.0 Hz, H-1), 4.07 (1H, ddd, *J* = 10.1, 5.9, 5.3 Hz, OC*H*HCH$_2$), 3.78 (1H, dd, *J* = 11.9, 2.1 Hz, H-6a), 3.65 – 3.55 (4H, m, H-6b, OCH*H*CH$_2$, CH$_2$C*H*$_2$Cl), 3.36 – 3.23 (3H, m, H-3, H-4, H-5), 3.16 (1H, dd, *J* = 9.5, 8.2 Hz, H-2), 2.15 – 1.98 (2H, m, OCH$_2$C*H*$_2$) ppm; **^{13}C{^{1}H} NMR** (101 MHz, CD_3OD) δ 103.1 (C-1), 78.1 (C-5), 76.3 (C-3), 71.6 (C-4), 68.5 (C-2), 67.6 (O*C*H$_2$CH$_2$), 62.6 (C-6), 42.6 (CH$_2$*C*H$_2$Cl), 34.1 (OCH$_2$*C*H$_2$) ppm; **Selected data for minor (α) anomer: ^{1}H NMR** (400 MHz, CD_3OD) δ 4.90 (0.1H, d, *J* = 3.5 Hz, H-1), 3.08 (0.1H, dd, *J* = 10.4, 3.5 Hz, H-2) (Other signals obscured by major anomer) ppm; **LRMS** (ES$^+$) *m/z* 304 [M^{35}Cl+Na]$^+$; **HRMS** (ES$^+$) for $C_{19}H_{16}ClN_3NaO_5$ calcd 304.0671 found 304.0672.

Synthesis of 3-chloropropyl 2-deoxy-2-azido-3,4-*O*-[(2'*S*,3'*S*)-2',3'-dimethoxybutane-2',3'-diyl]-D-glucopyranoside (A, BLS3)

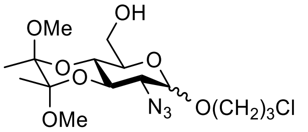

To a solution of **BLS2** (3.75 g, 13.3 mmol) in MeOH (40 mL) was added trimethyl orthoformate (8.00 mL, 73.2 mmol), butane-2,3-dione (1.38 mL, 16.0 mmol) and camphor sulfonic acid (309 mg, 1.33 mmol). The resulting yellow solution was heated to 80 °C and stirred at this temperature for 16 h, affording a dark red suspension. The reaction was cooled to RT, then neutralised with Et_3N (pH 7).

The reaction mixture was concentrated *in vacuo*. Purification by chromatography (silica, 20% acetone/hexane) afforded **BLS3** as an off-white amorphous solid (4.73 g, 12.0 mmol, 90%, mixture of anomers *α/β* 1:10). **R_f** 0.29 (30% acetone / hexane); **FT-IR** (neat) ν_{max}: 3524 (br, w), 2949 (w), 2109 (s), 1371 (m), 1289 (m), 1126 (s), 1025 (s) cm^{-1}; **Data for major (β) anomer: ^{1}H NMR** (500 MHz, $CDCl_3$): δ 4.35 (1H, d, *J* = 7.9 Hz, H-1), 4.05 (1H, ddd, *J* = 9.8, 6.1, 5.4 Hz, OC*H*HCH_2), 3.88 (1H, ddd, *J* = 12.0, 5.6, 2.9 Hz, H-6a), 3.79-3.74 (2H, m, H-6b, OCH*H*CH_2), 3.73-3.68 (3h, m, H-4, $CH_2C\mathit{H}_2Cl$), 3.62 (1H, dd, *J* = 10.4, 9.8 Hz, H-3), 3.50 (1H, ddd, *J* =9.7, 4.7, 2.9 Hz, H-5), 3.43 (1H, dd, *J* = 10.4, 7.9 Hz, H-2), 3.32 (3H, s, $OC\mathit{H}_3^{\mathbf{BDA}}$), 3.27 (3H, s, $OC\mathit{H}_3^{\mathbf{BDA}}$), 2.14-2.01 (2H, m, $OCH_2C\mathit{H}_2$), 1.92 (1H, dd, *J* = 8.0, 5.6 Hz, 6-O*H*), 1.35 (3H, s, $C\mathit{H}_3^{\mathbf{BDA}}$), 1.30 (3H, s, $C\mathit{H}_3^{\mathbf{BDA}}$) ppm; **^{13}C{^{1}H} NMR** ($CDCl_3$, 101 MHz) δ 102.6 (C-1), 100.0 ($\mathit{C}(CH_3)^{BDA}$), 99.7 ($\mathit{C}(CH_3)^{BDA}$), 74.0 (C-5), 70.6 (C-3), 66.6 ($O\mathit{C}H_2CH_2$), 66.0 (C-4), 62.8 (C-2), 61.1 (C-6), 48.0 ($O\mathit{C}H_3^{BDA}$), 48.0 ($O\mathit{C}H_3^{BDA}$), 41.5 ($CH_2\mathit{C}H_2Cl$), 32.5 ($OCH_2\mathit{C}H_2$), 17.6 ($\mathit{C}H_3^{BDA}$), 17.5 ($\mathit{C}H_3^{BDA}$) ppm; **Selected data for minor (α) anomer: ^{1}H NMR** (500 MHz, $CDCl_3$): δ 4.92 (0.1H, d, *J* = 3.7 Hz, H-1) (Other signals obscured by major anomer) ppm; **LRMS** (ES^+) *m/z* 418 $[M^{35}Cl+Na]^+$. **HRMS** (ES^+) for $C_{15}H_{26}ClN_3NaO_7$ calcd 418.1351 found 418.1358.

Synthesis of B and C

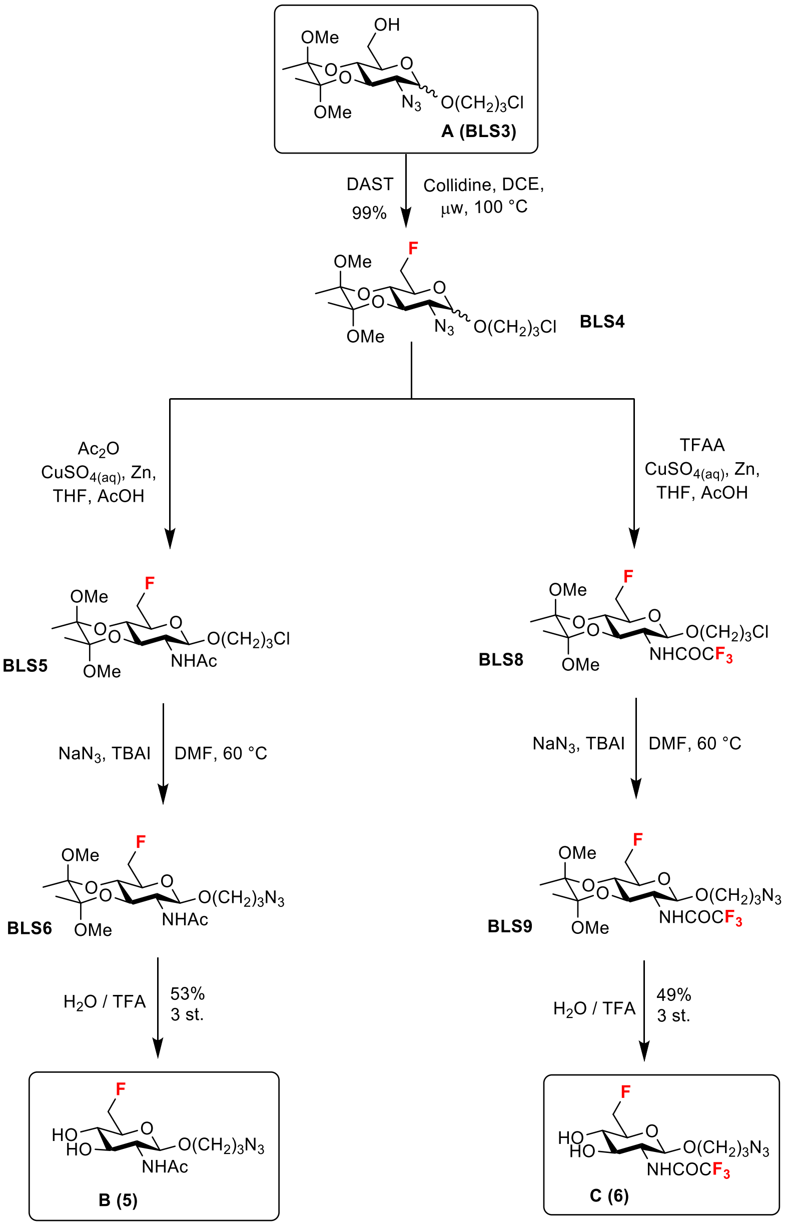

Synthesis of 3-Chloropropyl 2,6-dideoxy-2-azido-6-fluoro-3,4-*O*-[(2'*S*,3'*S*)-2',3'-dimethoxybutane-2',3'-diyl]-D-glucopyranoside (BLS4)

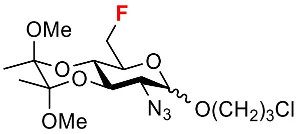

Into a microwave vial was added a solution of **BLS3** (1.63 g, 4.11 mmol) in DCE (13 mL), 2,4,6-trimethylpyridine (0.82 mL, 6.16 mmol) then DAST (0.76 mL, 6.16 mmol). The vial was capped then the solution reacted in a microwave reactor for 10 min at 100 °C, then quenched by the addition of MeOH, then concentrated *in vacuo*. Purification by chromatography (silica, 10% acetone/petroleum ether) afforded **BLS4** as an off-white solid (1.62 g, 4.06 mmol, 99%, mixture of anomers 7:93 α/β). $\mathbf{R_f}$

0.43 (15% acetone/hexane); **FT-IR** (neat) ν_{max}: 2955 (w), 2893 (w), 2113 (s), 1450 (w), 1368 (m), 1292 (m), 1127 (s), 1107 (s), 1014 (s), 958 (s) cm^{-1}; **Data for major (β) anomer: ^{1}H NMR ^{1}H NMR** (400 MHz, $CDCl_3$) δ 4.72 – 4.49 (2H, m, [*J* = 47.7 Hz can be extracted], H-6), 4.33 (1H, d, *J* = 7.7 Hz, H-1), 4.07 (1H, dt, *J* = 9.9, 5.5 Hz, OC*H*HCH$_2$), 3.79 – 3.52 (6H, m, H-3, H-4, H-5, OCH*H*CH$_2$, CH$_2$C*H*HCl), 3.46 (1H, dd, *J* = 10.8, 8.0 Hz, H-2), 3.32 (3H, s, OC*H*$_3^{BDA}$), 3.25 (3H, s, OC*H*$_3^{BDA}$), 2.19 – 2.00 (2H, m, OCH$_2$C*H*$_2$), 1.36 (3H, s, C*H*$_3^{BDA}$), 1.31 (3H, s, C*H*$_3^{BDA}$) ppm; **^{13}C{^{1}H} NMR** (101 MHz, $CDCl_3$) δ 102.8 (C-1), 100.1 (*C*(CH$_3$)^{BDA}), 99.9 (*C*(CH$_3$)^{BDA}), 80.6 (d, *J* = 174.6 Hz, C-6), 73.0 (d, *J* = 18.3 Hz, C-5), 70.5 (C-3), 66.8 (O*C*H$_2$CH$_2$), 64.9 (d, *J* = 7.3 Hz, C-4), 62.7 (C-2), 48.1 (O*C*H$_3^{BDA}$), 48.0 (d, *J* = 1.5 Hz, O*C*H$_3^{BDA}$), 41.6 (CH$_2$*C*H$_2$Cl), 32.5 (OCH$_2$*C*H$_2$), 17.6 (C(*C*H$_3$)^{BDA}), 17.5 (C(*C*H$_3$)^{BDA}) ppm; **^{19}F NMR** (376 MHz, $CDCl_3$) δ −235.76 (1F, td, *J* = 47.7, 26.0 Hz, F-6) ppm; **^{19}F{^{1}H} NMR** (376 MHz, $CDCl_3$) δ −235.59 (1F, s, F-6) ppm; **Selected data for minor (α) anomer: ^{1}H NMR** (400 MHz, $CDCl_3$) δ 4.95 (0.1H, d, *J* = 3.7 Hz, H-1) (Other signals obscured by major anomer) ppm; **^{19}F NMR** (376 MHz, $CDCl_3$) δ –236.55 - –237.17 (0.1F, m, F-6); **^{19}F{^{1}H} NMR** (376 MHz, $CDCl_3$) δ –236.79 (0.1F, s, F-6) ppm; **LRMS** (ES$^+$) *m/z* 420 [M^{35}Cl+Na]$^+$; **HRMS** (ES$^+$) for $C_{15}H_{25}ClFN_3NaO_6$ calcd 420.1308 found 420.1314.

Synthesis of 3-azidopropyl-2,6-dideoxy-2-acetimido-6-fluoro-β-D-glucopyranoside (B, 5)

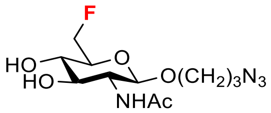

Using general procedure **A** with **BLS4** (450 mg, 1.13 mmol) and Ac_2O, **BLS5** was obtained as an off-white solid (468 mg, 1.13 mmol). Selected data: **FT-IR** (neat) ν_{max}: 3277 (w), 2953 (w), 1654 (m), 1555 (m), 1347 (m), 1110 (s), 1037 (s), 978 (m), 885 (m); **HRMS (ESI)** for $C_{17}H_{29}ClFNNaO_7$ (M + Na)$^+$ calcd 436.1509, found 436.1501. Then, a solution of **BSL5** (200 mg, 0.43 mmol), NaN_3 (152 mg, 2.35mmol) and TBAI (16 mg, 0.04 mmol) in DMF (6 mL) was heated at 60 ˚C for 15 h. The reaction was then diluted with EtOAc (100 mL) and brine (50 mL). The aqueous phase was separated and extracted with EtOAc (4 × 50 mL). The combined organic phases were washed with brine (2 × 100 mL), dried ($MgSO_4$), and concentrated *in vacuo* to afford crude **BLS6**. Selected data: **HRMS (ESI)** for $C_{17}H_{29}FN_4NaO_7$ calcd 443.1912 found 443.1919. The crude azide was then re-dissolved in H_2O (0.15 mL) and TFA (2.14 g, 1.4 mL, 18.8 mmol) at 0 ˚C. The suspension was then warmed to RT and concentrated *in vacuo*. Purification by chromatography (silica, 40% acetone/hexane) afforded **B** (**5)** as a white solid (145 mg, 0.40 mmol, 53% over 3 steps). **R$_f$**: 0.16 (60% acetone/CH_2Cl_2); **[α]$_D$** −13.2 (*c* 0.65, 26 °C, MeOH); **MP (obtained post chromatography)**: 138.5-140.4 °C; **FT-IR** (neat) ν_{max}: 3259 (br, m), 2949 (w), 2102 (s), 1649 (m), 1558 (m), 1371 (m), 1074 (s), 1013 (s), 954 (m), 909 (w) cm^{-1}; **^{1}H NMR** (500 MHz, CD_3OD) δ 4.64 (1H, ddd, *J* = 48.0, 10.2, 1.8 Hz, H-6a), 4.59 (1H, ddd, *J* = 47.5, 10.2, 4.4 Hz, H=6b), 4.42 (1H, d, *J* = 8.4 Hz, H-1), 3.91 (1H, ddd, *J* = 10.1, 6.0, 5.4 Hz, OC*H*HCH$_2$), 3.63 (1H, dd, *J* = 10.3, 8.4 Hz, H-2), 3.57 (1H, ddd, *J* = 10.1, 7.4, 5.2 Hz, OCH*H*CH$_2$), 3.50 – 3.33 (5H, m, H-3, H-4, H-5, CH$_2$C*H*$_2$N$_3$), 1.98 (3H, s, OC*H*$_3^{NHAc}$), 1.89 – 1.47 (2H, m, OCH$_2$C*H*$_2$) ppm; **^{1}H{^{19}F} NMR** (500 MHz, CD_3OD) δ 4.64 (1H, dd, *J* = 10.2, 2.0 Hz, H-6a), 4.59 (1H, dd, *J* = 10.2, 4.7 Hz, H-6b), 4.57 (1H, br s, N*H*Ac), 3.91 (1H, ddd, *J* = 10.0, 6.0, 5.2 Hz, OC*H*HCH$_2$), 3.63 (1H, dd, *J* = 10.3, 8.4 Hz, H-2), 3.57 (1H, ddd, *J* = 10.0, 7.4, 5.1 Hz, OCH*H*CH$_2$), 3.47 (1H, dd, *J* = 10.5, 8.8 Hz, H-3), 3.43 (1H, ddd, *J* = 10.0, 4.5, 1.8 Hz, H-5), 3.38 (2H, t, *J* = 6.7 Hz, CH$_2$C*H*$_2$N$_3$), 3.36 (1H, dd, *J*

= 8.8, 1.2 Hz, H-4), 1.98 (3H, s, OCH_3^{NHAc}), 1.89 – 1.74 (2H, m, OCH_2CH_2) ppm; **^{13}C{^{1}H} NMR** (126 MHz, CD_3OD) δ 173.9 (*C*O, NHAc), 103.0 (C-1), 83.4 (d, *J* = 171.7 Hz, C-6), 76.6 (d, *J* = 18.1 Hz, C-5), 75.9 (C-3), 71.0 (d, *J* = 7.2 Hz, C-4), 67.4 (OCH_2CH_2), 57.4 (C-2), 49.4 ($CH_2CH_2N_3$), 30.2 (OCH_2CH_2), 23.1 (OCH_3^{NHAc}) ppm; **^{19}F NMR** (470 MHz, CD_3OD) δ −235.7 (1F, td, *J* = 47.8, 24.1 Hz, F-6) ppm; **^{19}F{^{1}H} NMR** (470 MHz, CD_3OD) δ −235.7 (1F, s, F-6) ppm; **HRMS** (ES^+) for $C_{11}H_{19}FN_4NaO_5$ calcd 329.1232 found 329.1226.

Synthesis of 3-azidopropyl 2,6-dideoxy-2-trifluoroacetimido-6-fluoro-β-D-glucopyranoside (C, 6)

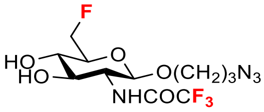

Using general procedure **A** with **BLS4** (2.62 g, 6.59 mmol) and TFAA, **BLS8** was obtained as an off-white amorphous solid (1.72 g, 3.68 mmol). Selected data: **FT-IR** (neat) ν_{max}: 3284 (w), 2952 (w), 1703 (s), 1564 (m), 1374 (m), 1172 (s), 1139 (s), 1106 (s), 1028 (s), 998 (s) cm^{-1}; **HRMS** (ES^+) for $C_{17}H_{26}ClF_4NNaO_7$ calcd 490.1266 found 490.1232. Then, using general procedure **B** with **BLS8** (2.20 g, 4.70 mmol), **BLS9** was obtained as an amorphous white solid (2.13 g, 4.49 mmol). Selected data: **HRMS** (ES^+) for $C_{17}H_{26}F_4N_4NaO_7$ calcd 497.1630 found 497.1640. Then, using general procedure **C** with **BLS9** (2.01 g, 4.24 mmol), **C** (**6**) was obtained as an amorphous white solid (1.41 g, 3.91 mmol, 49% over 3 steps). **R_f** 0.23 (40% acetone/hexane); **$[\alpha]_D$** −12.0 (*c* 0.52, 25 °C, MeOH); **MP (obtained psot chromatography)**: 150.6-152.0 °C; **FT-IR** (neat) ν_{max}: 3564 (w), 3279 (w), 3115 (w), 2113 (s), 1703 (s), 1564 (s), 1165 (s), 1068 (s) cm^{-1}; **^{1}H NMR** (500 MHz, CD_3OD) δ 4.65 (1H, ddd, J = 48.5, 10.8, 2.4 Hz, 1H, H-6a), 4.61 (1H, ddd, *J* = 47.8, 10.1, 4.5 Hz, H-6b), 4.50 (1H, d, *J* = 8.4 Hz, H-1), 3.92 (1H, ddd, *J* = 10.0, 6.1, 5.2 Hz, OC*H*HCH_2), 3.69 (1H, dd, *J* = 10.5, 8.4 Hz, H-2), 3.59 – 3.53 (2H, m, H-3, OCH*H*CH_2), 3.45 (1H, dddd, *J* = 24.3, 10.0, 4.5, 1.8 Hz, H-5), 3.40 – 3.34 (3H, m, H-4, $CH_2CH_2N_3$), 1.87 – 1.74 (2H, m, OCH_2CH_2) ppm; **^{13}C{^{1}H} NMR** (126 MHz, $MeOD_4$) δ 159.6 (q, J = 36.7 Hz, *C*O, NHTFA), 117.8 (q, *J* = 287.1 Hz, CF_3), 102.2 (C-1), 83.3 (d, *J* = 171.9 Hz, C-6), 76.7 (d, *J* = 18.1 Hz, C-5), 75.1 (C-3), 71.0 (d, *J* = 6.9 Hz, C-4), 67.5 (OCH_2CH_2), 57.8 (C-2), 49.3 ($CH_2CH_2N_3$), 30.2 (OCH_2CH_2) ppm; **^{19}F NMR** (470 MHz, $MeOD_4$) δ −77.3 (3F, s, CF_3), −235.8 (1F, td, *J* = 47.7, 24.2 Hz, F-6) ppm; **^{19}F{^{1}H} NMR** (470 MHz, $MeOD_4$) δ −77.23 (3F, s, CF_3), −235.8 (1F, s, F-6) ppm; **LRMS** (ES^+) *m/z* 383 $[M+Na]^+$; **HRMS** (ES^+) for $C_{11}H_{16}F_4N_4NaO_5$ calcd 383.0949 found 383.0953.

Synthesis of D and E

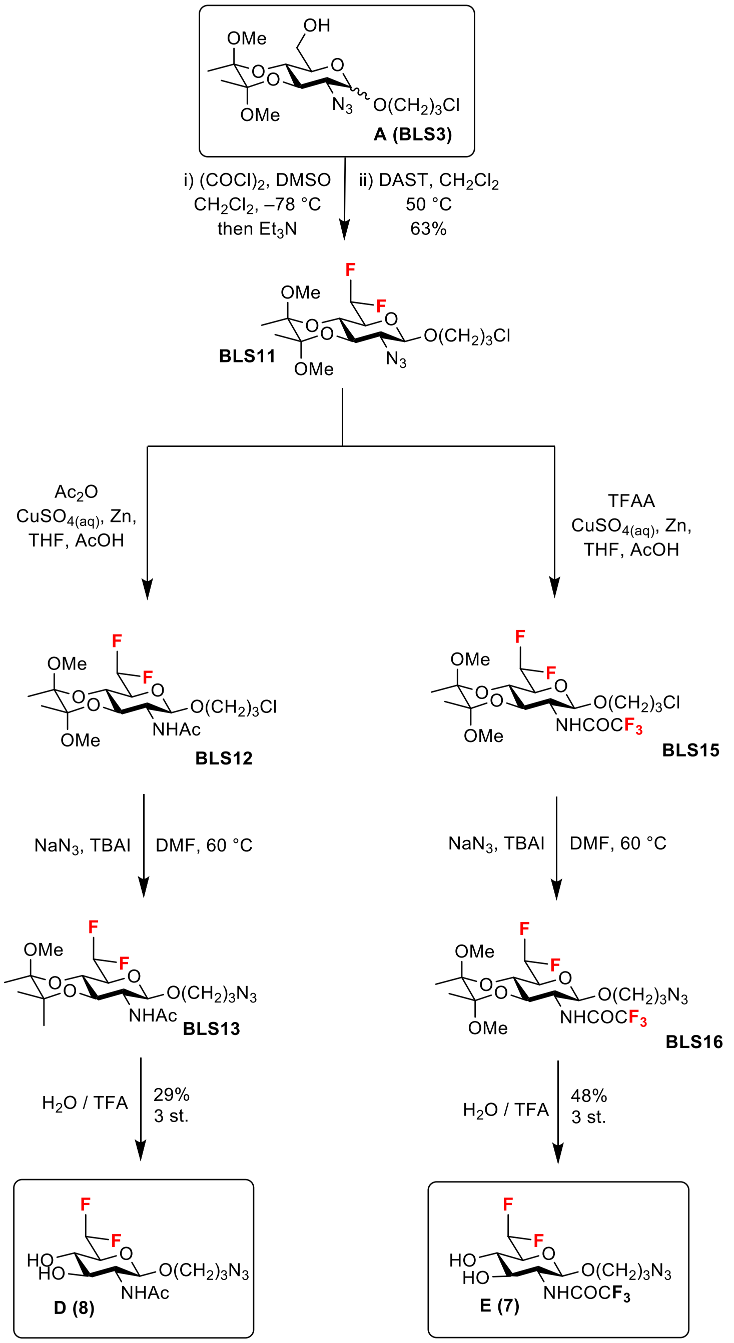

Synthesis of 3-Chloropropyl 2,6-dideoxy-2-azido-3,4-*O*-[(2'*S*,3'*S*)-2',3'-dimethoxybutane-2',3'-diyl]-6,6-difluoro-D-glucopyranoside (BLS11)

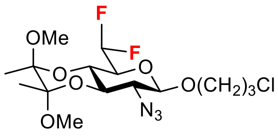

To a solution of DMSO (5.40 mL, 73.8 mmol) in CH_2Cl_2 (10 mL) at −78 °C was added $(COCl)_2$ (3.15 mL, 36.9 mmol) dropwise. The colourless solution was stirred at this temperature for 30 min. A solution of **BLS3** (3.65 g, 9.22 mmol) in CH_2Cl_2 (35 mL) was then added dropwise over 10 min. The

reaction was stirred at −78 °C for 2 h. Et_3N (20.0 mL, 148 mmol) was then added, the reaction warmed to RT and stirred for 16 h. The reaction was quenched by the addition of sat. $NaHCO_{3(aq)}$ (60 mL) and stirred for 30 min. The phases were separated and the aqueous phase extracted with CH_2Cl_2 (5 x 50 mL). The combined organic layers were washed with sat. $NaHCO_{3(aq)}$ (200 mL), dried ($MgSO_4$) and concentrated *in vacuo* to afford the crude aldehyde as a colourless oil. The residue was re-dissolved in DCE (45 mL). DAST (6.09 mL, 46.1 mmol) was added dropwise, and the yellow solution heated to 50 °C for 16 h. The reaction was cooled to RT, diluted with CH_2Cl_2 (50 mL) and poured into vigorously stirred sat. $NaHCO_{3(aq)}$ (100 mL) and stirred for 30 min. The aqueous phase was separated and extracted with CH_2Cl_2 (4 x 100 mL). The combined organic layers were washed with sat. $NaHCO_{3(aq)}$ (500 mL), dried ($MgSO_4$) and concentrated to afford an off-yellow solid. Purification by chromatography (silica, 10% acetone/hexane) afforded **BLS11** as an off-yellow amorphous solid (2.40 g, 5.77 mmol, 63%, mixture of anomers α/β 2:98). **R_f** 0.43 (15% acetone/hexane); **FT-IR** (neat) ν_{max}: 2967 (w), 2843 (w), 2114 (s), 1388 (m), 1293 (m), 1133 (s), 1031 (s) cm^{-1}; **Data for major (β) anomer:** **^{1}H NMR** (400 MHz, $CDCl_3$) δ 5.94 (1H, dt, *J* = 53.6, 1.3 Hz, H-6), 4.35 (1H, d, *J* = 7.7, H-1), 4.07 (1H, dt, *J* = 10.2, 5.8 Hz, OC*H*HCH₂), 3.84 (1H, t, *J* = 10.0 Hz, H-4), 3.80 – 3.58 (5H, m, H-3, H-5, OCH*H*CH₂, CH₂C*H₂*Cl), 3.46 (1H, dd, *J* = 10.6, 7.8 Hz, H-2), 3.31 (3H, s, OCH_3^{BDA}), 3.26 (3H, s, OCH_3^{BDA}), 2.19 – 1.99 (2H, m, OCH₂C*H₂*), 1.35 (3H, s, CH_3^{BDA}), 1.30 (3H, s, OCH_3^{BDA}) ppm; **^{13}C{^{1}H} NMR** (101 MHz, $CDCl_3$) δ 112.5 (t, *J* = 244.7 Hz, C-6), 103.0 (C-1), 100.2 ($C(CH_3)^{BDA}$), 99.9 ($C(CH_3)^{BDA}$), 71.9 (t, *J* = 21.3 Hz, C-5), 70.1 (C-3), 67.0 (O*C*H₂CH₂), 65.6 (t, *J* = 3.7 Hz, C-4), 62.5 (C-2), 48.1 (OCH_3^{BDA}), 48.1 (OCH_3^{BDA}), 41.5 (CH₂*C*H₂Cl), 32.4 (OCH₂*C*H₂), 17.6 (CH_3^{BDA}), 17.4 (CH_3^{BDA}) ppm; **^{19}F NMR** (376 MHz, $CDCl_3$) δ −133.54 (2F, dd, *J* = 53.6, 12.1 Hz, CH*F₂*) ppm; **^{19}F{^{1}H} NMR** (376 MHz, $CDCl_3$) δ −133.62 (2F, s, CH*F₂*) ppm; **Selected data for minor (α) anomer:** **^{1}H NMR** (400 MHz, $CDCl_3$) δ 5.98 (0.1H, td, *J* = 53.4, 1.1 Hz, H-6), 4.99 (0.1H, d, *J* = 3.7 Hz, H-1) (Other signals obscured by major anomer) ppm; **LRMS** (ES^+) *m/z* 384 $[M–OMe]^+$; **HRMS** (ES^+) for $C_{15}H_{24}ClF_2NaN_3O_6$ calcd 438.1214 found 438.1218.

Synthesis of 3-azidopropyl-2,6-dideoxy-2-acetimido-6,6-difluoro-β-D-glucopyranoside (D, 8)

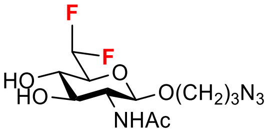

Using general procedure **A** with **BLS11** (500 mg, 1.20 mmol) and Ac_2O, **BLS12** was obtained as an off-white solid (500 mg, 1.16 mmol). **FT-IR** (neat) ν_{max}: 2924 (br, w), 1749 (s), 1654 (s), 1375 (m), 1224 (m), 1140 (s), 1038 (s), 931 (w), 886 (w) cm^{-1}; **HRMS (ESI)** for $C_{17}H_{28}ClF_2NNaO_7$ calcd 454.1415 found 454.1420. Then, using general procedure **B** with **BLS12** (500 mg, 1.16 mmol), **BLS13** was obtained as an off-white solid (458 mg, 1.05 mmol). Selected data: **HRMS (ESI)** for $C_{17}H_{28}F_2N_4NaO_5$ calcd 461.1818 found 461.1826. Then, using general procedure **C** with **BLS13** (458 mg, 1.04 mmol), **D** (**8**) was obtained as a pale yellow solid (114 mg, 0.35 mmol, 29% over 3 steps). **R_f**: 0.18 (60% acetone/CH_2Cl_2); **$[\alpha]_D$** −12.7 ° (*c* 0.15, 26 °C, MeOH); **MP (obtained post chromatography)**: 120.1-122.3 °C; **FT-IR** (neat) ν_{max}: 3393 (br, m), 2917 (w), 1629 (m), 1542 (m), 1374 (m), 1153 (s), 1061 (s), 1022 (s), 971 (m), 887 (m) cm^{-1}; **^{1}H NMR** (500 MHz, CD_3OD) δ 6.07 (1H, td, *J* = 53.9, 1.2 Hz, H-6), 4.48 (1H, d, *J* = 8.3 Hz, H-1), 3.93 (1H, ddd, *J* = 10.0, 6.0, 5.1 Hz, OC*H*HCH₂), 3.65 (1H, dd, *J* = 9.9, 8.3 Hz, H-2), 3.60 (1H, ddd, *J* = 10.1, 7.9, 5.2 Hz, OCH*H*CH₂),

3.57 – 3.44 (3H, m, H-3, H-4, H-5), 3.40 (2H, t, *J* = 6.7 Hz, $CH_2C\mathit{H}_2Cl$), 2.00 (3H, s, $OC\mathit{H}_3^{NHAc}$), 1.92 – 1.76 (2H, m, $OCH_2C\mathit{H}_2$) ppm; **^{1}H{^{19}F} NMR** (500 MHz, CD_3OD) δ 6.05 (1H, d, *J* = 1.2 Hz, H-6), 4.46 (1H, d, *J* = 8.4 Hz, H-1), 3.91 (1H, ddd, *J* = 10.0, 6.0, 5.1 Hz, $OC\mathit{H}HCH_2$), 3.63 (1H, dd, *J* = 9.8, 8.5 Hz, H-2), 3.58 (1H, ddd, *J* = 9.4, 7.3, 4.9 Hz, $OCH\mathit{H}CH_2$), 3.53 – 3.43 (3H, m, H-3, H-4, H-5), 3.38 (2H, t, *J* = 6.7 Hz, 2H, $CH_2C\mathit{H}_2Cl$), 1.98 (3H, s, $OC\mathit{H}_3^{NHAc}$), 1.90 – 1.75 (2H, m, $OCH_2C\mathit{H}_2$). **^{13}C NMR** (126 MHz, CD_3OD) δ 173.7 (*C*O), 115.1 (t, *J* = 241.3 Hz, C-6), 103.1 (C-1), 75.5 (C-3) , 75.3 (t, *J* = 20.0 Hz, C-5), 71.1 (d, *J* = 4.9 Hz, C-4), 67.5 ($O\mathit{C}H_2CH_2$), 57.1 (C-2), 49.4 ($CH_2\mathit{C}H_2N_3$ [assigned from 2D data]) 30.1 ($OCH_2\mathit{C}H_2$), 23.0 ($O\mathit{C}H_3^{NHAc}$) ppm; **^{19}F NMR** (471 MHz, CD_3OD) δ -133.7 (1F, ddd, *J* 286.1, 53.9, 6.9 Hz, F-6), -136.2 (1F, ddd, *J* 286.4, 54.0, 17.7 Hz, F-6) ppm; **^{19}F{^{1}H} NMR** (471 MHz, CD_3OD) δ -133.7 (1F, d, *J* 286.3 Hz, F-6), -136.2 (1F, d, *J* 286.2 Hz, F-6) ppm; **HRMS** (ES^+) for $C_{11}H_{18}F_2N_4NaO_5$ calcd 347.1137 found 347.1144.

Synthesis of 3-azidopropyl 2,6-dideoxy-2-trifluoroacetimido-6,6-dfluoro-β-d-glucopyranoside (E, 7)

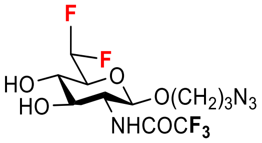

Using general procedure **A** with **BLS11** (461 mg, 1.11 mmol) and TFAA, **BLS15** was obtained as an off-white powder (318 mg, 0.65 mmol). Selected data: **FT-IR** (neat) ν_{max}: 3293 (w), 2968 (w), 1703 (s), 1561 (m), 1140 (s), 1024 (s) cm^{-1}; **HRMS** (ES^+) for $C_{17}H_{25}ClF_5NNaO_7$ calcd 508.1137 found 508.1132. Then, using general procedure **B** with **BLS15** (1.50 g, 3.09 mmol), **BLS16** was obtained as an off-white powder (1.38 g, 2.80 mmol). Selected data: **HRMS** (ES^+) for $C_{17}H_{25}F_5N_4NaO_7$ calcd 515.1541 found 515.1550. Then, using general procedure **C** with **BLS16** (1.28 g, 2.60 mmol), **E (7)** was obtained as an off-white powder (876 mg, 2.32 mmol, 48% over 3 steps). **R$_f$** 0.40 (40% acetone/hexane); **[α]$_D$** −11.0 ° (*c* 0.53, 25 °C, MeOH); **MP** (obtained post chromatography): 129.3-131.6 °C; **FT-IR** (neat) ν_{max}: 3278 (br), 2940 (w), 2102 (s), 1703 (w), 1563 (w), 1366 (m), 1165 (s), 1036 (s), 882 (m) cm^{-1}; **^{1}H NMR** (500 MHz, CD_3OD) δ 6.06 (1H, td, *J* = 53.9, 1.4 Hz, H-6), 4.53 (1H, d, *J* = 8.4 Hz, H-1), 3.92 (1H, ddd, *J* = 10.0, 5.9, 5.2 Hz, $OC\mathit{H}HCH_2$), 3.69 (1H, dd, *J* = 10.4, 8.4 Hz, H-2), 3.58 (1H, ddd, *J* = 10.1, 7.6, 5.0 Hz, $OCH\mathit{H}CH_2$), 3.58 (1H, dd, *J* = 10.5, 8.7 Hz, H-3), 3.55 – 3.44 (2H, m, H-4, H-5), 3.36 (2H, td, *J* = 7.0, 1.4 Hz, $CH_2C\mathit{H}_2N_3$), 1.89 – 1.73 (2H, m, $OCH_2C\mathit{H}_2$) ppm; **^{1}H{^{19}F} NMR** (500 MHz, CD_3OD) δ 6.06 (1H, d, *J* = 1.3 Hz, H-6), 4.54 (1H, d, *J* = 8.3 Hz, H-1), 3.92 (1H, ddd, *J* 10.0, 6.0, 5.2 Hz, $OC\mathit{H}HCH_2$), 3.69 (1H, dd, *J* 10.4, 8.4 Hz, H-2), 3.58 (1H, ddd, *J* 10.1, 7.5, 5.0 Hz, 1H, $OCH\mathit{H}CH_2$), 3.58 (1H, dd, *J* 10.5, 8.6 Hz, H-3), 3.53 (1H, dd, *J* 9.9, 1.2 Hz, H-5), 3.47 (1H, dd, *J* 9.9, 8.4 Hz, H-4), 3.36 (2H, td, *J* 6.8, 0.9 Hz, $CH_2C\mathit{H}_2N_3$), 1.89 – 1.73 (2H, m, $OCH_2C\mathit{H}_2$) ppm; **^{13}C{^{1}H} NMR** (126 MHz, CD_3OD) δ 160.3 (q, *J* = 36.7 Hz, *C*O), 118.4 (q, *J* = 286.8 Hz, $\mathit{C}F_3$), 115.9 (t, *J* = 242.5 Hz, C-6), 103.2 (C-1), 76.2 (t, *J* = 19.9 Hz, C-5), 75.5 (C-3), 72.0 (d, *J* = 5.0 Hz, C-4), 68.5 ($O\mathit{C}H_2CH_2$), 58.3 (C-2), 50.0 ($CH_2\mathit{C}H_2N_3$), 30.9 ($OCH_2\mathit{C}H_2$) ppm; **^{19}F NMR** (471 MHz, CD_3OD) δ −77.3 (3F, s, $C\mathit{F}_3$), −133.7 (1F, ddd, *J* = 286.7, 53.8, 7.0 Hz, F-6a), −136.2 (1F, ddd, *J* = 287.1, 53.9, 17.6 Hz, F-6b) ppm; **^{19}F{^{1}H} NMR** (471 MHz, CD_3OD) δ −77.3 (3F, s, $C\mathit{F}_3$), −133.7 (1F, d, *J* = 286.7 Hz, F-6a), −135.32 (1F, d, *J* = 287.1 Hz, F-6b) ppm; **LRMS** (ES^+) *m/z* 401 [M+Na]$^+$; **HRMS** (ES^+) for $C_{11}H_{15}F_5N_4NaO_5$ calcd 401.0860 found 401.0852.

Synthesis of 3-Azidopropyl 2-acetamido-2-deoxy-*β*-D-glucopyranoside 14

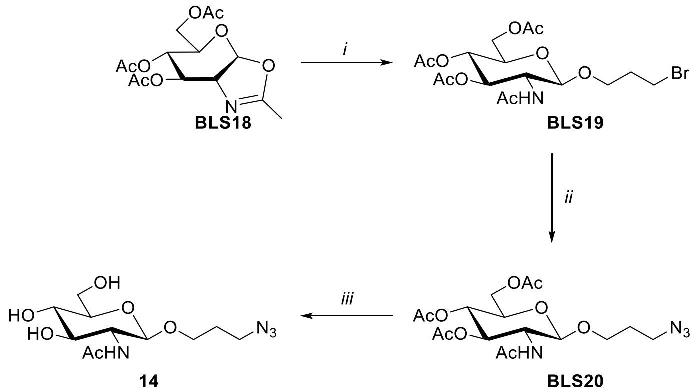

Scheme S1: Reagents and conditions for the synthesis of **14**: i) 3-bromopropan-1-ol, $CuBr_2$, THF, rt, 16 h (57%); ii) NaN_3, DMF, 80°C, 16h (63%); iii) MeONa 2M, MeOH, 1h (92%).

3-Bromopropyl 2-acetamido-2-deoxy-3,4,6-tri-*O*-acetyl-*β*-D-glucopyranoside BLS19

To a solution of oxazoline **BLS18** [5] (4.3 g, 12.9 mmol) and 3-bromopropan-1-ol (3.0 mL, 64.7 mmol) in THF (40 mL) at rt under N_2, was added $CuBr_2$ (2.9 g, 12.9 mmol). The resulting mixture was warmed to 50°C and left stirring for 16h. The mixture was then cooled to rt, filtered through celite and then resuspended in EtOAc and washed with HCl 1M followed by a saturated solution of $NaHCO_3$ before drying over anhydrous $MgSO_4$. The solution was then concentrated under reduced pressure and the dried residue purified by flash column chromatography (9:1 to 8:1 DCM:Acetone) to give **BLS19** (3.5 g, 57%) as a white solid. **^{1}H NMR** (400 MHz, $CDCl_3$) δ 5.52 (d, *J* = 9.0 Hz, 1H, NHAc), 5.20 (dd, *J* = 10.6, 9.7 Hz, 1H, H-3), 5.08 (dd, *J* = 9.7, 9.4 Hz, 1H, H-4), 4.58 (d, *J* = 8.3 Hz, 1H, H-1), 4.26 (dd, *J* = 12.4, 4.7 Hz, 1H, H-6a), 4.14 (dd, *J* = 12.4, 2.0 Hz, 1H, H-6b), 4.03 – 3.91 (m, 2H, H-2, OC*H*HCH$_2$), 3.71 – 3.63 (m, 2H, H-5, OCH*H*CH$_2$), 3.52 – 3.47 (m, 2H, CH$_2$C*H*$_2$Br), 2.22 – 2.11 (m, 2H, OCH$_2$C*H*$_2$), 2.09 (s, 3H, CH$_3$, Ac), 2.04 (s, 3H, CH$_3$, Ac), 2.03 (s, 3H, CH$_3$, Ac), 1.97 (s, 3H, CH$_3$, NHAc); **^{13}C NMR** (101 MHz, $CDCl_3$) δ 171.2 (*C*O, NHAc), 170.8 (*C*O, Ac), 170.3 (*C*O, Ac), 169.5 (*C*O, Ac), 101.7 (C-1), 72.6 (C-3), 72.1 (C-5), 68.6 (C-4), 67.4 (O*C*H$_2$CH$_2$), 62.2 (C-6), 54.6 (C-2), 32.2 (CH$_2$*C*H$_2$Br), 30.7 (OCH$_2$*C*H$_2$), 23.5 (CH$_3$, NHAc), 20.9 (CH$_3$, Ac), 20.8, 20.8 (2 x CH$_3$, Ac). Characterization data in agreement with literature. [6]

3-Azidopropyl 2-acetamido-2-deoxy-3,4,6-tri-*O*-acetyl-*β*-D-glucopyranoside BLS20

To **BLS19** (5.00 g, 10.7 mmol) dissolved in DMF (110 mL) at rt, was added NaN_3 (2.08 g, 32.0 mmol). The resulting solution was stirred for 16h at 80°C. The crude product was purified by flash column chromatography (98:2 DCM:MeOH) to give **BLS20** (4.96 g, 85%) as a pale orange solid. **^{1}H NMR** (400 MHz, $CDCl_3$) δ 5.48 (d, *J* = 8.9 Hz, 1H, NHAc), 5.23 (dd, *J* = 10.7, 9.3 Hz, 1H, H-3), 5.07 (dd, *J* = 9.9, 9.3 Hz, 1H, H-4), 4.61 (d, *J* = 8.3 Hz, 1H, H-1), 4.25 (dd, J = 12.3, 4.8 Hz, 1H, H-6a), 4.14 (dd, *J* = 12.3, 2.5 Hz, 1H, H-6b), 4.00 – 3.84 (m, 2H, H-2, $OCHHCH_2$), 3.69 (ddd, *J* = 9.9, 4.8, 2.5 Hz, 1H, H-5), 3.59 (app. ddd, *J* = 9.8, 8.2, 4.8 Hz, 1H, $OCHHCH_2$), 3.45 – 3.27 (m, 2H, $CH_2CH_2N_3$), 2.09 (s, 3H, CH_3, Ac), 2.03 (s, 3H, CH_3, Ac), 2.03 (s, 3H, CH_3, Ac), 1.96 (s, 3H, CH_3, NHAc), 1.94 – 1.70 (m, 2H, OCH_2CH_2); **^{13}C NMR** (101 MHz, $CDCl_3$) δ 171.1 (*C*O, NHAc), 170.8 (*C*O, Ac), 170.2 (*C*O, Ac), 169.5 (*C*O, Ac), 101.2 (C-1), 72.5 (C-3), 72.1 (C-5), 68.7 (C-4), 66.4 (OCH_2CH_2), 62.2 (C-6), 54.7 (C-2), 48.2 ($CH_2CH_2N_3$), 29.1 (OCH_2CH_2), 23.5 (CH_3, NHAc), 20.9 (CH_3, Ac), 20.8 (CH_3, Ac), 20.8 (CH_3, Ac). Characterization data in agreement with literature. [7,8]

3-Azidopropyl 2-acetamido-2-deoxy-*β*-D-glucopyranoside 14

To a solution of **BLS20** (600 mg, 1.39 mmol) in methanol (15 mL), a 2M solution of sodium methoxide in methanol (0.6 mL, 1.2 mmol) was added and the mixture was stirred at room temperature for 1h. Reaction was neutralised with Amberlite IR-120 resin until neutral pH of the solution, the resin was filtered off and solvent was evaporated under reduced pressure. Compound **14** (390 mg, 92%) was obtained as white solid and was used without further purification. **^{1}H NMR** (400 MHz, D_2O) δ 4.50 (d, *J* = 8.5 Hz, 1H, H-1), 3.96 (dt, *J* = 10.3, 5.6 Hz, 1H, $OCHHCH_2$), 3.91 (dd, *J* = 12.4, 1.8 Hz, 1H, H-6a), 3.78 -3.60 (m, 3H, H-6b, $OCHHCH_2$, H-2), 3.53 (t, *J* = 9.5 Hz, 1H, H-3), 3.46-3.40 (m, 2H, H-4, H-5), 3.36 (td, *J* = 6.6, 1.7 Hz, 2H, $CH_2CH_2N_3$), 2.04 (s, 3H, CH_3, NHAc), 1.83 (p, *J* = 6.6 Hz, 1H, OCH_2CH_2). **^{13}C NMR** (101 MHz, D_2O) δ 174.4 (*C*O, NHAc), 101.1 (C-1), 75.7 (C-5), 73.6 (C-3), 69.8 (C-4), 67.0 (OCH_2CH_2), 60.6 (C-6), 55.5 (C-2), 47.7 ($CH_2CH_2N_3$), 28.0 (OCH_2CH_2), 22.0 (CH_3, NHAc). Characterization data in agreement with literature[9]

Enzymatic synthesis of lacto-n-biose analogues

Gal-β(1,3)-GlcNAc-N_3 (15)

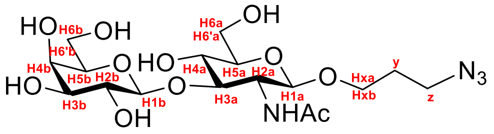

Reactions were assembled by adding GlcNAc-N_3 (**14**, 77 mg), Gal (**9**, 88.77 mg) and ATP (407 mg) in 4 mL of ddH_2O, to 1644 µL of 1 M Tris buffer (pH 6.5) and 328 µL of 1 M $MgCl_2$ in a 50 mL falcon tube. After adjusting the pH to 6.5, 10.7 mg of BiGalK and 7.8 mg of BiGalHexNAcP was added and the reaction was made up to 16.44 mL with ddH_2O. After incubation at 37 °C with shaking (120 rpm) for 90 h, the enzymes were removed by ultrafiltration (Vivaspin Protein Concentrator Spin Column 30000 MWCO). The reaction was lyophilised, resuspended in ddH_2O and purified by BioGel P2 column chromatography in H_2O. Fractions containing the desired product (**19**) were dried onto silica gel and further purified by flash chromatography (EtOAc: MeOH: H2O = 4:1:0.1). Fractions containing the desired product (**15**) were pooled and evaporated *in vacuo* (55.2 mg, 47 %). Characterisation data in agreement with the literature.[9] Appearance: white powder. **ATR-FTIR** V_{max} (thin film/cm^{-1}) 3305, 2881, 2410, 2098, 1948, 1614, 1424, 1372, 1300, 1116, 1077, 1077, 1029, 892, 779, 618, 551. **HRMS** (ESI) *m/z* calcd. for $C_{17}H_{31}N_4O_{11}$ (M + H) 467.1987 found 467.1986. **^{1}H NMR (500 MHz, D_2O)** δ 4.58 (d, *J* = 8.1 Hz, 1H, **H-1b**), 4.46 (d, *J* = 7.8 Hz, 1H, **H-1a**), 4.04 – 3.93 (m, 3H), 3.86 – 3.65 (m, 8H), 3.59 – 3.50 (m, 3H), 3.41 (td, *J* = 6.6, 2.4 Hz, 2H, **H-z**), 2.07 (s, 3H, **NHCOC<u>H</u>$_3$**), 1.91 – 1.85 (m, 2H, **H-y**). **^{13}C NMR (125 MHz, D_2O)** δ 174.54 (**C=O**), 103.46 (**C-1a**), 100.87 (**C-1b**), 82.34, 75.30, 75.22, 72.42, 70.61, 68.66, 68.46, 67.10 (**C-x**), 60.95, 60.67, 54.51, 47.73 (**C-z**), 28.04 (**C-y**), 22.16 (**NHCO<u>C</u>H$_3$**).

Gal3F-β(1,3)-GlcNAc-N_3 (16)

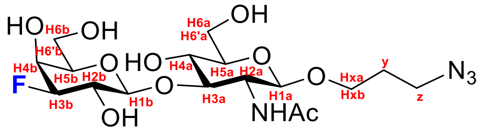

Reactions reactions were assembled by adding GlcNAc-N_3 (**14**, 10.7 mg), Gal3F (**10**, 56.4 mg) and ATP (90.2 mg) in 3 mL ddH_2O, to 1.09 mL of 1 M Tris buffer (pH 6.5) and 218 µL of 1 M $MgCl_2$ in a 50 mL falcon tube. After adjusting the pH to 6.5, 21.8 mg of BiGalK and 21.8 mg of BiGalHexNAcP were added and the reaction was made up to 10.9 mL with ddH_2O and incubated at 37 °C with shaking (120 rpm). After 50 h, 9 mg of BiGalK and 19 mg of BiGalHexNAcP were added and the reaction was further incubated at 37 °C with shaking (120 rpm). After 64 h, 8 mg of BiGalHexNAcP was added and the reaction was further incubated at 37 °C with shaking (120 rpm). After 120 h, 19 mg of BiGalHexNAcP was added and the reaction was further incubated at 37 °C with shaking (120 rpm). After 144 h, the enzymes were removed by ultrafiltration (Vivaspin Protein Concentrator Spin Column 30000 MWCO). The reaction was lyophilised, resuspended in ddH_2O and purified by BioGel P2 column chromatography in H_2O. Fractions containing the desired product (**16**) were pooled and further purified by anion exchange chromatography using a HiTrap Q anion exchange column. The column was washed with 5 CV of H_2O. The flow through and wash fractions containing the desired product (**16**, 8 mg, 48 %) were retained and lyophilised. The bound ADP was eluted from the HiTrap Q anion

exchange column with 2 CV of 2M $(NH_4)HCO_3$ solution. Appearance: yellow solid. **ATR-FTIR** V_{max} (thin film/cm^{-1}) 3300, 2106, 1673, 1360, 1204, 1134, 1078, 833, 801, 701. **HRMS** (ESI) *m/z* calcd for $C_{17}H_{29}FN_4NaO_{10}$ (M + Na) 491.1760, found 491.1769. **^{1}H NMR (500 MHz, D_2O)** δ 4.59 (m, 1H, **H-3b**), 4.58 (d, *J* = 8.2 Hz, 1H, **H-1b**), 4.51 (d, *J* = 7.8 Hz, 1H, **H-1a**), 4.25 – 4.22 (m, 1H, **H-4b**), 4.01 (dt, *J* = 10.4, 5.6 Hz, 1H, **H-xa**), 3.96 (dd, *J* = 12.4, 2.2 Hz, 1H, **H-6a**), 3.87 – 3.68 (m, 8H, **H-2b, H-2a, H-4a, H-5b, H-6a',H-6b, H-6b', H-xb**), 3.58 (dd, *J* = 10.0, 8.2 Hz, 1H, **H-3a**), 3.51 (ddd, *J* = 9.9, 5.6, 2.3 Hz, 1H, **H-5a**), 3.41 (td, *J* = 6.6, 2.3 Hz, 2H, **H-z**), 2.06 (s, 3H, **NHCOC<u>H</u>$_3$**), 1.91 – 1.84 (m, 2H, **H-y**). **^{13}C NMR (125 MHz, D_2O)** δ 174.56 (**C=O**), 102.77 (**C-1a**), 100.83 (**C-1b**), 93.39 (**C-3b**), 82.40, 73.92, 69.34, 69.19, 60.65 & 54.52 (**C-2a, C-2b, C-4a, C-5b, C-6a, C-6b**), 75.30 (**C-5a**), 68.60 (**C-3a**), 67.11 (**C-x**), 66.67 (**C-4b**), 47.73 (**C-x**), 28.03 (**C-y**), 22.13 (**NHCO<u>C</u>H$_3$**). **^{19}F {^{1}H} (500 MHz, D_2O)** δ -199.00.

Gal6F-β(1,3)-GlcNAc-N_3 (17)

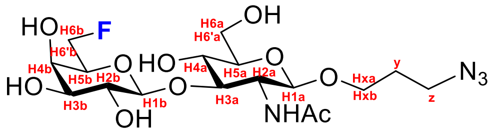

Reactions were assembled by adding GlcNAc-N_3 (**14**, 58 mg), Gal6F (**11**, 47 mg) and ATP (160 mg) in 4 mL of ddH_2O, to 1009 µL of 1 M Tris buffer (pH 6.5) and 218 µL of 1 M $MgCl_2$ in a 50 mL falcon tube. After adjusting the pH to 6.5, 6 mg of BiGalK and 4 mg of BiGalHexNAcP were added and the reaction was made up to 10.09 mL in ddH_2O. The reaction was incubated at 37 °C with shaking (120 rpm). After 72 h, a further 5.4 mg of BiGalHexNAcP was added and the reaction was incubated at 37 °C. After 79 h, 5 mg of BiGalK and 5.4 mg of BiGalHexNAcP were added and the reaction was incubated at 37 °C. After 97 h, another 10 mg of BiGalHexNAcP was added and the reactions was incubated at 37 °C for a further 24 h. The enzymes were removed by ultrafiltration (Vivaspin Protein Concentrator Spin Column 30000 MWCO). The reaction was lyophilised and resuspended in dH_2O, dried onto silica gel and purified by flash chromatography (EtOAc: MeOH: H_2O = 4:1:0.1). Fractions containing the desired product (**17**) were pooled and evaporated *in vacuo*. The compound was further purified by BioGel P2 column chromatography in H_2O. Fractions containing the pure desired product (**17**) were pooled and lyophilised (30.8 mg, yield 47 %). Yield calculated based on the recovery of unreacted GlcNAc-N_3 (14.4 mg) starting material. Appearance: white powder. **ATR-FTIR** V_{max} (thin film/cm^{-1}) 3321, 2889, 2099, 1632, 1424, 1372, 1304, 1127, 1076, 1029, 897, 781, 702, 597. **HRMS** (ESI) *m/z* calcd for $C_{17}H_{30}FN_4O_{10}$ (M + H) 469.1940, found 469.1952. **^{1}H NMR (500 MHz, D_2O)** δ 4.77 – 4.69 (m, 1H, **H-6b**), 4.68 – 4.61 (m, 1H, **H-6'b**), 4.59 (d, *J* = 8.4 Hz, 1H, **H-1b**), 4.48 (d, *J* = 7.8 Hz, 1H, **H-1a**), 4.07 – 3.98 (m, 3H, **H-5a, H-3a & H-xa**), 3.95 (dd, *J* = 12.3, 2.2 Hz, 1H, **H-6a**), 3.86 (dd, *J* = 10.4, 8.4 Hz, 1H, **H-2b**), 3.82 – 3.76 (m, 2H, **H-6'a & H-4b**), 3.74 – 3.68 (m, 2H, **H-xb & H-3a**), 3.58 (dd, *J* = 9.8, 8.0 Hz, 2H, **H-2a** and **H-4a**), 3.51 (ddd, *J* = 9.9, 5.6, 2.2 Hz, 1H, **H-5a**), 3.41 (td, *J* = 6.7, 2.2 Hz, 2H, **H-z**), 2.07 (s, 3H, **NHCOC<u>H</u>$_3$**), 1.91 – 1.85 (m, 2H, **H-y**). **^{13}C NMR (125 MHz, D_2O)** δ 174.55 (**NH<u>C</u>OCH$_3$**), 103.61 (**C-1b**), 100.85 (**C-1a**), 83.00 (d, *J* = 165.4 Hz, **C-6b**), 83.39 (**C-4b**), 75.19 (**C-5a**), 73.39 (d, *J* = 19.6 Hz, **C-5b**), 72.24 (**C-3a**), 70.37 & 68.70 (**C-2a & C-4a**), 67.96 (d, *J* = 7.4 Hz, **C-3b**), 67.11 (**C-x**),60.62 (**C-6a**), 54.34 (**C-2b**), 47.74 (**C-z**), 28.04 (**C-y**), 22.14 (**NHCO<u>C</u>H$_3$**). **^{19}F {^{1}H} (500 MHz, D_2O)** δ 230.30

Gal-β(1,3)-6FGlcNAc-N_3 (18)

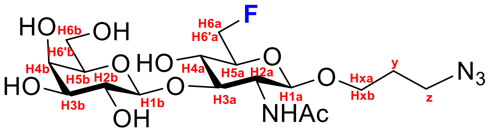

Reactions were assembled by adding 6FGlcNAc-N_3 (**5**, 20 mg), Gal (**9**, 30.42 mg) and ATP (107 mg) in 4 ml of H_2O, to 1300 µL of 1 M Tris buffer (pH 6.5) and 260 µl of 1 M $MgCl_2$ in a 50 mL falcon tube. After adjusting the pH to 6.5, 5 mg of BiGalK and 12 mg of BiGalHexNAcP (Prozomix) were added and the reaction was made up to 13 mL in H_2O. The reaction was incubated at 37 °C with shaking (120 rpm). After 24 h, the enzymes were removed by ultrafiltration (Vivaspin Protein Concentrator Spin Column 30000 MWCO). The reaction was lyophilised and resuspended in dH_2O, dried onto silica gel and purified by flash chromatography (EtOAc: MeOH: H_2O = 4:1:0.1). Fractions containing the desired product (**18**) were pooled and evaporated *in vacuo*. The compound was further purified by BioGel P2 column chromatography in H_2O. Fractions containing the pure desired product (**18**) were pooled and lyophilised (12.1 mg, yield 39 %). Appearance: white powder. **ATR-FTIR** V_{max} (thin film/cm^{-1}) 3276, 2097, 1648, 1554, 1373, 1075. **HRMS** (ESI) *m/z* calcd for $C_{17}H_{29}FN_4NaO_{10}$ (M + Na) 491.1760, found 491.1752. **^{1}H NMR (500 MHz, D_2O)** δ 4.81 & 4.72 (2H, **H-6a & H-6'a**), 4.63 (1H, **H-1a**), 4.47 (d, *J* = 7.7 Hz, 1H, **H-1b**), 4.00 (dt, *J* = 10.4, 5.6 Hz, 1H, **H-xa**), 3.94 (dd, *J* = 3.5, 1.0 Hz, 1H, **H-4b**), 3.89 – 3.83 (m, 2H, **H2-a, H-4a**), 3.81 – 3.65 (m, 7H, **Hxb, H-3a, H-3b, H-5a, H-5b, H-6b, H-6b'**), 3.56 (dd, *J* = 9.9, 7.7 Hz, 1H, **H-2b**), 3.45 – 3.37 (m, 2H, **H-z**), 2.07 (s, 3H, **NHCOC<u>H</u>₃**), 1.91 – 1.84 (m, 2H, **H-y**). **^{13}C NMR (125 MHz, D_2O)** δ 174.54 (**NH<u>C</u>OCH₃**) 103.44 (**C-1b**), 100.95 (**C-1a**), 81.93 (d, *J* = 168.4 Hz, **C-6a**), 81.90 & 54.48 (**C-2a, C-4a**), 75.22, 73.88, 73.74, 72.42, 67.57 & 67.51 (**C-3a, C-3b, C-5a, C-5b**), 70.60 (**C-2b**), 68.47 (**C-4b**), 67.27 (**C-x**), 60.97 (**C-6b**), ,47.70 (**C-z**), 28.05 (**C-x**), 22.15 (**NHCO<u>C</u>H₃**). **^{19}F NMR (471 MHz, D_2O)** δ -235.21.

Gal6F-β(1,3)-6FGlcNAc-N_3 (19)

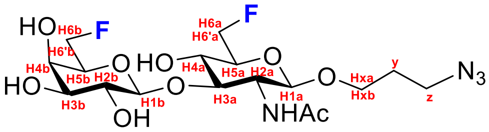

Reactions were assembled by adding 6FGlcNAc-N_3 (**5**, 20 mg), Gal6F (**11**, 30.4 mg) and ATP (107 mg) in 4 mL of H_2O, to 1300 µL of 1 M Tris buffer (pH 6.5) and 260 µL of 1 M $MgCl_2$ in a 50 mL falcon tube. After adjusting the pH to 6.5, 5 mg of BiGalK and 12 mg of BiGalHexNAcP (Prozomix) were added and the reaction was made up to 13 mL in H_2O. The reaction was incubated at 37 °C with shaking (120 rpm). After 24 h, the enzymes were removed by ultrafiltration (Vivaspin Protein Concentrator Spin Column 30000 MWCO). The reaction was lyophilised and resuspended in dH_2O, dried onto silica gel and purified by flash chromatography (EtOAc: MeOH: H_2O = 4:1:0.1). Fractions containing the desired product (**19**) were pooled and evaporated *in vacuo*. The compound was further purified by BioGel P2 column chromatography in H_2O. Fractions containing the pure product (**19**) were pooled and lyophilised (7.8 mg, yield 25 %). Appearance: white powder. **ATR-FTIR** V_{max} (thin film/cm^{-1}) 3282, 2098, 1553, 1372, 1129, 1082, 560. **HRMS** (ESI) *m/z* calcd for $C_{17}H_{28}F_2N_4NaO_9$ (M + Na) 493.1717 found 493.1726. **^{1}H NMR (500 MHz, D_2O)** δ 4.77 – 4.59 (m, 5H, **H-1a, H-6a, H-6a', H-6b & H-6b'**), 4.49 (d, *J* = 7.8 Hz, 1H, **H-1b**), 4.07 – 3.98 (m, 3H, **H-5a, H-5b, H-xa**), 3.89 – 3.81 (m, 2H, **H-2a, H-4a**), 3.75 – 3.65 (m, 4H, **H-3a, H-3b, H-4b, H-xb**), 3.60 – 3.55 (m, 1H, **H-2b**),

3.41 (td, J = 6.6, 2.6 Hz, 2H, **H-z**), 2.07 (s, 3H, **NHCOC$\underline{H}_3$**), 1.91 – 1.85 (m, 2H, **H-y**). **^{13}C NMR (126 MHz, D_2O)** δ 174.55 (**C=O**), 103.58 (**C-1b**), 100.94 (**C-1a**), 82.92 & 54.32 (**C-2a & C-4a**), 81.88 (d, J = 168.7 Hz) & 83.01 (d, J = 165.5 Hz) (**C-6a & C-6b**), 73.76, 73.62, 73.46, 73.30, 72.24, 70.37, 68.00, 67.94, 67.63, 67.58 (**C-2b, C-3a, C-3b, C-4b, C-5a, C-5b**), 67.27 (**C-x**), 47.71 (**C-z**), 28.04 (**C-y**), 22.14 (**NHCO$\underline{C}H_3$**). **^{19}F NMR (471 MHz, D_2O)** δ -230.29, -235.26.

Gal-β(1,3)-6FGlcNTFA-N_3 (20)

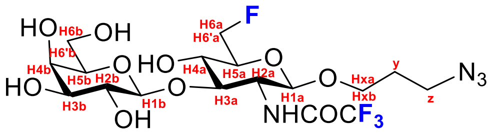

Reactions were assembled by adding 6FGlcNTFA-N_3 (**6**, 30 mg), Gal (**9**, 44.8 mg) and ATP (137.23mg) in 3 mL of ddH_2O, to 1660 µL of 1 M Tris buffer (pH 6.5) and 332 µL of 1 M $MgCl_2$ in a 50 mL falcon tube. After adjusting the pH to 6.5, 8 mg of BiGalK and 25 mg of BiGalHexNAcP was added and the reaction was made up to 16.6 mL with ddH_2O. After incubating the reaction at 37 °C with shaking (180 rpm) for 36 h, the enzymes were removed by ultrafiltration (Vivaspin Protein Concentrator Spin Column 30000 MWCO). The reaction was lyophilised, resuspended in ddH_2O and purified by BioGel P2 column chromatography in H_2O. Fractions containing the desired product (**20**) were pooled and lyophilised (16.6 mg, yield 76 %). Yield calculated based on the recovery of unreacted 6FGlcNTFA-N_3 (**6**, 15 mg) starting material. Appearance: white powder. **ATR-FTIR** V_{max} (thin film/cm^{-1}) 2981, 2106, 1922, 1704, 1431, 1381, 1156, 1081, 952, 741, 692, 507. HRMS (ESI) *m/z* calcd for $C_{17}H_{26}F_4N_4NaO_{10}$ (M + Na) 545.1477, found 545.1480. **^{1}H NMR (500 MHz, D_2O)** δ 4.82-4.73 (2H, **H-6a & H-6'a**), 4.75 – 4.68 (m, 1H, **H-1a**), 4.45 (d, J = 7.7 Hz, 1H, **H-1b**), 4.04 – 3.95 (m, 2H, **H-6b & H-6b'**), 3.96 – 3.92 (m, 2H, **H-2a**, **H-4b**), 3.86 – 3.76 (m, 2H, **H-xa & H-xb**), 3.76 – 3.76 (m, 4H, **H-3a**, **H-4a, H-5a, H-5b**) 3.65 (dd, J = 9.9, 3.4 Hz, 1H, **H-3b**), 3.57 – 3.50 (m, 1H, **H-2b**), 3.39 (td, J = 6.7, 1.8 Hz, 2H, **H-z**), 1.87 (p, J = 6.4 Hz, 2H, **H-y**). **^{13}C NMR (126 MHz, D_2O)** δ 159.32 (d, J = 37.7 Hz, **C=0**), 115.73 (q, J = 286.3 Hz, **NHCO$\underline{C}F_3$**), 103.53 (**C-1b**), 100.46 (**C-1a**), 81.15 & 68.44 (**C-2a, C-4b**), 81.84 (d, J = 168.6 Hz, **C-6a**), 75.27, 74.29, 73.99 &73.84 (**C-3a, C-4a, C-5a, C-5b**), 72.45 (**C-3b**), 70.50 (**C-2b**), 70.42, 67.48 (**C-6b**), 60.93 (**C-x**), 54.92, 47.65 (**C-z**), 28.00 (**C-y**). **^{19}F {^{1}H} (500 MHz, D_2O)** δ -75.62, -235.27.

Gal6F-β(1,3)-6FGlcNTFA-N_3 (21)

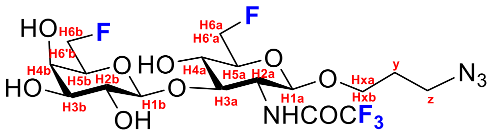

Reactions were assembled by adding 6FGlcNTFA-N_3 (**6**, 14.1 mg), Gal6F (**11**, 25.1 mg) and ATP (70 mg) in 4 ml of dH_2O, to 912 µL of 1 M Tris buffer (pH 6.5) and 184 µL of 1 M $MgCl_2$ in a 50 mL falcon tube. After adjusting the pH to 6.5, 10 mg of BiGalK and 9.6 mg of BiGalHexNAcP were added and the reaction was made up to 9.21 mL in dH_2O. The reaction was incubated at 37 °C with shaking (120 rpm). After 18 h, another 24 mg of BiGalHexNAcP was added and the reaction was further incubated at 37 °C. After another 24 h, a further 14 mg of BiGalHexNAcP was added and the reaction was incubated at 37 °C for a further 72 h. The enzymes were removed by ultrafiltration (Vivaspin

Protein Concentrator Spin Column 30000 MWCO). The reaction was lyophilised and resuspended in dH_2O, dried onto silica gel and purified by flash chromatography (EtOAc: MeOH: H_2O = 4:1:0.1). Fractions containing the desired product (**21**) were pooled and evaporated *in vacuo*. The compound was further purified by BioGel P2 column chromatography in H_2O. Fractions containing the pure desired product (**21**) were pooled and lyophilised (4.6 mg, yield 36 %). Yield calculated based on the recovery of unreacted 6FGlcNTFA-N_3 (**6**, 5.3 mg) starting material. Appearance: white powder. **ATR-FTIR** V_{max} (thin film/cm^{-1}) 3256, 2105, 1705, 1570, 1370, 1131, 1081. **HRMS** (ESI) *m/z* calcd for $C_{17}H_{25}F5N_4NaO_9$ (M + Na) 547.1440, found 547.1434. **^{1}H NMR (500 MHz, D_2O)** δ 4.77 – 4.59 (m, 5H, **H-1a, H-6a, H-6a', H-6b & H-6b'**), 4.49 (d, *J* = 7.8 Hz, 1H, **H-1b**), 4.07 – 4.03 (m, 1H, **H-5b**), 4.02 – 3.96 (m, 3H, **H-4a**, **H-xa, H-xb**), 3.90 (dd, *J* = 10.4, 8.0 Hz, 1H, **H-2a**), 3.75 – 3.66 (m, 4H, **H-5a**, **H-3a**, **H-3b**, **H-4b**), 3.59 – 3.54 (m, 1H, **H-2b**), 3.40 (td, *J* = 6.7, 1.9 Hz, 2H, **H-z**), 1.87 (p, *J* = 6.4 Hz, 2H, **H-y**). **^{13}C NMR (126 MHz, D_2O)** δ 182.36 (**C=0**), 103.56 (**C-1b**), 100.61 (**C-1a**), 82.99 (d, *J* = 165.4 Hz, **C-6b**), 82.70 (**C-2a**), 81.83 (d, *J* = 168.6 Hz, **C-6a**), 73.88, 73.73, 73.48, 73.32 & 72.24, 67.98, 67.92, 67.54, 67.48 (**C-3a, C-3b, C-4a, C-4b, C-5a, C-5b**, 70.34 (**C-2b**), 67.43 (**C-x**), 47.65 (**C-z**), 28.01 (**C-y**). **^{19}F {^{1}H} (500 MHz, D_2O)** δ -75.35, -230.32, -235.28.

Gal-β(1,3)-6,6diFGlcNAc-N_3 (22)

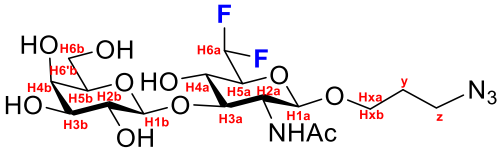

Reactions were assembled by adding 6,6diFGlcNAc-N_3 (**8**, 18.7 mg), Gal (**9**, 68 mg) and ATP (175 mg) in 4 mL of H_2O, to 1150 µL of 1M Tris buffer (pH 6.5) and 230 µL of 1M $MgCl_2$ in a 50 mL falcon tube. After adjusting the pH to 6.5, 16.5 mg of BiGalK and 14.4 mg of BiGalHexNAcP (Prozomix) were added and the reaction was made up to 11.5 mL in MilliQ H_2O. The reaction was incubated at 37 °C with shaking (120 rpm). After 24 h, the enzymes were removed by ultrafiltration (Vivaspin Protein Concentrator Spin Column 30000 MWCO). The reaction was lyophilised and resuspended in dH_2O, dried onto silica gel and purified by flash chromatography (EtOAc: MeOH: H_2O = 4:1:0.1). Fractions containing the desired product (**22**) were pooled and evaporated *in vacuo*. The compound was further purified by BioGel P2 column chromatography in H_2O. Fractions containing the pure product (**22**) were pooled and lyophilised (15.2 mg, yield 54 %). Appearance: white powder. **ATR-FTIR** V_{max} (thin film/cm^{-1}) 3270, 2118, 2012, 1618, 1572, 1131, 1063, 1034, 951, 892, 774, 611, 561, 466. **HRMS** (ESI) *m/z* calcd for $C_{17}H_{28}F_2N_4NaO_{10}$ (M + Na) 509.1666, found 509.1670. **^{1}H NMR (500 MHz, D_2O)** δ 6.23 (t, *J* = 53.4 Hz, 1H, **H-6a**), 4.69 – 4.66 (m, 1H, **H-1a**), 4.47 (d, *J* = 7.7 Hz, 1H, **H-1b**), 4.01 (dt, *J* = 10.9, 5.6 Hz, 1H, **H-xa**), 3.94 (dd, *J* = 3.4, 0.9 Hz, 1H, **H-4b**), 3.90 – 3.71 (m, 8H, H-xb, H-6b, **H-6b', H-2a, H-3a, -H4a, H-5a, H-5b**), 3.67 (dd, *J* = 9.9, 3.4 Hz, 1H, **H-3b**), 3.55 (dd, *J* = 9.9, 7.7 Hz, 1H, **H-2b**), 3.41 (td, *J* = 6.6, 2.7 Hz, 2H, **H-z**), 2.07 (s, 3H, **NHCOC$\underline{H}_3$**), 1.92 – 1.85 (m, 2H, **H-y**). **^{13}C NMR (125 MHz, D_2O)** δ 174.58 (**C=O**), 113.41 (**C-6a**), 103.42 (**C-1b**), 101.16 (**C-1a**), 81.46, 75.24, 67.92, 54.24 (**C-2a, C-3a, C-4a, C-5b**), 72.77 (t, *J* = 20.2 Hz, **C-5a**), 72.41 (**C-3b**), 70.58 (**C-2b**), 68.46 (**C-4b),** 67.48 (**H-x**), 60.98 (**C-6b**), 47.68 (**H-z**), 28.02 (**C-y**), 22.14 (**NHCO$\underline{C}H_3$**). **^{19}F {^{1}H} (500 MHz, D_2O)** δ -132.12, -132.73, -134.04, -134.64.

Gal-β(1,3)-6,6diFGlcNTFA-N$_3$ (23)

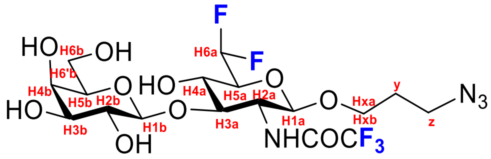

Reactions were assembled by adding 6,6diFGlcNTFA-N$_3$ (**7**, 21 mg), Gal (**9**, 60.6 mg) and ATP (169 mg) in 4 mL of H_2O, to 1100 µL of 1 M Tris buffer (pH 6.5) and 222 µL of 1 M $MgCl_2$ in a 50 mL falcon tube. After adjusting the pH to 6.5, 16.5 mg of BiGalK and 14.4 mg of BiGalHexNAcP (Prozomix) were added and the reaction was made up to 11 mL in MilliQ H_2O. The reaction was incubated at 37 °C with shaking (120 rpm). After 24 h, the enzymes were removed by ultrafiltration (Vivaspin Protein Concentrator Spin Column 30000 MWCO). The reaction was lyophilised and resuspended in dH_2O, dried onto silica gel and purified by flash chromatography (EtOAc: MeOH: H_2O = 4:1:0.1). Fractions containing the desired product (**23**) were pooled and evaporated *in vacuo*. The compound was further purified by BioGel P2 column chromatography in H_2O. Fractions containing the pure product (**23**) were pooled and lyophilised (9 mg, yield 31 %). Yield calculated based on the recovery of unreacted 6,6diFGlcNTFA-N$_3$ (**7**, 1 mg) starting material. Appearance: white powder. **ATR-FTIR** V_{max} (thin film/cm^{-1}) 3251, 2109, 1709, 1575, 1218, 1161, 1041, 888, 770, 529. **HRMS** (ESI) *m/z* calcd for $C_{17}H_{25}F_5N_4NaO_{10}$ (M + Na) 563.1366 found 563.1383. **^{1}H NMR (500 MHz, D_2O)** δ 6.33 – 6.09 (m, 1H, **H-6a**), 4.73 (d, *J* = 8.2 Hz, 1H, **H-1a**), 4.42 (d, *J* = 7.7 Hz, 1H, **H-1b**), 4.03 – 3.89 (m, 4H, **H-xa, H-2a, H-4b, H-5b**), 3.89 – 3.81 (m, 1H, **H-5a**), 3.81 – 3.74 (m, 3H, **H-6b, H-6b', H-4a**), 3.74 – 3.69 (m, 2H, **H-xb, H-3a**), 3.62 (dd, *J* = 9.9, 3.4 Hz, 1H, **H-3b**), 3.51 (dd, *J* = 10.0, 7.8 Hz, 1H, **H-2b**), 3.36 (td, *J* = 6.6, 1.7 Hz, 2H, **H-z**), 1.88 – 1.82 (m, 2H, **H-y**). **^{13}C NMR (125 MHz, D_2O)** δ 159.34 (d, *J* = 37.7 Hz, **C=O**), 113.28 (t, *J* = 242.9 Hz, **C-6a**), 103.50 (**C-1b**), 100.50 (**C-1a**), 81.10, 68.41 & 54.69 (**C-2a, C-4b, C-5b**), 75.25 (**C-3a**), 72.72 (d, *J* = 20.3 Hz, **C-5a**) 72.39 (**C-3b**), 70.45 (**C-2b**), 67.82 (d, *J* = 5.0 Hz, **C-4a**), 67.66 (**C-x**), 60.92 (**C-6b**), 47.59 (**C-z**), 27.94 (**C-y**). **^{19}F {^{1}H} (500 MHz, D_2O)** δ -75.64, -132.23, -132.83, -134.15, -134.76.

NMR Spectra

3-Bromopropyl 2-acetamido-2-deoxy-3,4,6-tri-*O*-acetyl-*β*-D-glucopyranoside S2

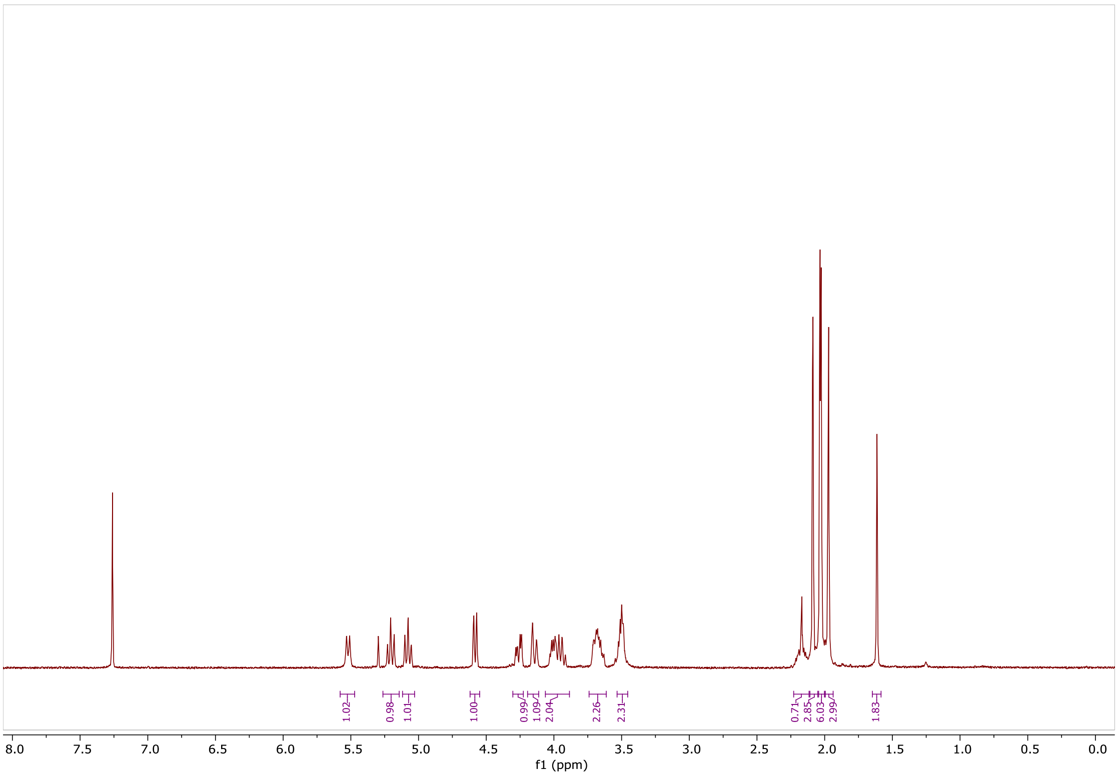

3-Azidopropyl 2-acetamido-2-deoxy-3,4,6-tri-O-acetyl-β-D-glucopyranoside S3

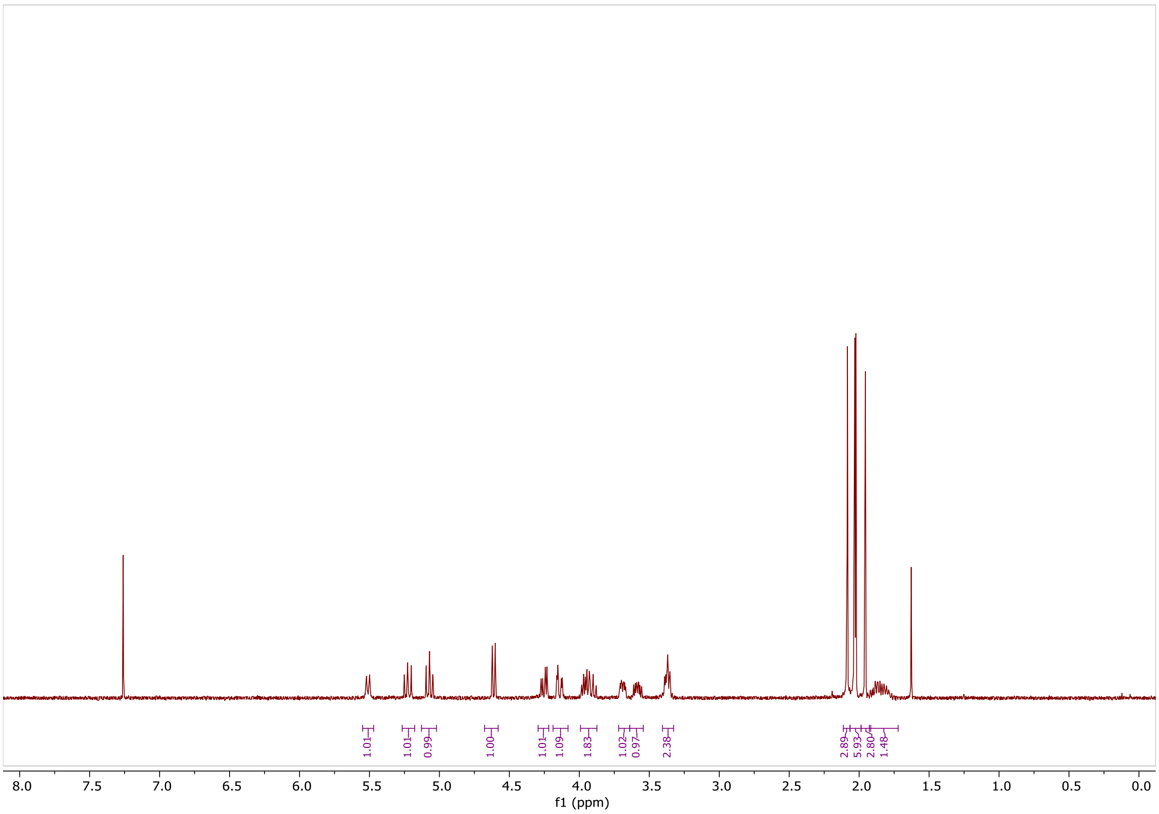

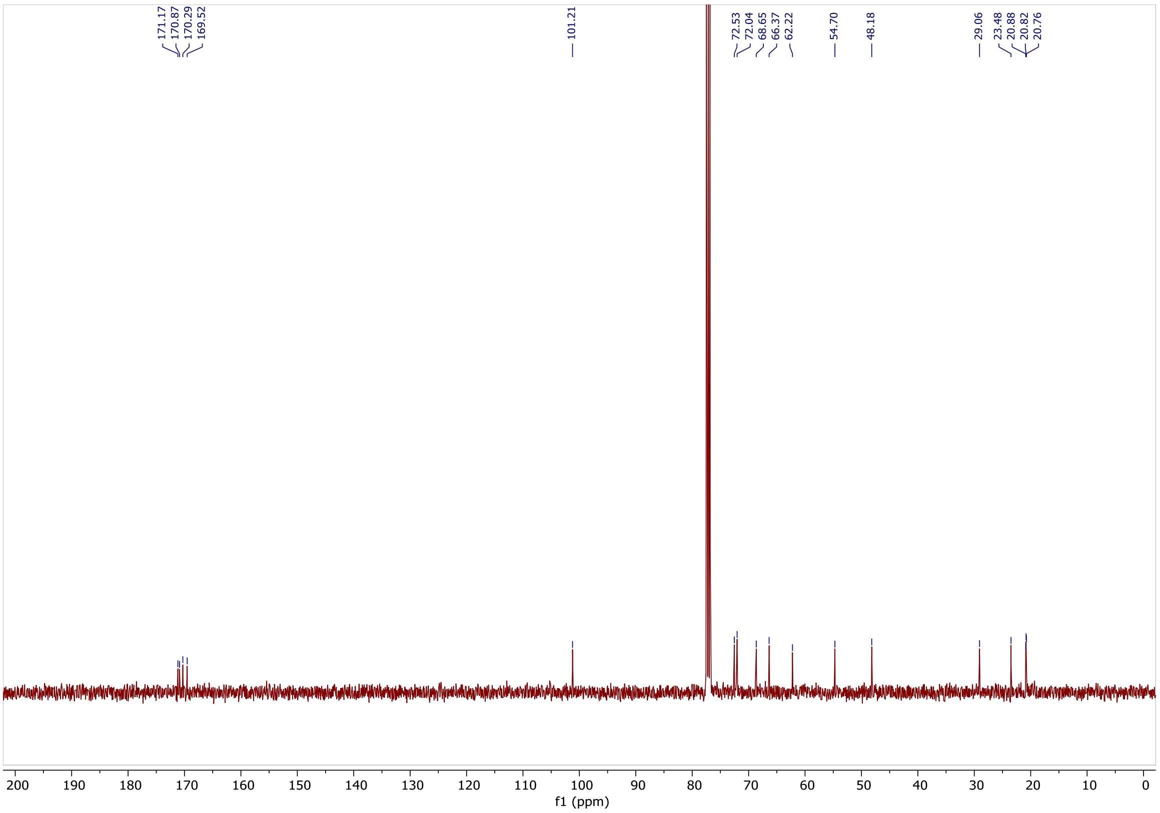

3-Azidopropyl 2-acetamido-2-deoxy-*β*-D-glucopyranoside 14

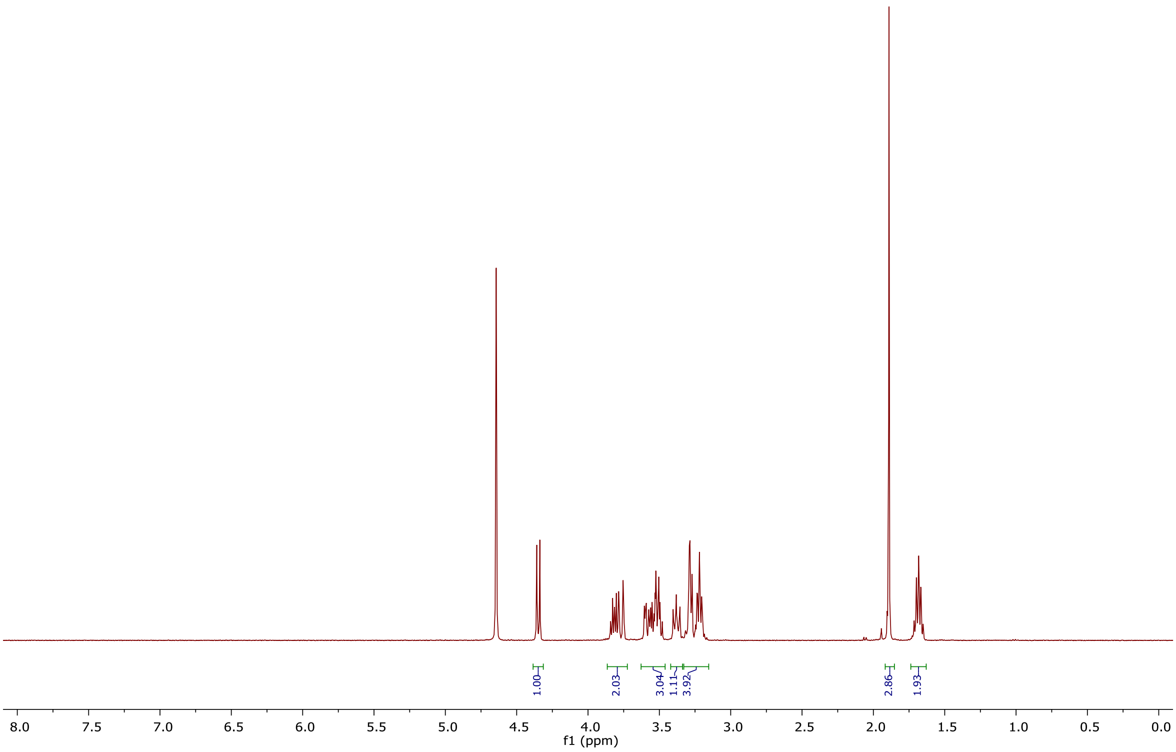

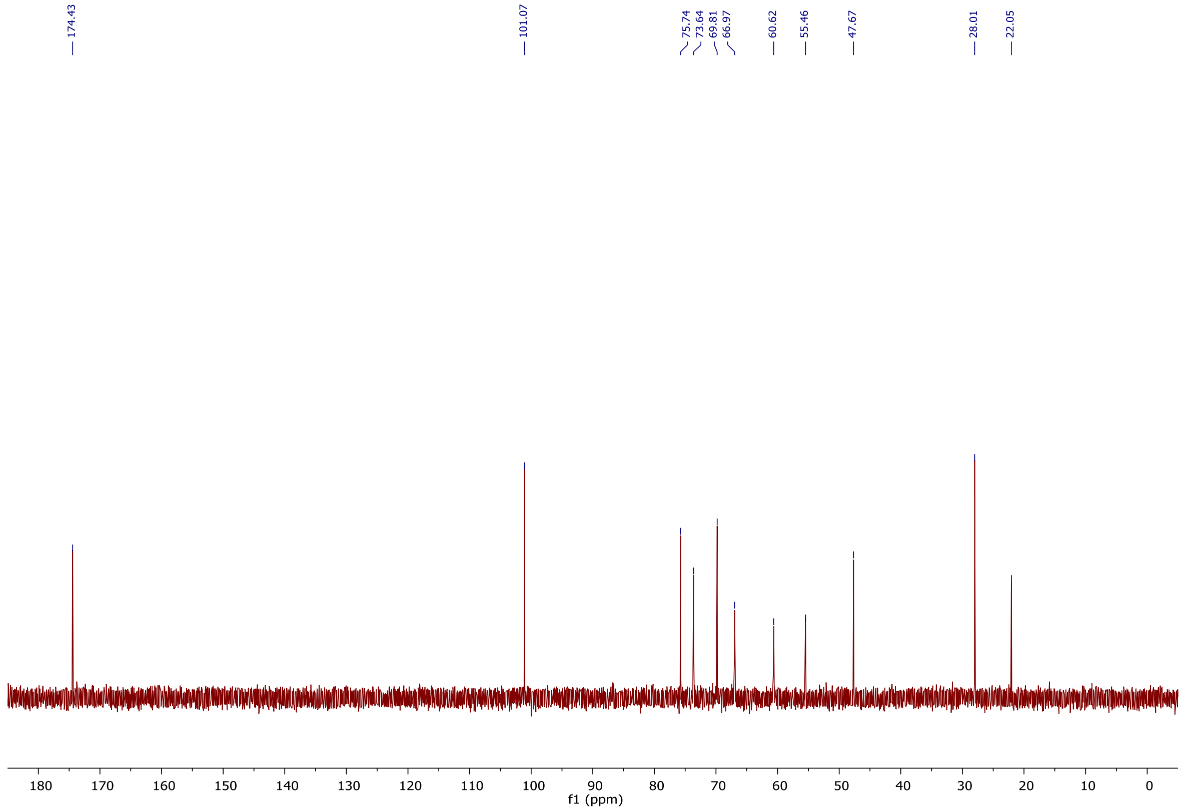

3-Chloropropyl 3,4,6-tri-*O*-acetyl-2-deoxy-2-azido-D-glucopyranoside (BLS1)

^{1}H NMR (500 MHz, CDCl₃)

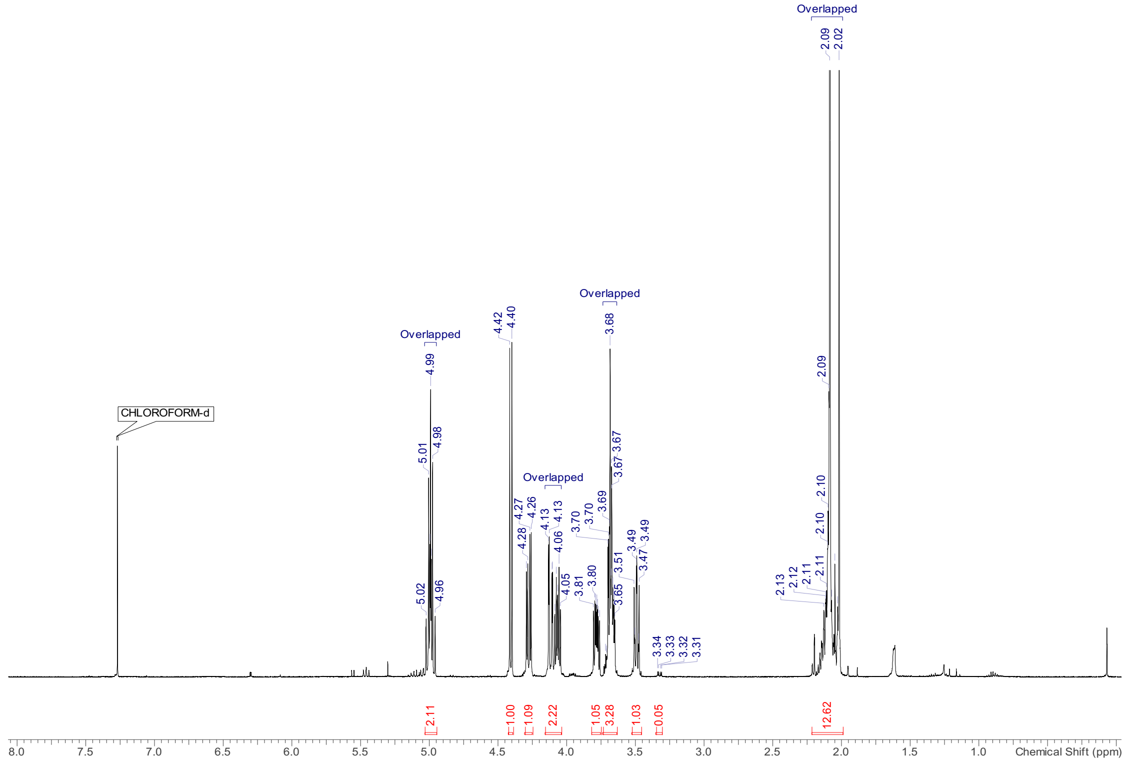

$^{13}C\{^1H\}$ NMR (126 MHz, $CDCl_3$)

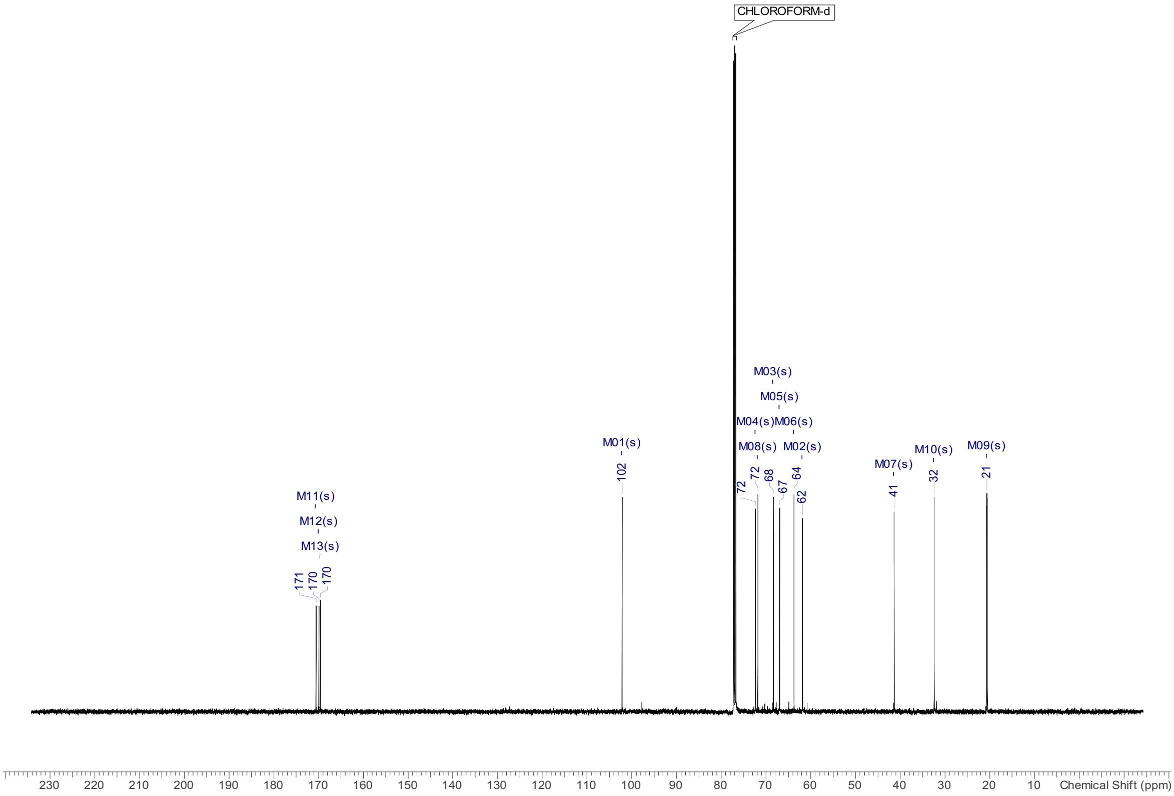

3-Chloropropyl 2-deoxy-2-azido-D-glucopyranoside (BLS2)

^1H NMR (400 MHz, CD_3OD)

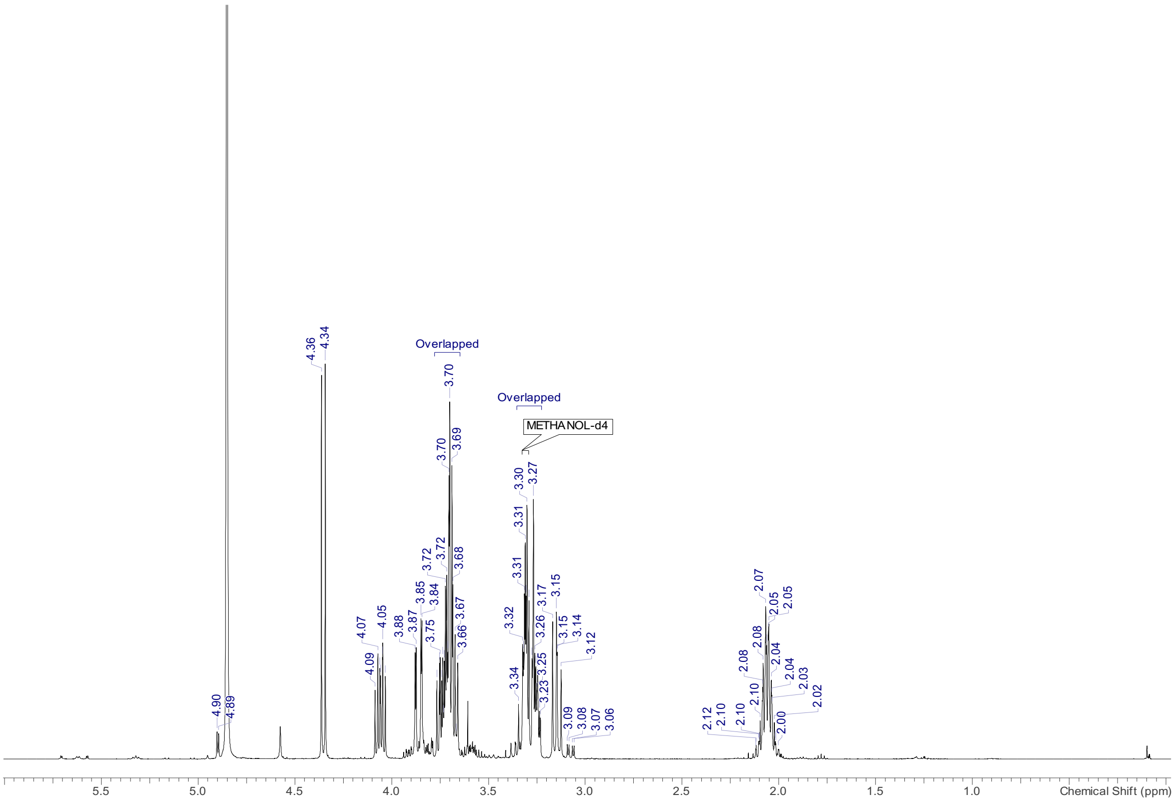

$^{13}C\{^1H\}$ NMR (101 MHz, CD_3OD)

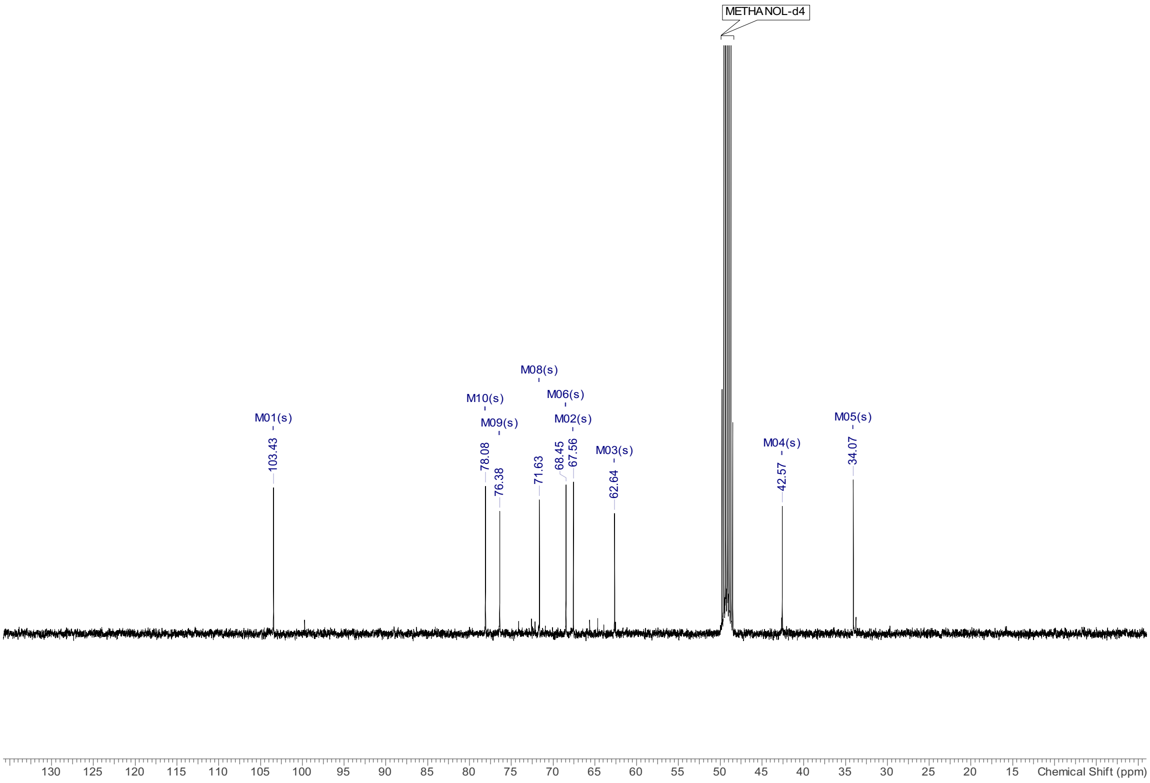

3-Chloropropyl 2-deoxy-2-azido-3,4-O-[(2’S,3’S)-2',3'-dimethoxybutane-2',3'-diyl]-d-glucopyranoside (BLS3)

^1H NMR (500 MHz, $CDCl_3$)

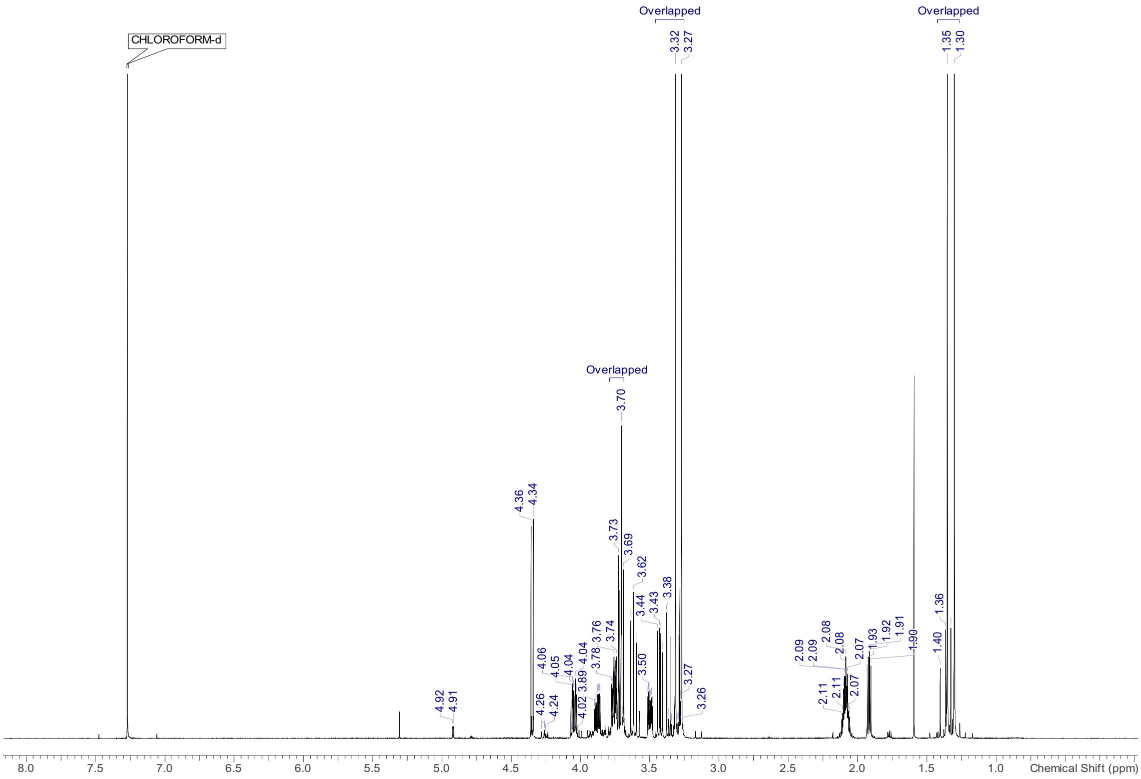

$^{13}C\{^1H\}$ NMR (126 MHz, $CDCl_3$)

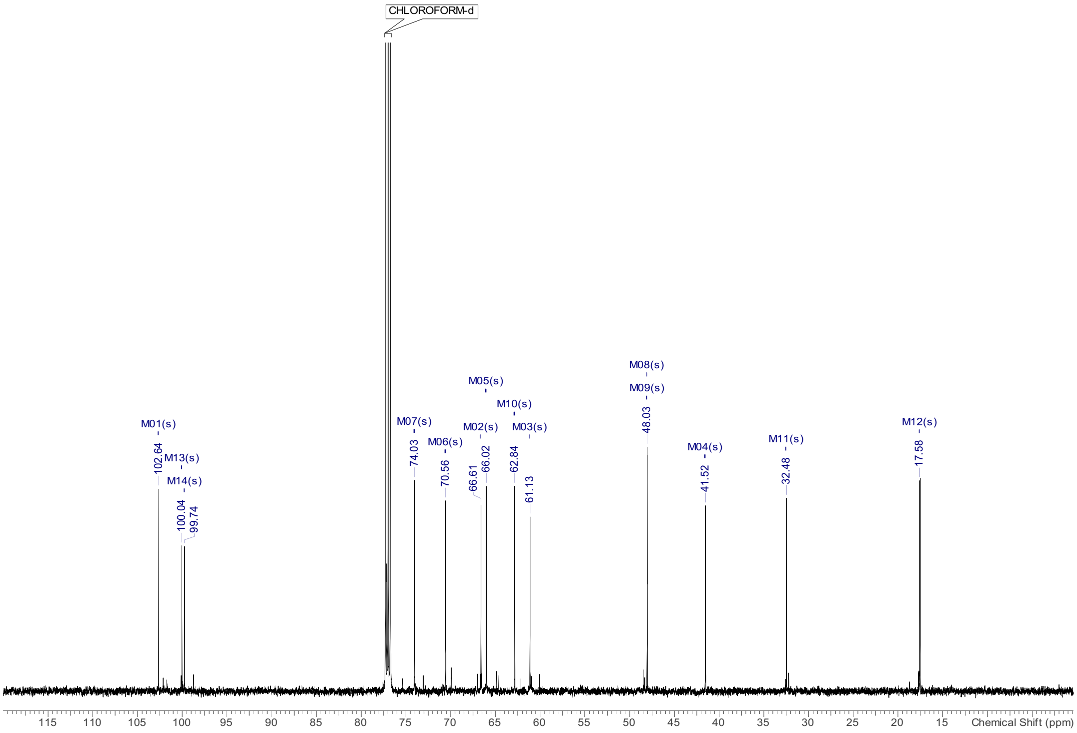

Synthesis of 3-Chloropropyl 2,6-dideoxy-2-azido-6-fluoro-3,4-*O*-[(2'*S*,3'*S*)-2',3'-dimethoxybutane-2',3'-diyl]-D-glucopyranoside (BLS4)

^1H NMR (400 MHz, $CDCl_3$)

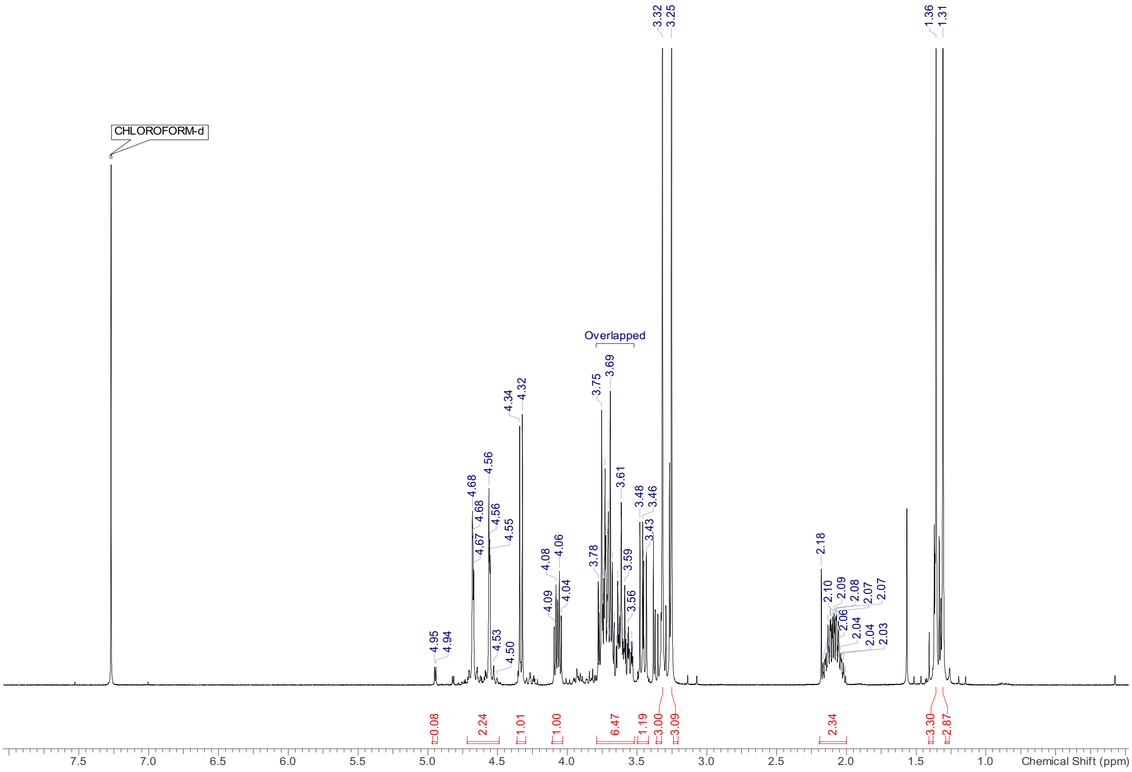

$^{13}C\{^1H\}$ NMR (101 MHz, $CDCl_3$)

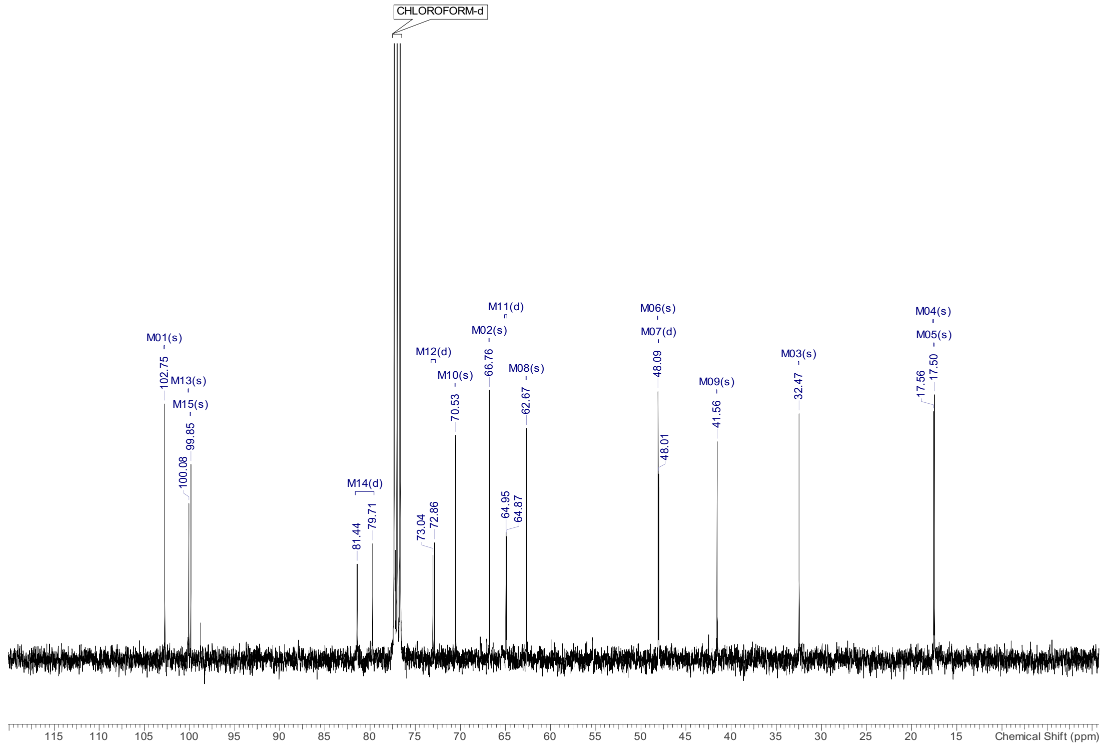

^{19}F NMR (376 MHz, $CDCl_3$)

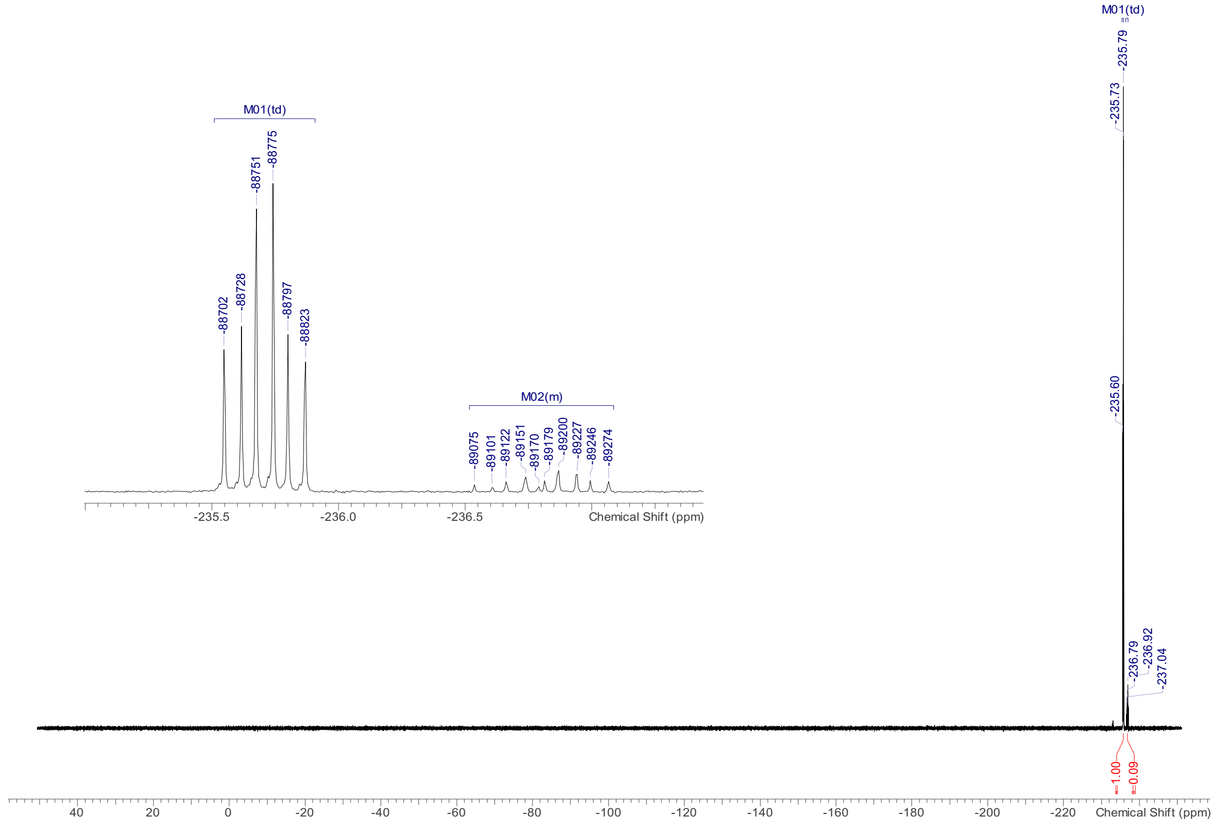

$^{19}F\{^{1}H\}$ NMR (376 MHz, $CDCl_3$)

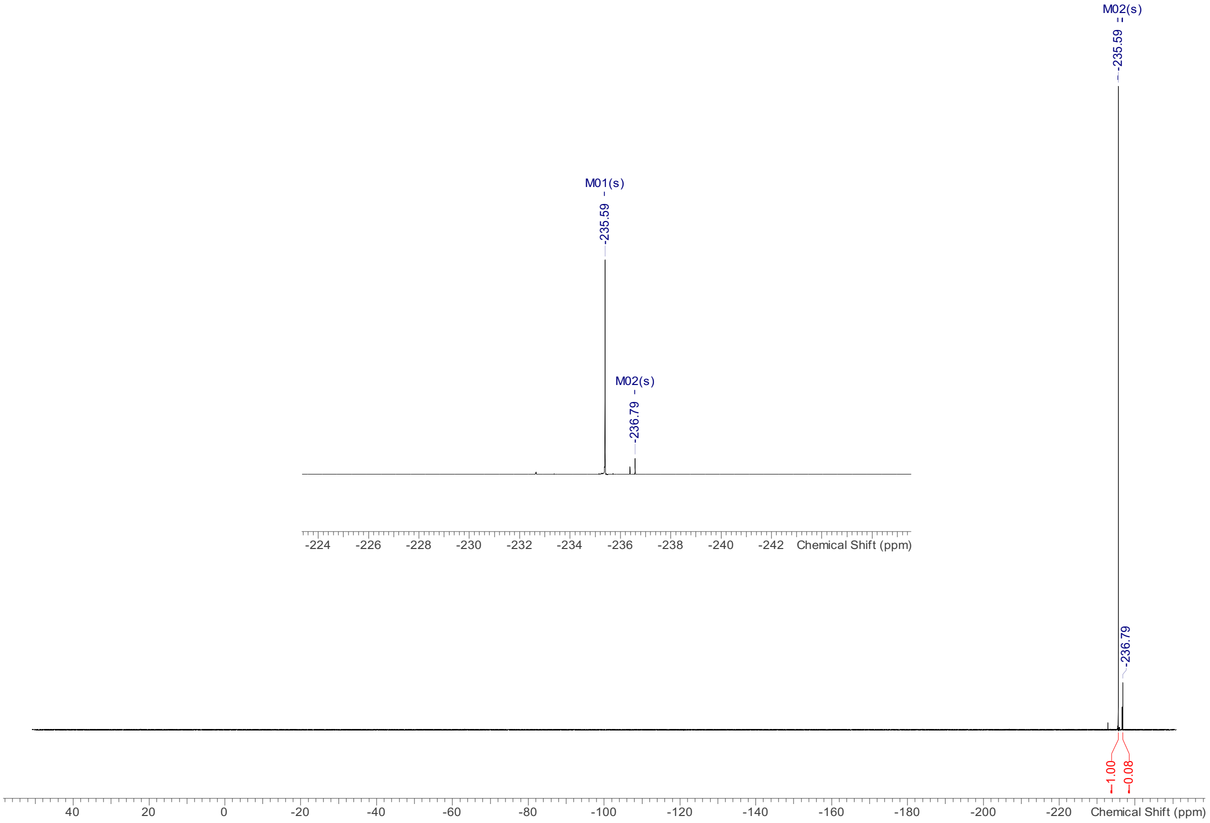

3-Azidopropyl-2,6-dideoxy-2-acetimido-6-fluoro-β-D-glucopyranoside (B, 5)

^{1}H NMR (500 MHz, CD_3OD)

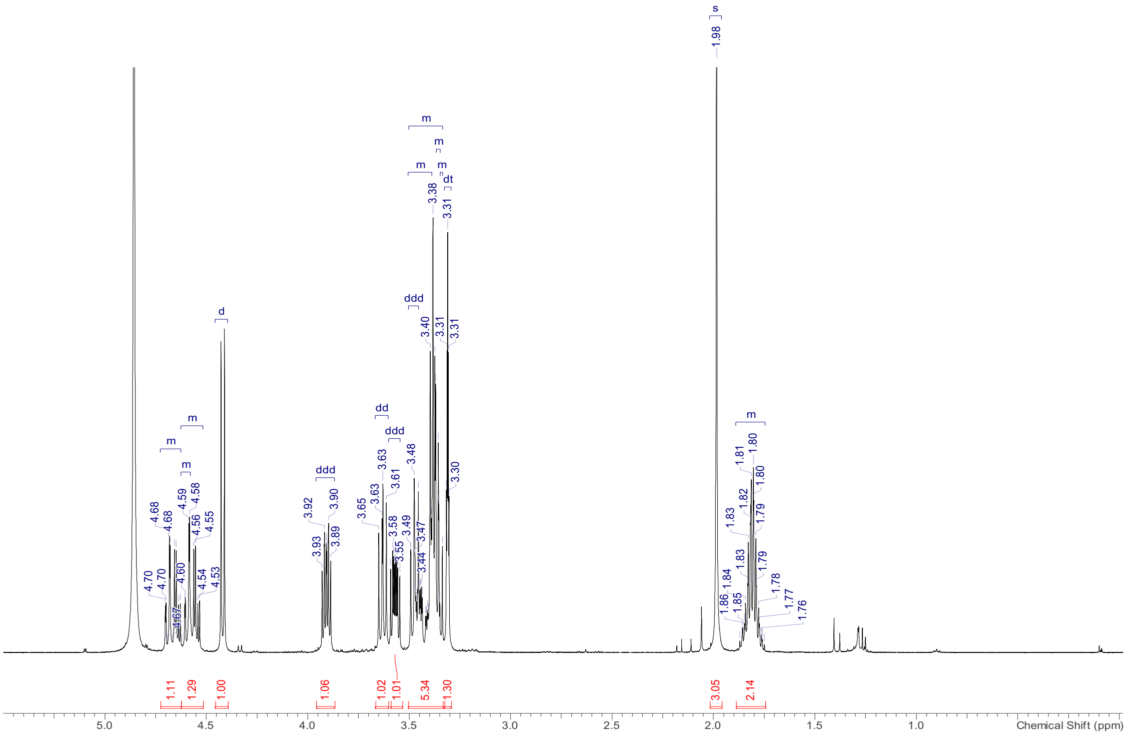

$^{1}H\{^{19}F\}$ NMR (500 MHz, CD_3OD)

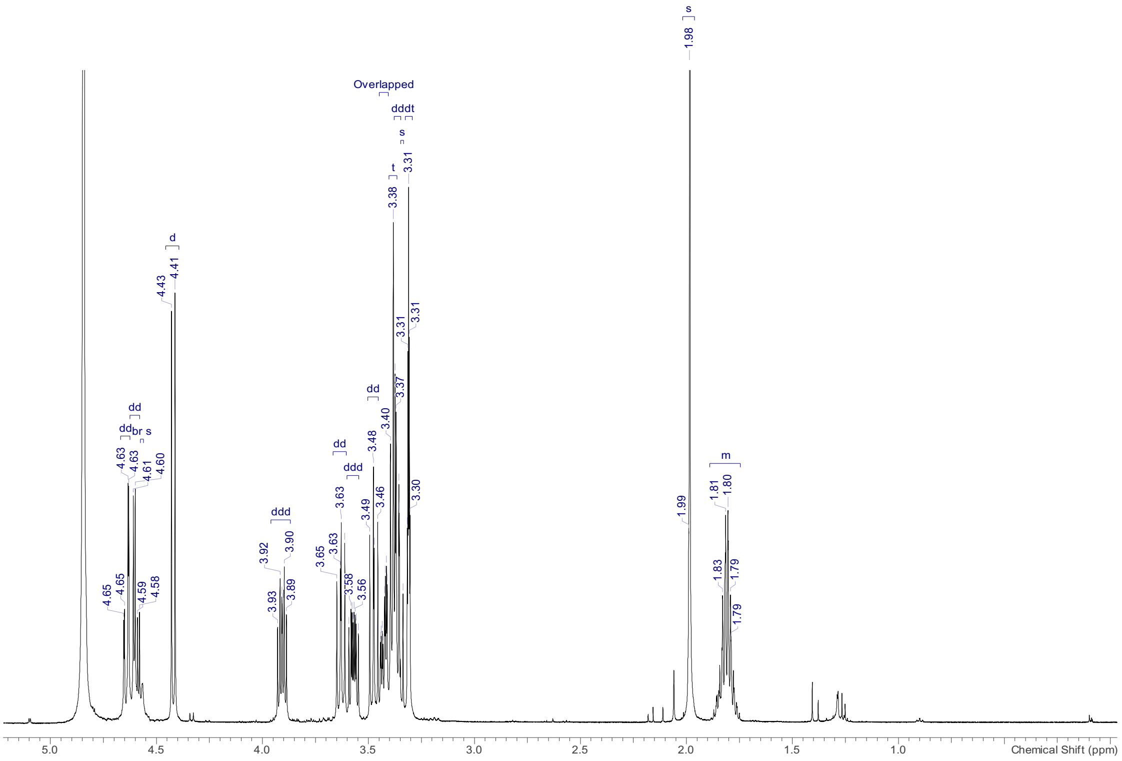

$^{13}C\{^{1}H\}$ NMR (500 MHz, CD_3OD)

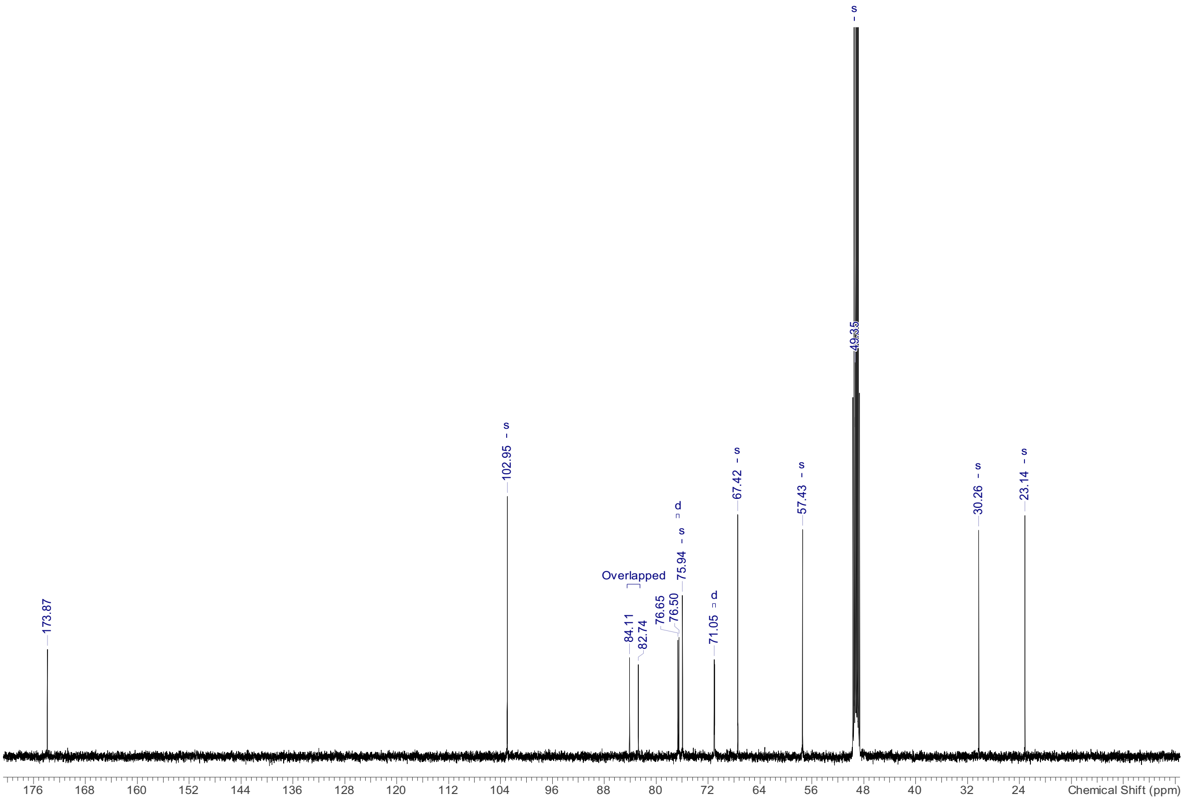

^{19}F NMR (470 MHz, CD_3OD)

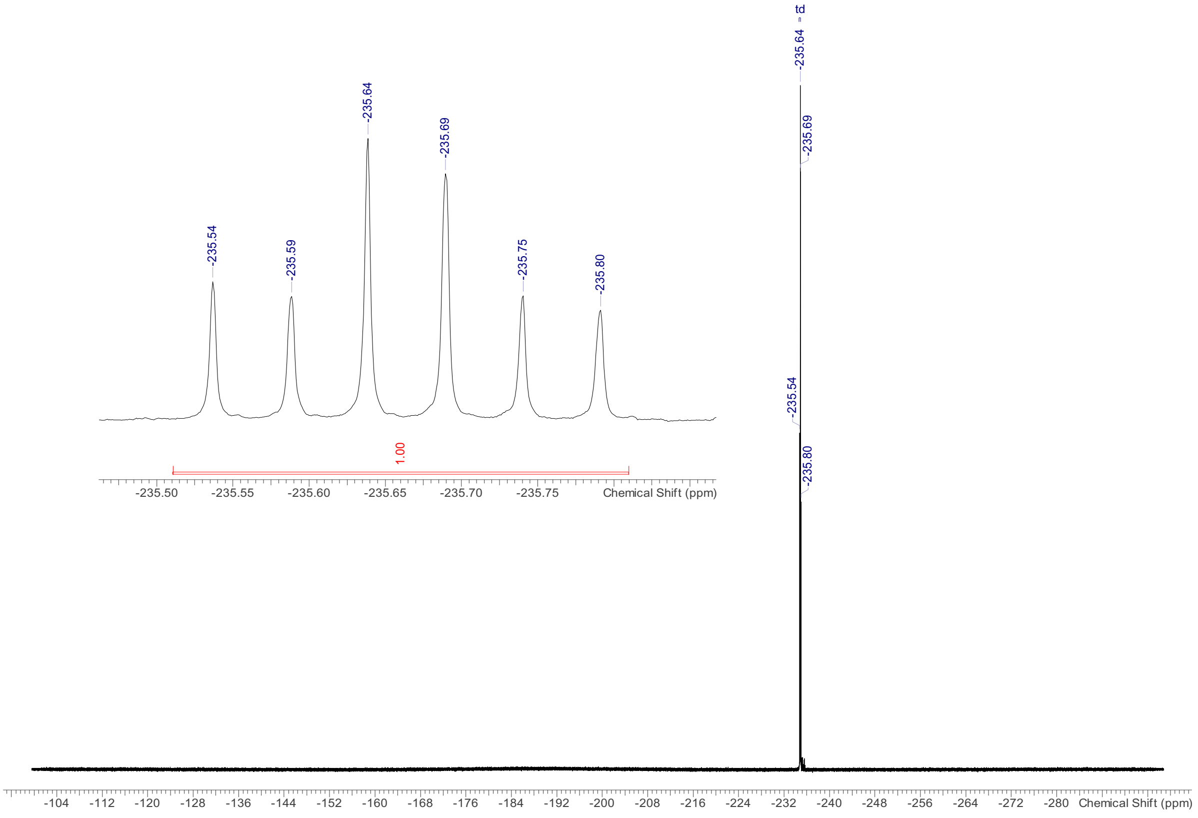

$^{19}F\{^1H\}$ NMR (470 MHz, CD_3OD)

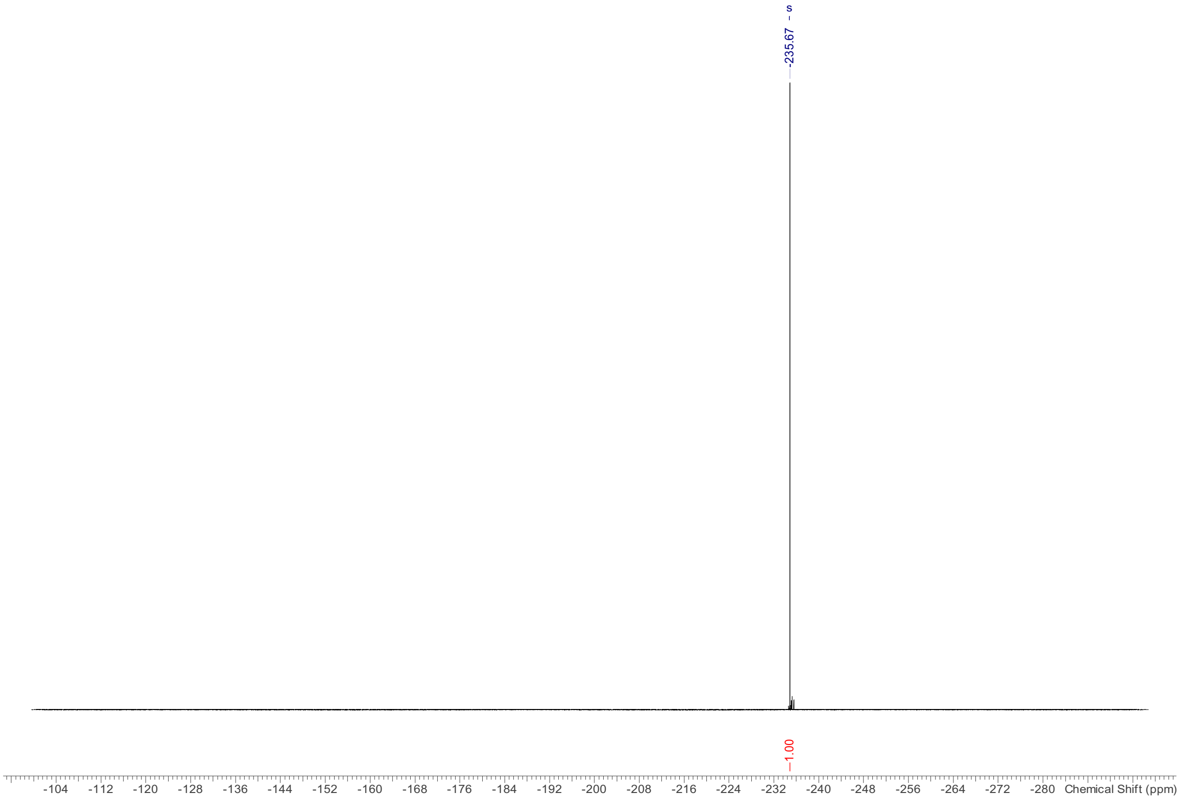

3-Azidopropyl 2,6-dideoxy-2-trifluoroacetimido-6-fluoro-β-D-glucopyranoside (C, 6)

^{1}H NMR (500 MHz, CD_3OD)

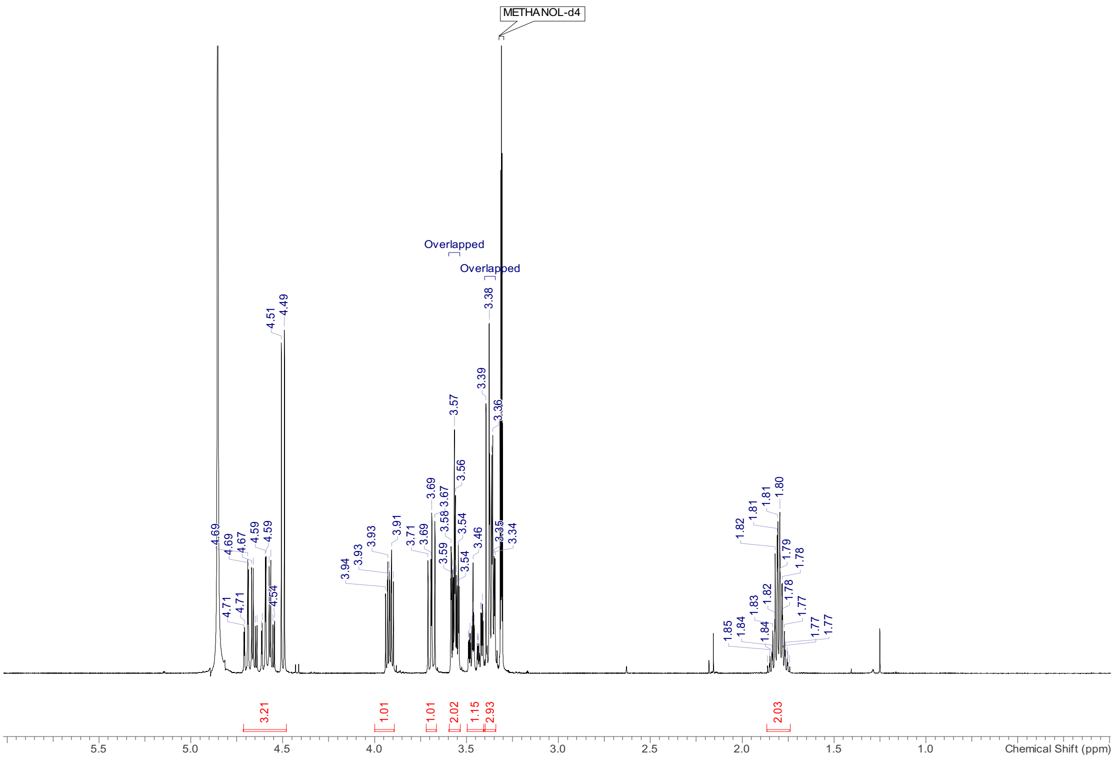

$^{13}C\{^{1}H\}$ NMR (126 MHz, CD_3OD)

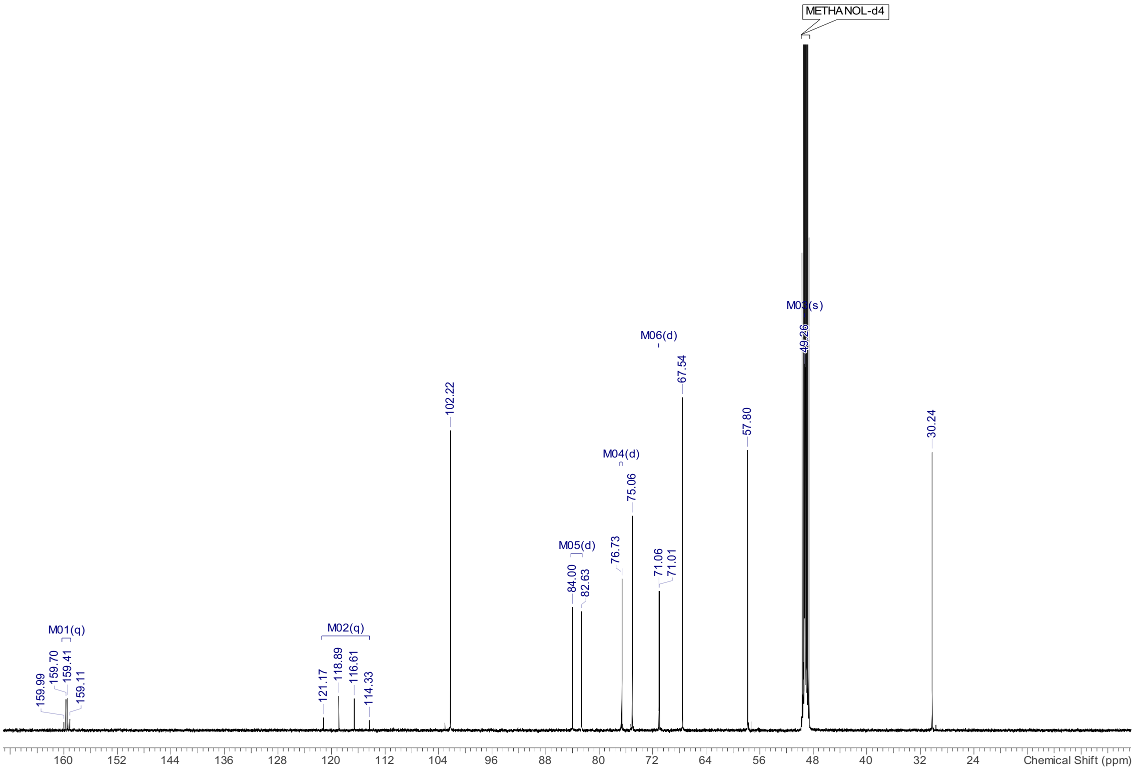

^{19}F NMR (470 MHz, CD_3OD)

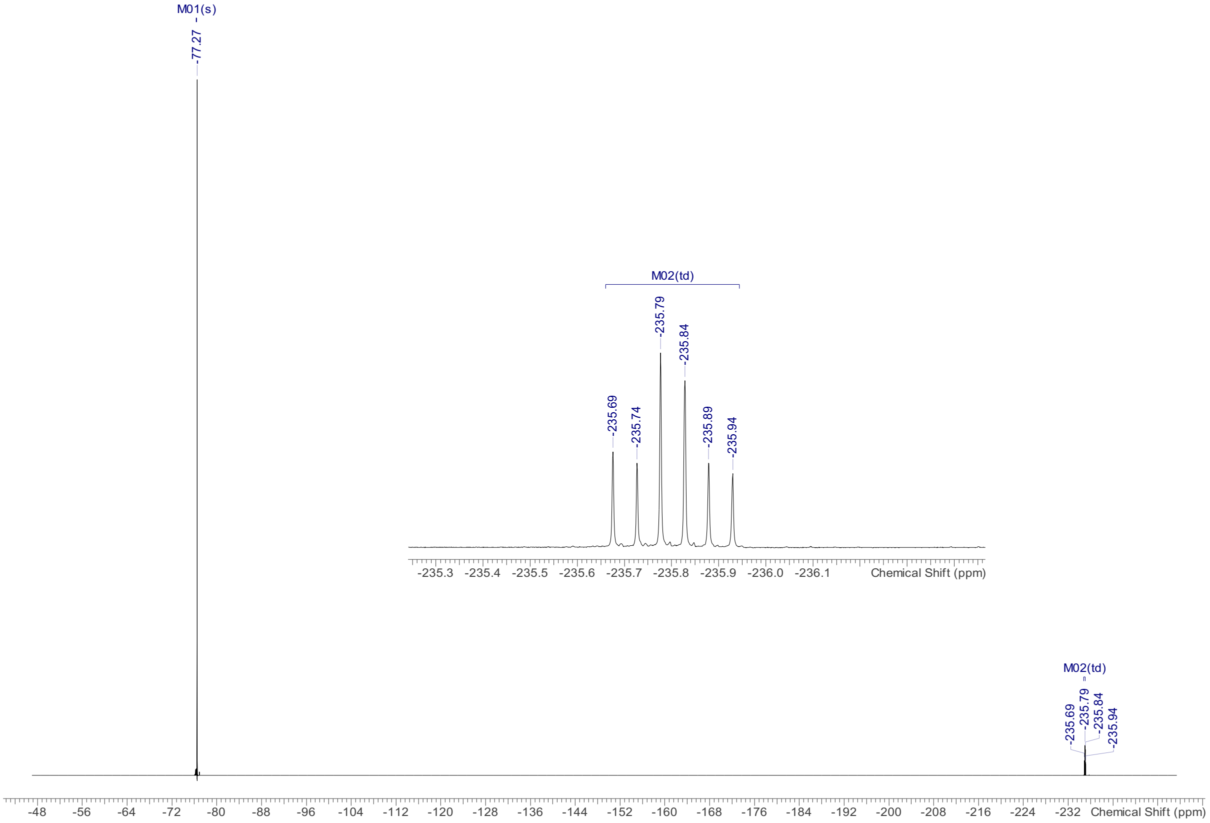

^{19}F{^{1}H} NMR (470 MHz, CD_3OD)

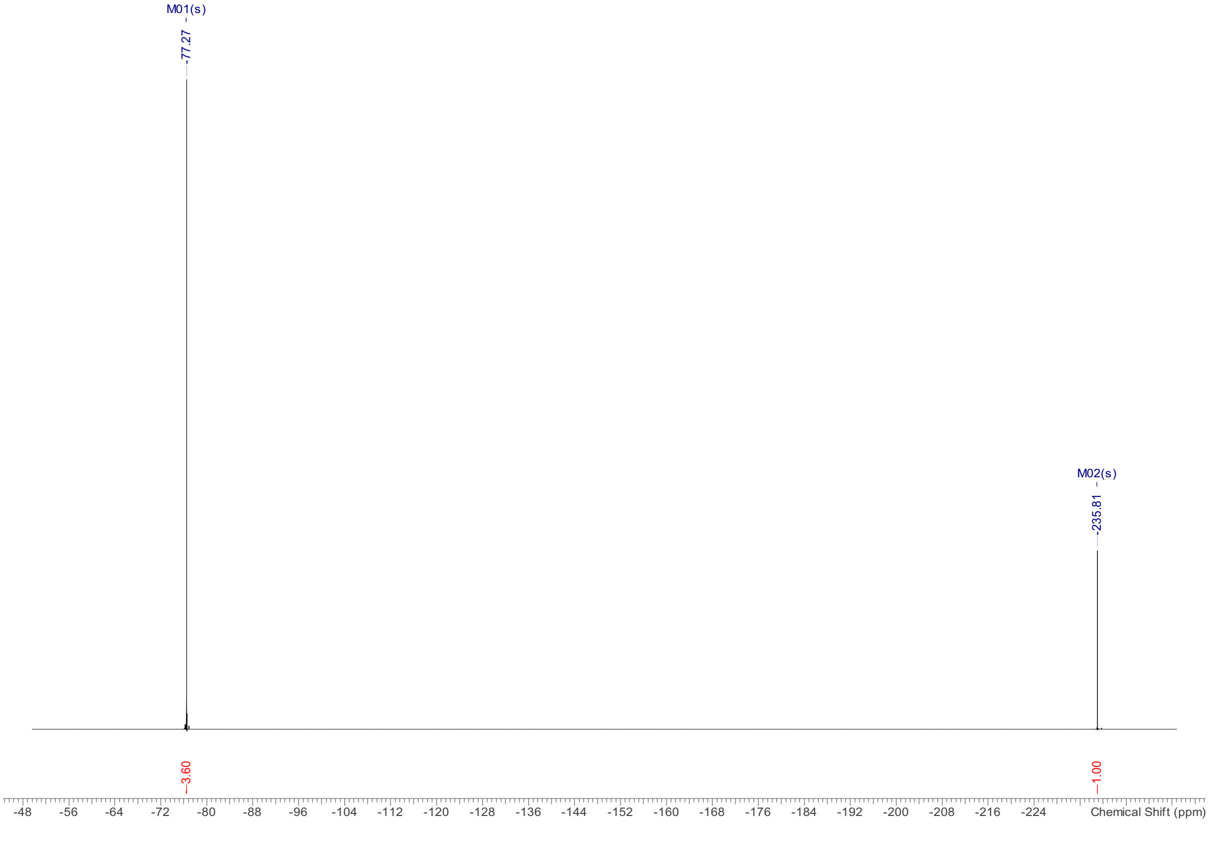

3-Chloropropyl 2,6-dideoxy-2-azido-3,4-*O*-[(2'*S*,3'*S*)-2',3'-dimethoxybutane-2',3'-diyl]-6,6-difluoro-D-glucopyranoside (BLS11)

^{1}H NMR (400 MHz, $CDCl_3$)

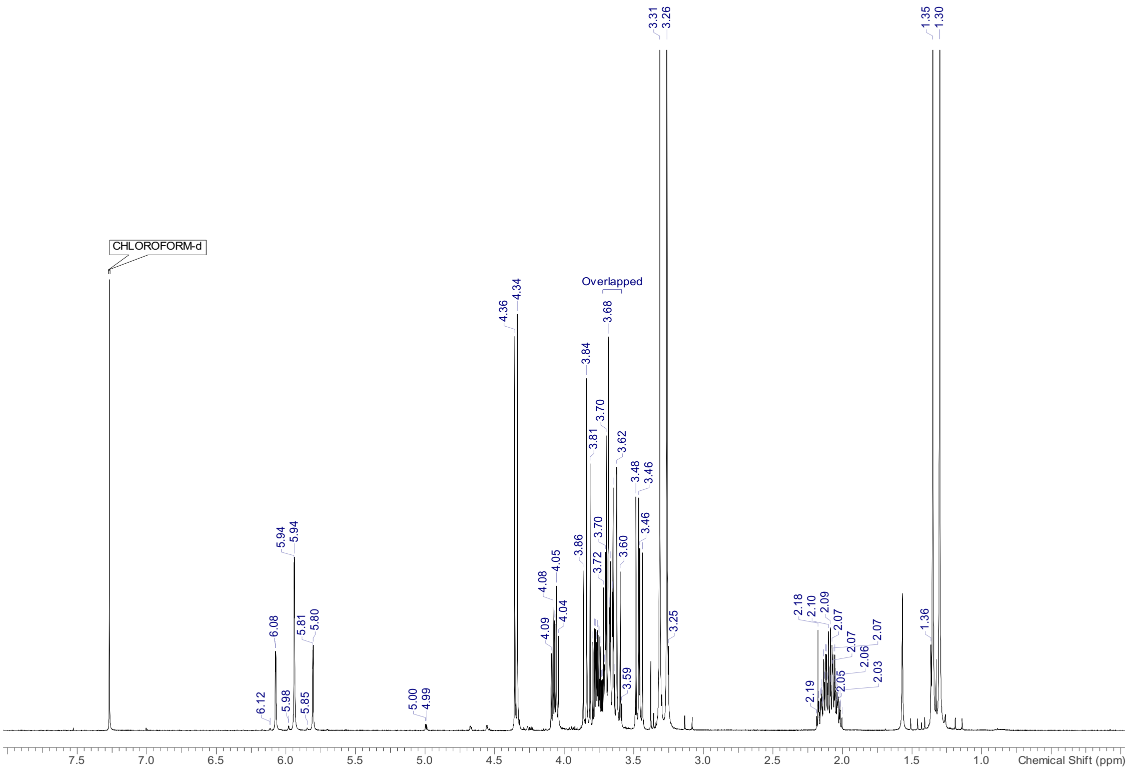

^{13}C{^{1}H} NMR (101 MHz, $CDCl_3$)

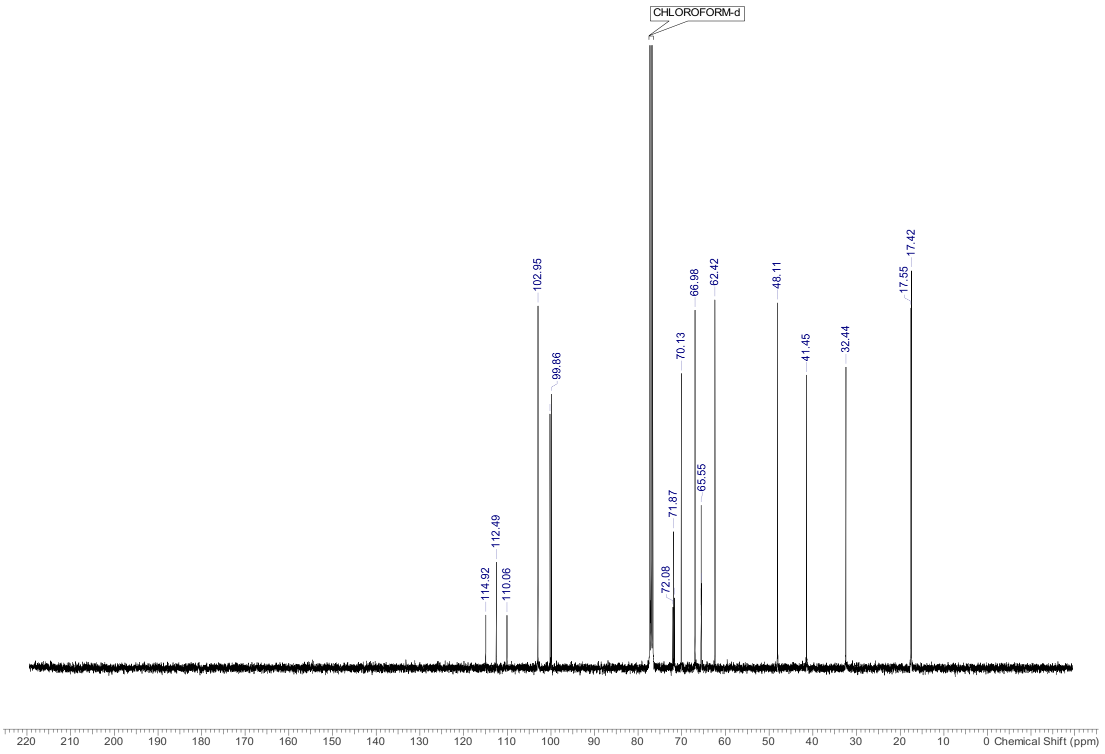

^{19}F NMR (376 MHz, $CDCl_3$)

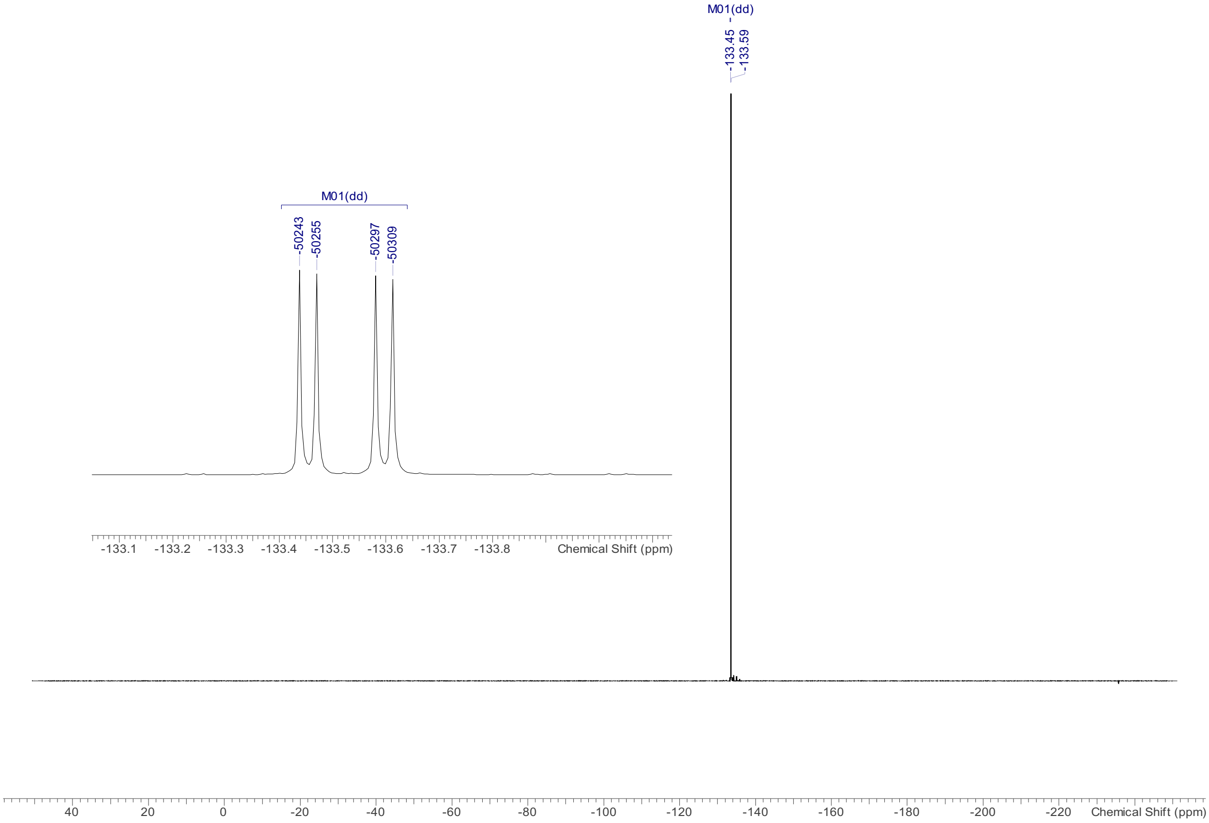

^{19}F{^{1}H} NMR (376 MHz, $CDCl_3$)

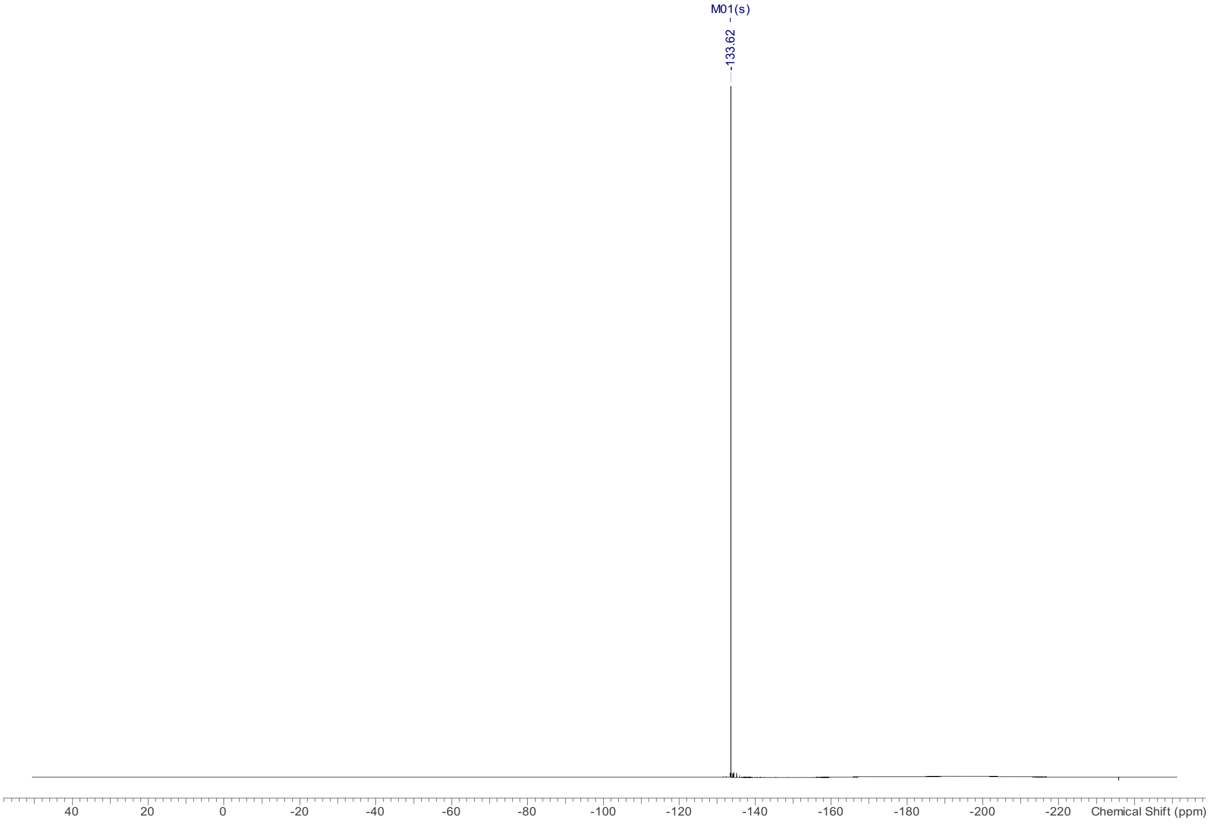

3-Azidopropyl 2,6-dideoxy-2-acetimido-6,6-difluoro-β-D-glucopyranoside (D, 8)

^{1}H NMR (500 MHz, CD_3OD)

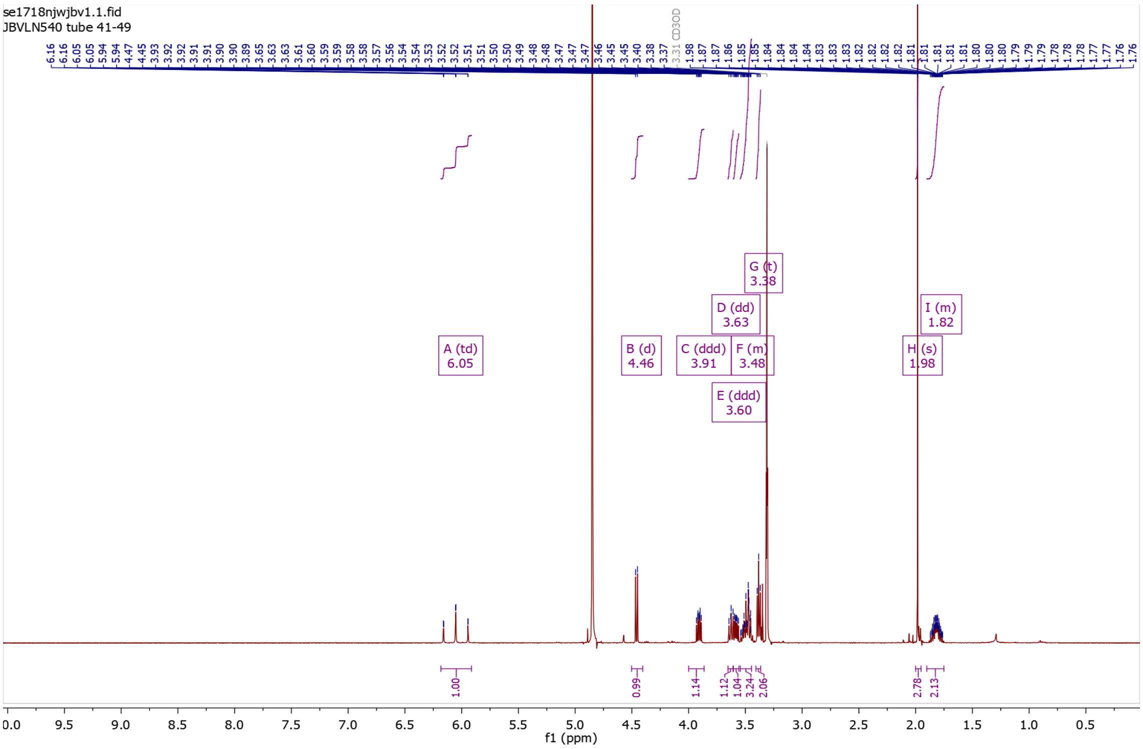

$^{1}H\{^{19}F\}$ NMR (500 MHz, CD_3OD)

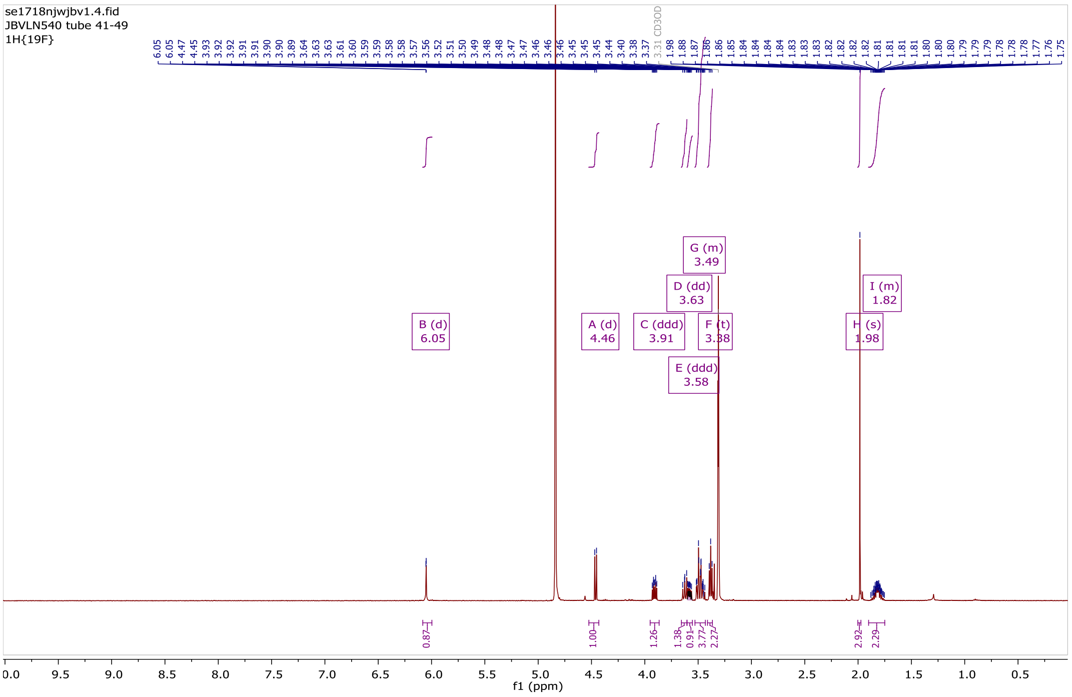

$^{13}C\{^1H\}$ NMR (126 MHz, CD_3OD)

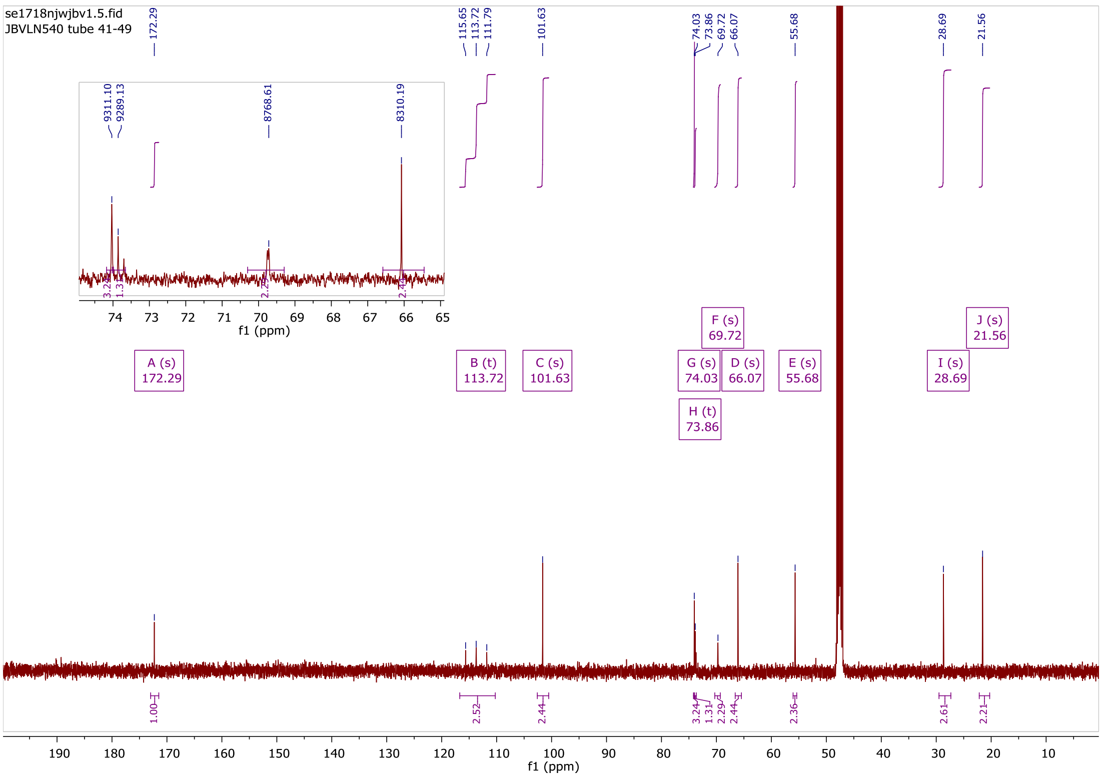

^{19}F NMR (470 MHz, $CDCl_3$)

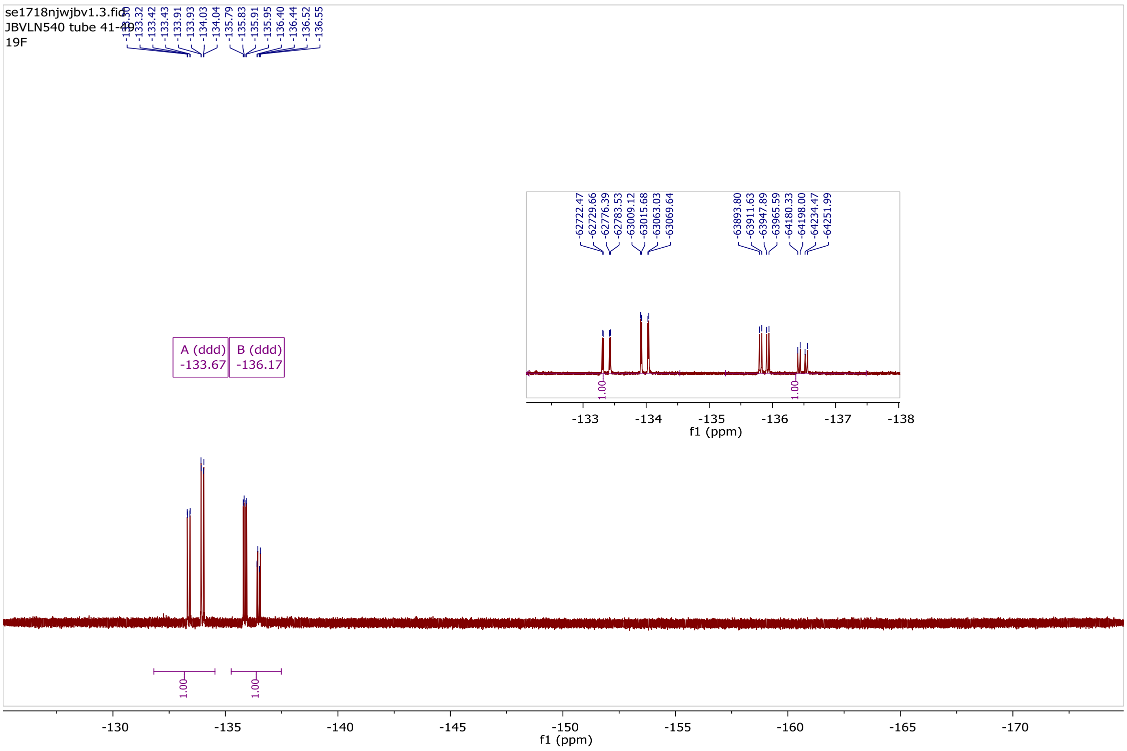

$^{19}F\{^{1}H\}$ NMR (470 MHz, $CDCl_3$)

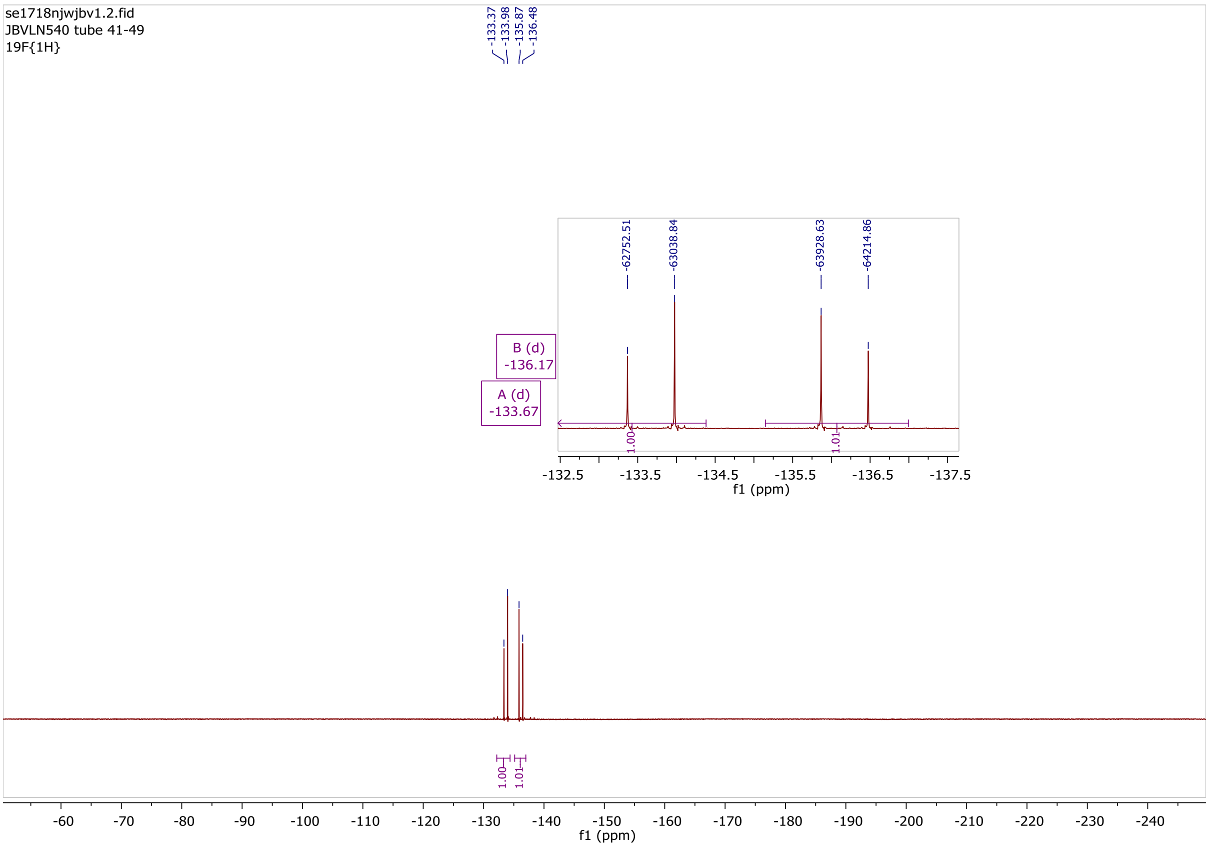

3-Azidoropropyl 2,6-dideoxy-2-trifluoroacetimido-6,6-difluoro-β-D-glucopyranoside (E, 7)

^{1}H NMR (500 MHz, CD_3OD)

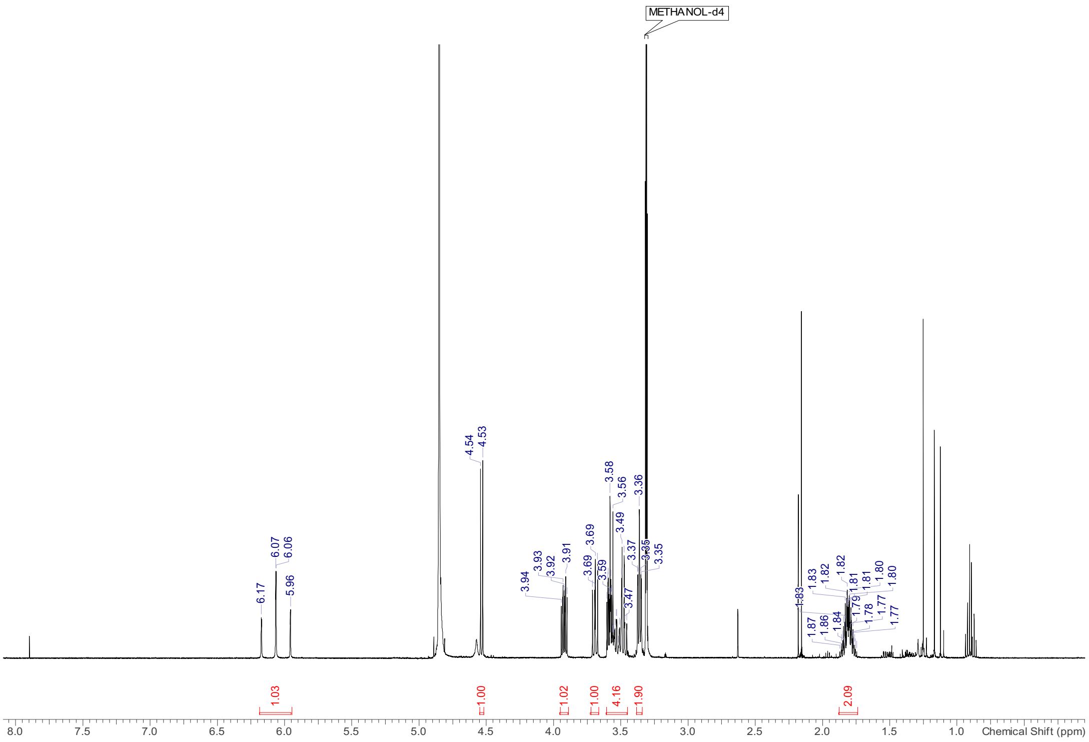

1H{^{19}F} NMR (500 MHz, CD_3OD)

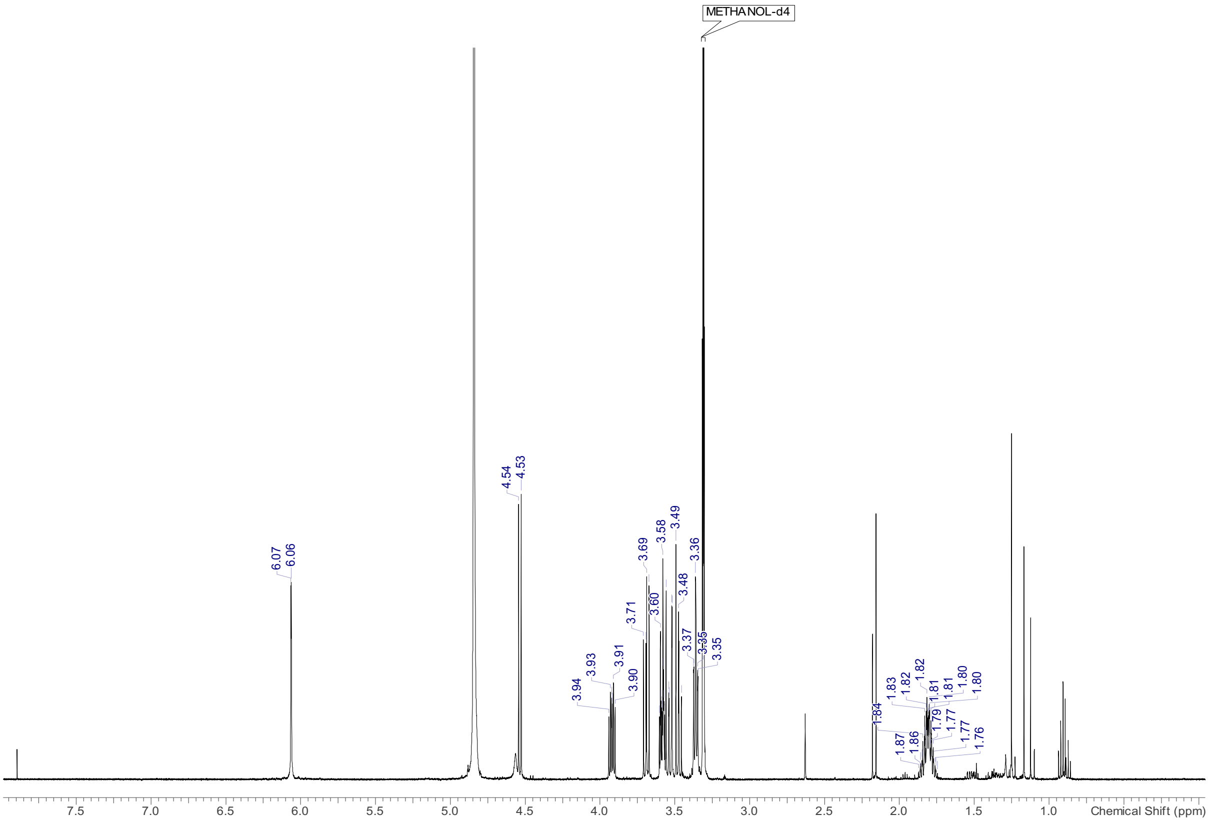

$^{13}C\{^1H\}$ NMR (126 MHz, CD_3OD)

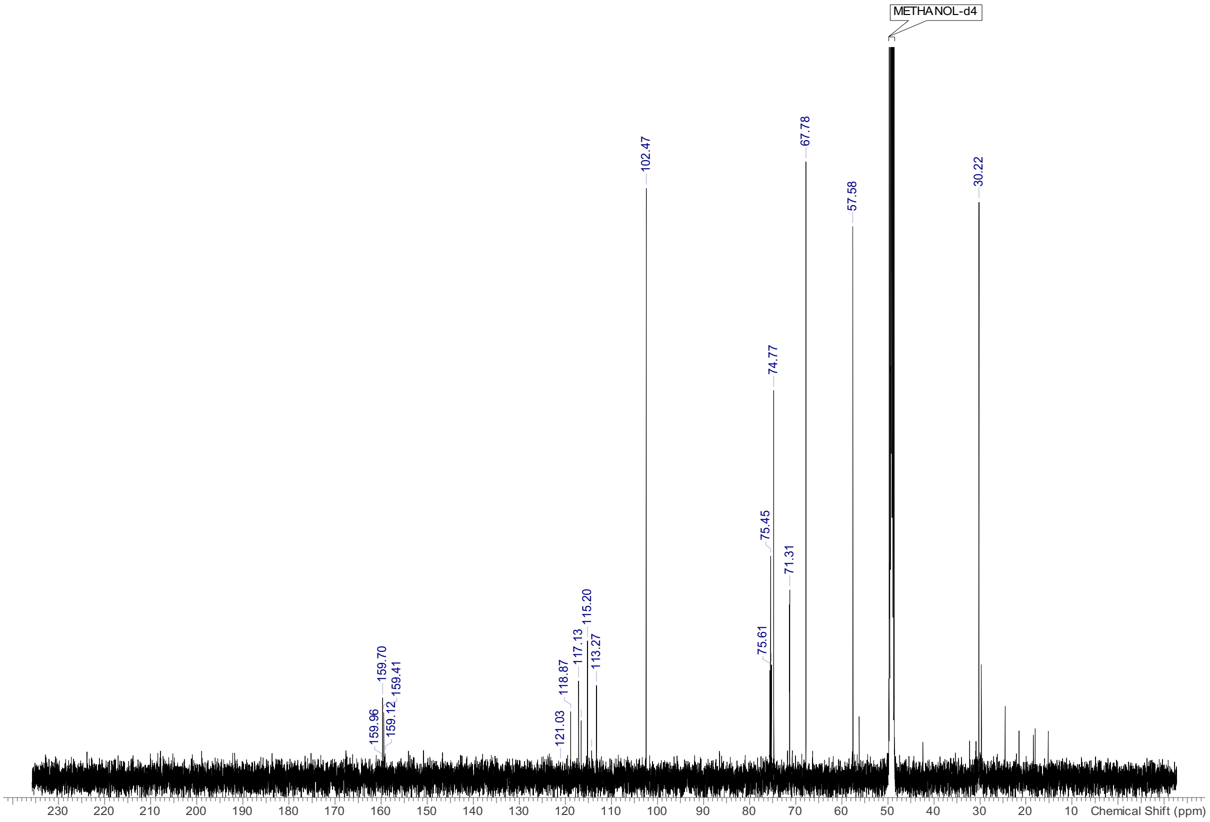

^{19}F NMR (470 MHz, CD_3OD)

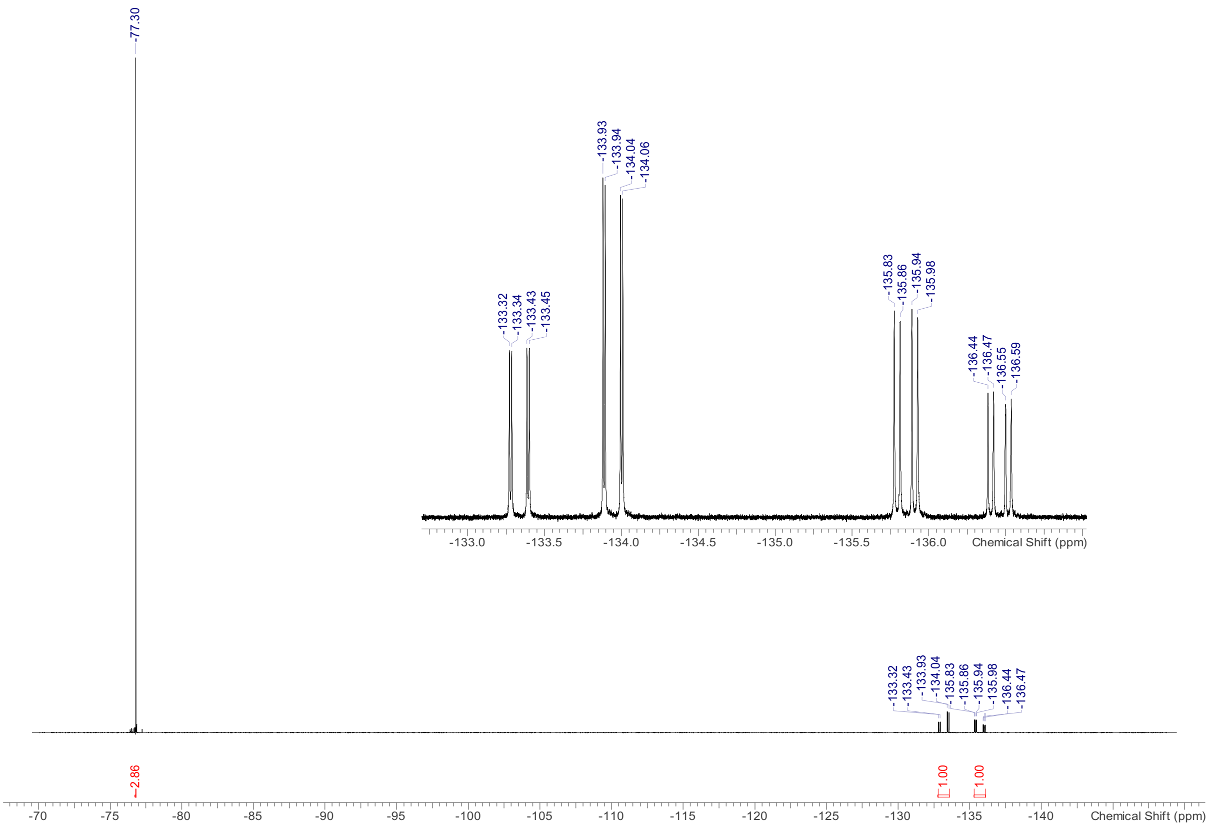

$^{19}F\{^1H\}$ NMR (470 MHz, CD_3OD)

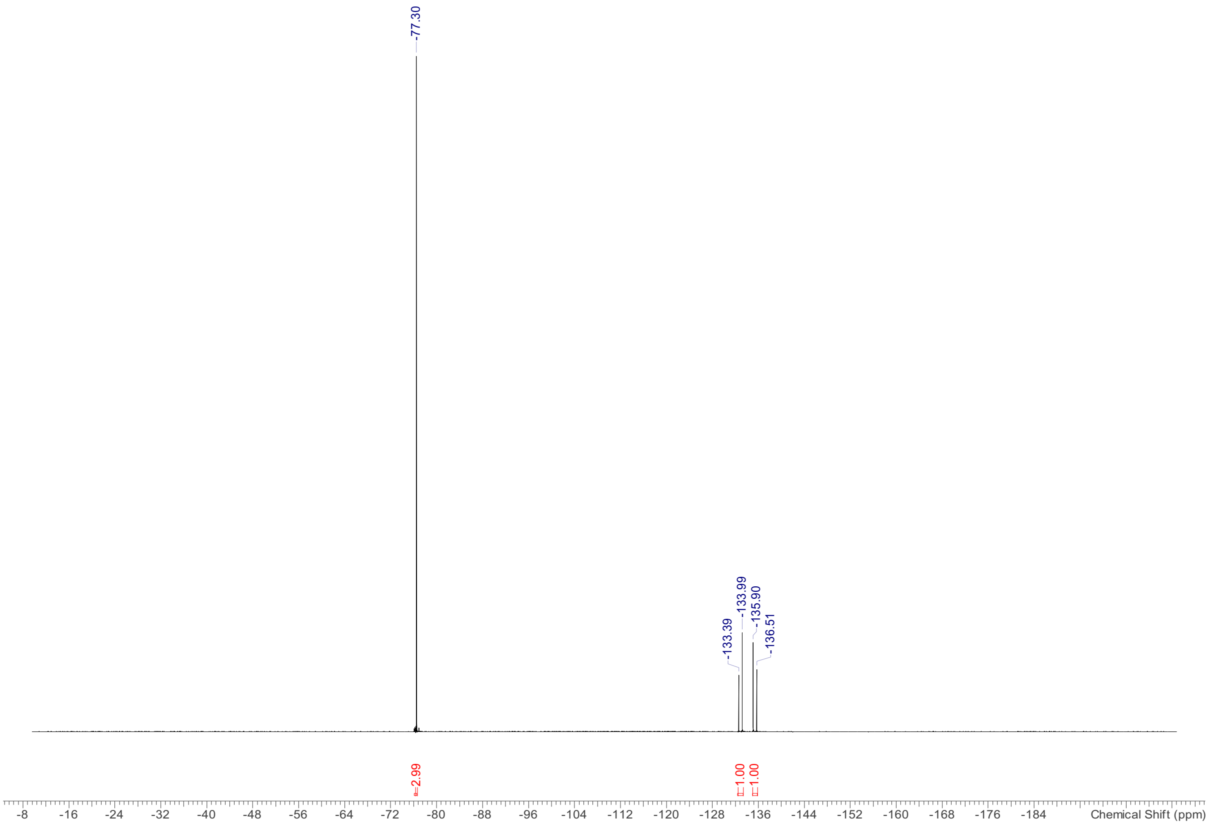

Gal-β(1,3)-GlcNAc-N_3 (15)

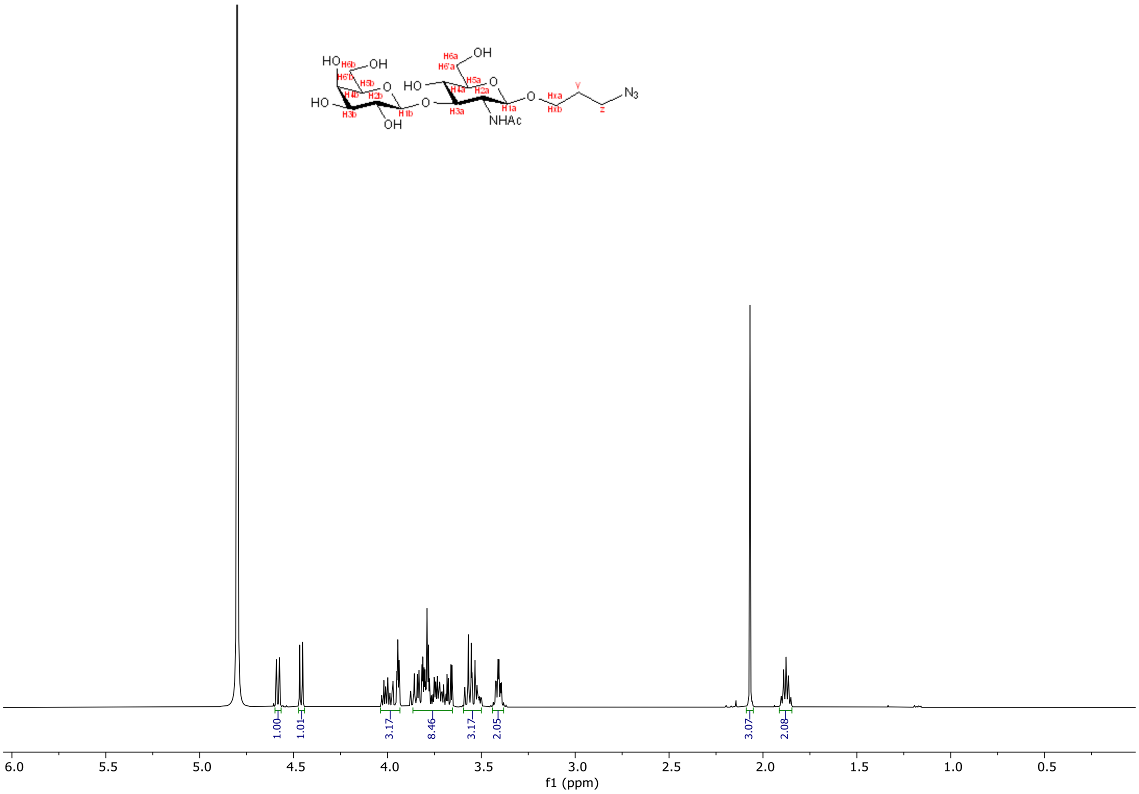

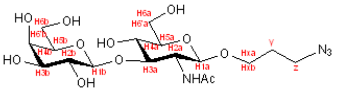

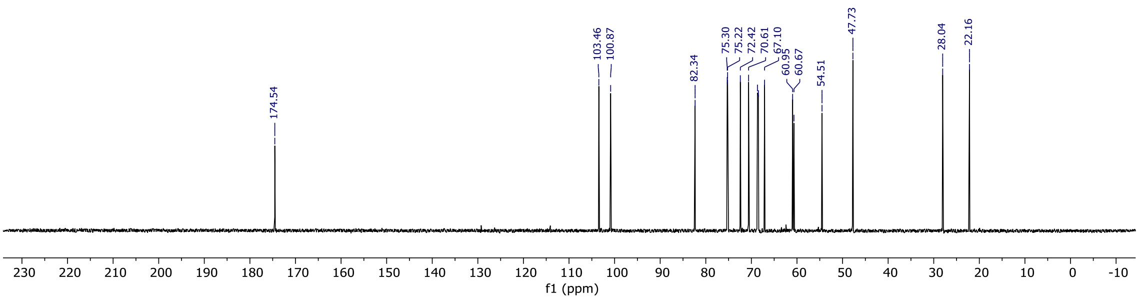

Gal3F-β(1,3)-GlcNAc-N_3 (16)

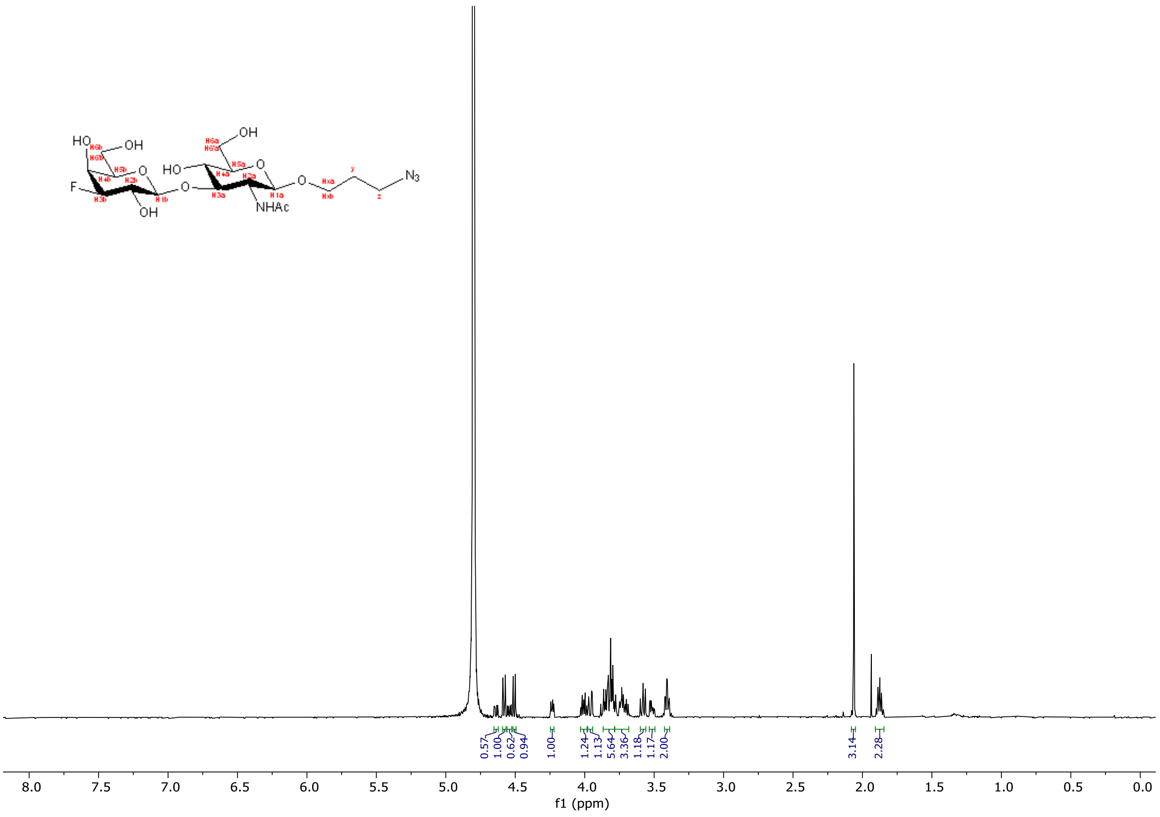

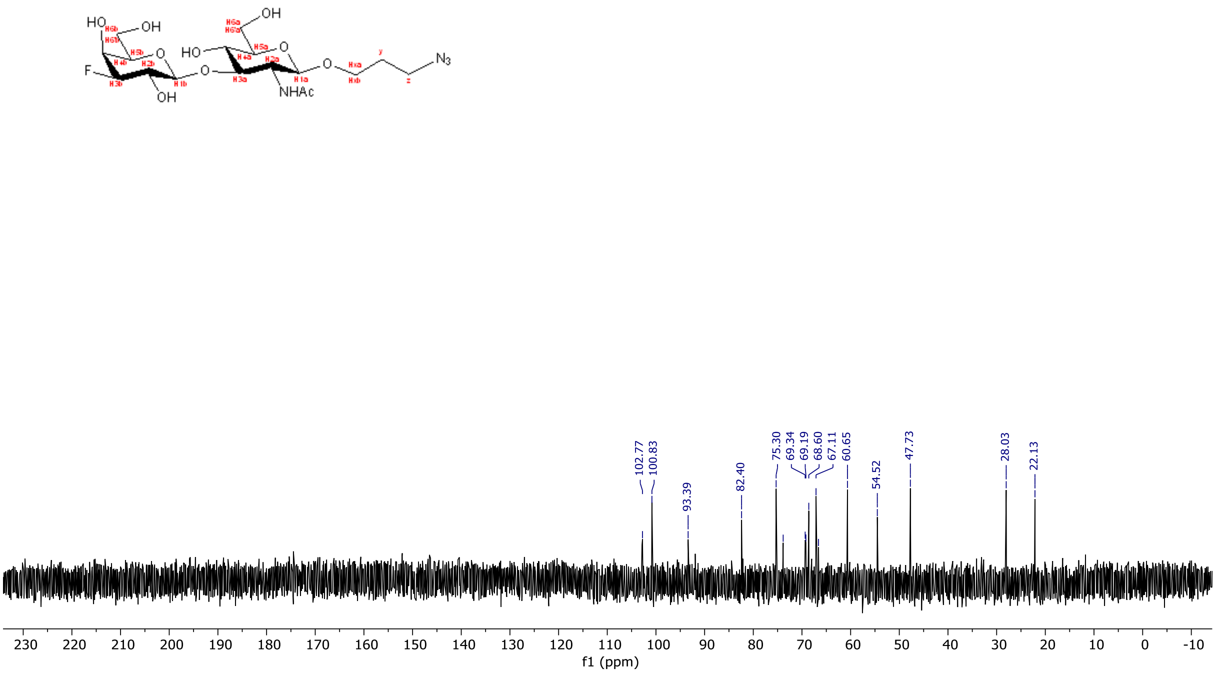

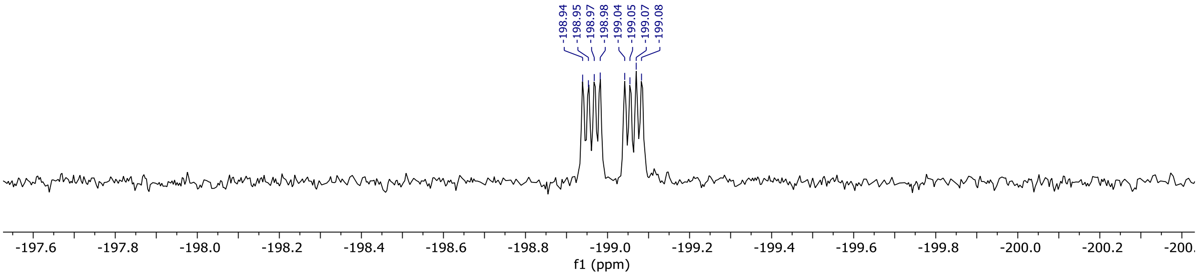

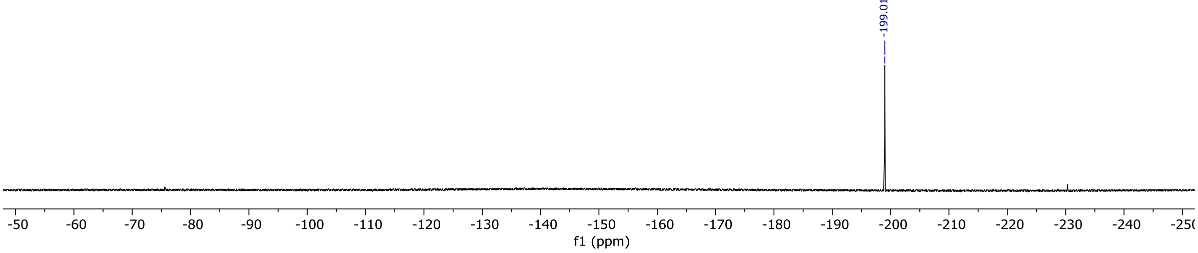

Gal6F-β(1,3)-GlcNAc-N_3 (17)

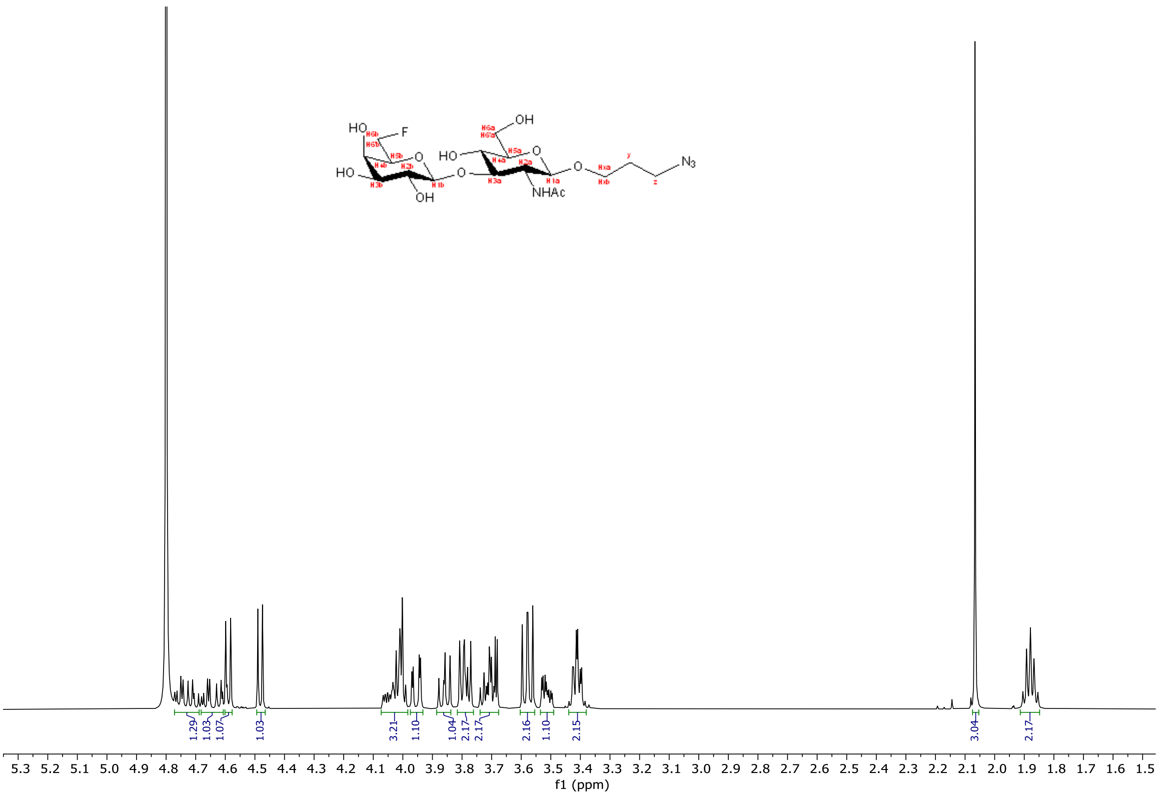
HO
F
HO
OH
HO
O
OH
NHAc
N3
1.29
1.03
1.07
1.03
3.21
1.10
1.04
2.17
2.17
2.16
1.10
2.15
3.04
2.17
5.3 5.2 5.1 5.0 4.9 4.8 4.7 4.6 4.5 4.4 4.3 4.2 4.1 4.0 3.9 3.8 3.7 3.6 3.5 3.4 3.3 3.2 3.1 3.0 2.9 2.8 2.7 2.6 2.5 2.4 2.3 2.2 2.1 2.0 1.9 1.8 1.7 1.6 1.5
f1 (ppm)

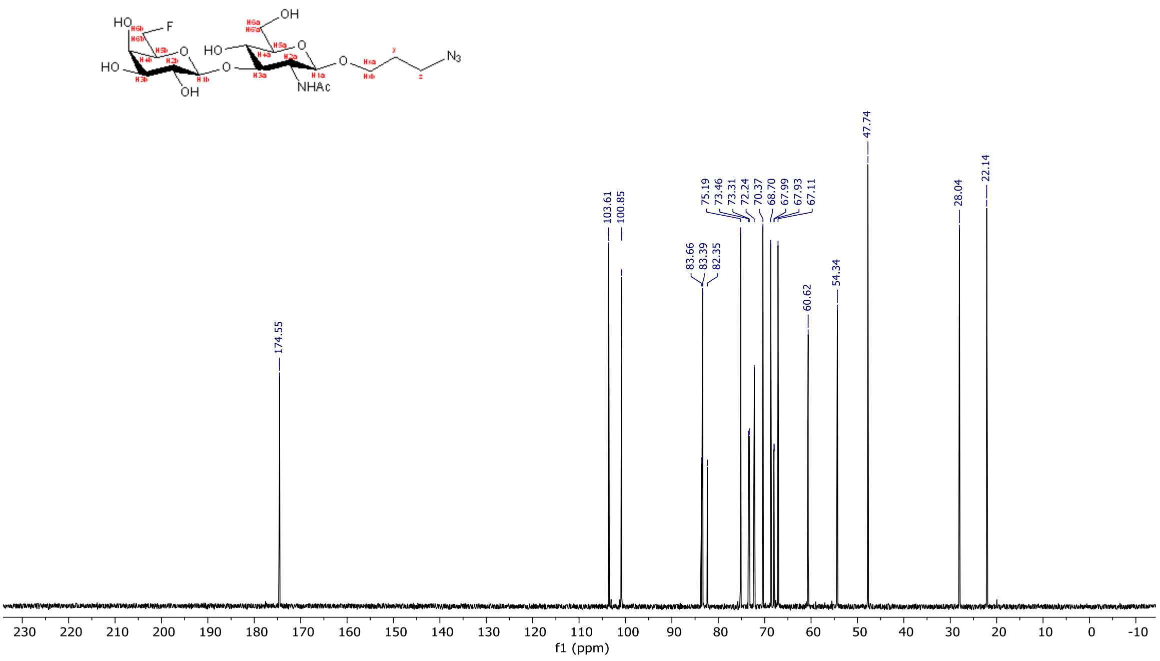
HO
F
HO
OH
HO
O
OH
NHAc
N3
174.55
103.61
100.85
83.66
83.39
82.35
75.19
73.46
73.31
72.24
70.37
68.70
67.99
67.93
67.11
60.62
54.34
47.74
28.04
22.14
230 220 210 200 190 180 170 160 150 140 130 120 110 100 90 80 70 60 50 40 30 20 10 0 -10
f1 (ppm)

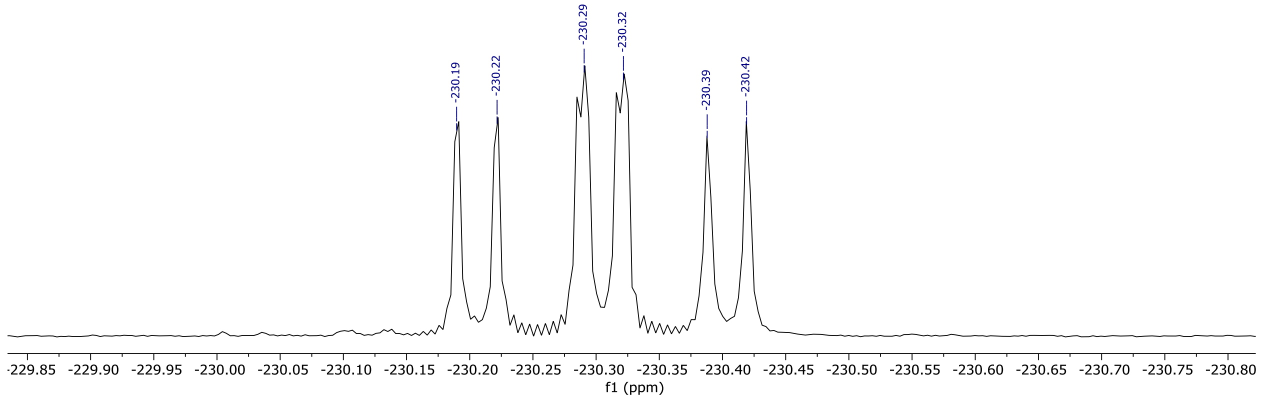

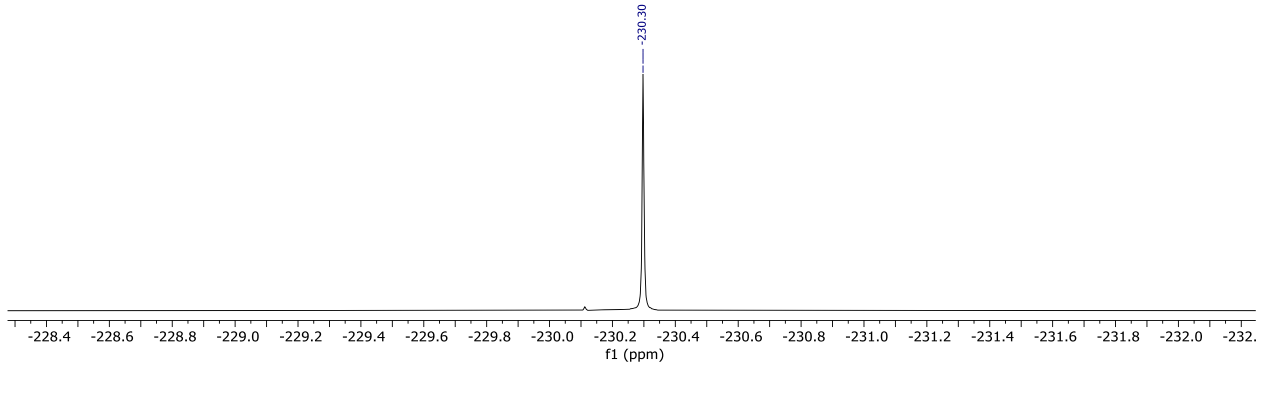

Gal-β(1,3)-6FGlcNAc-N_3 (18)

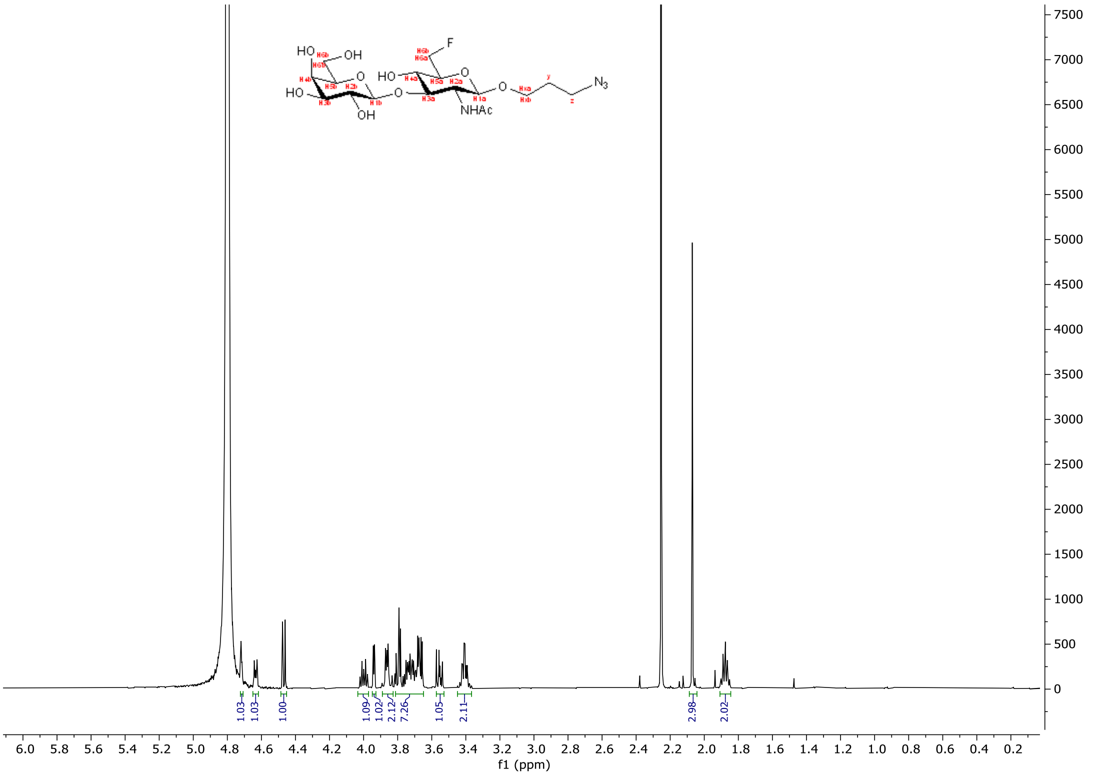
1.03
1.03
1.00
1.09
1.02
2.12
7.26
1.05
2.11
2.98
2.02
f1 (ppm)

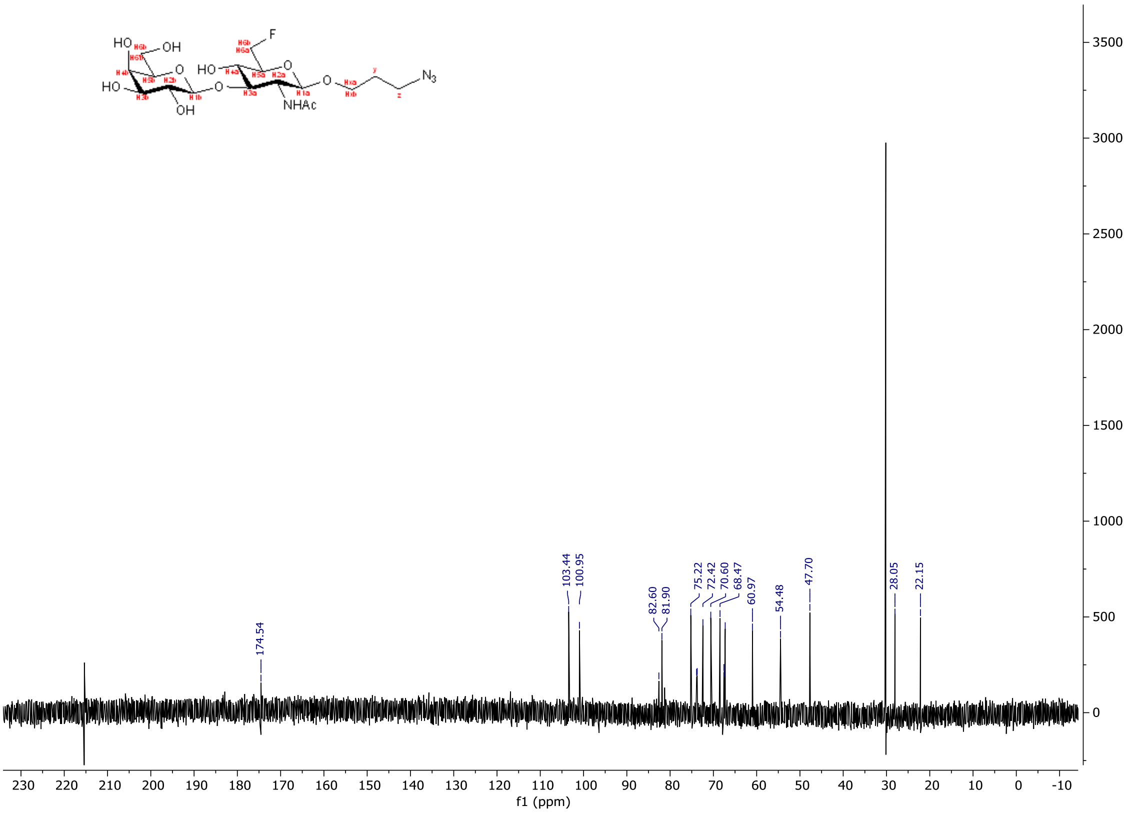
174.54
103.44
100.95
82.60
81.90
75.22
72.42
70.60
68.47
60.97
54.48
47.70
28.05
22.15
f1 (ppm)

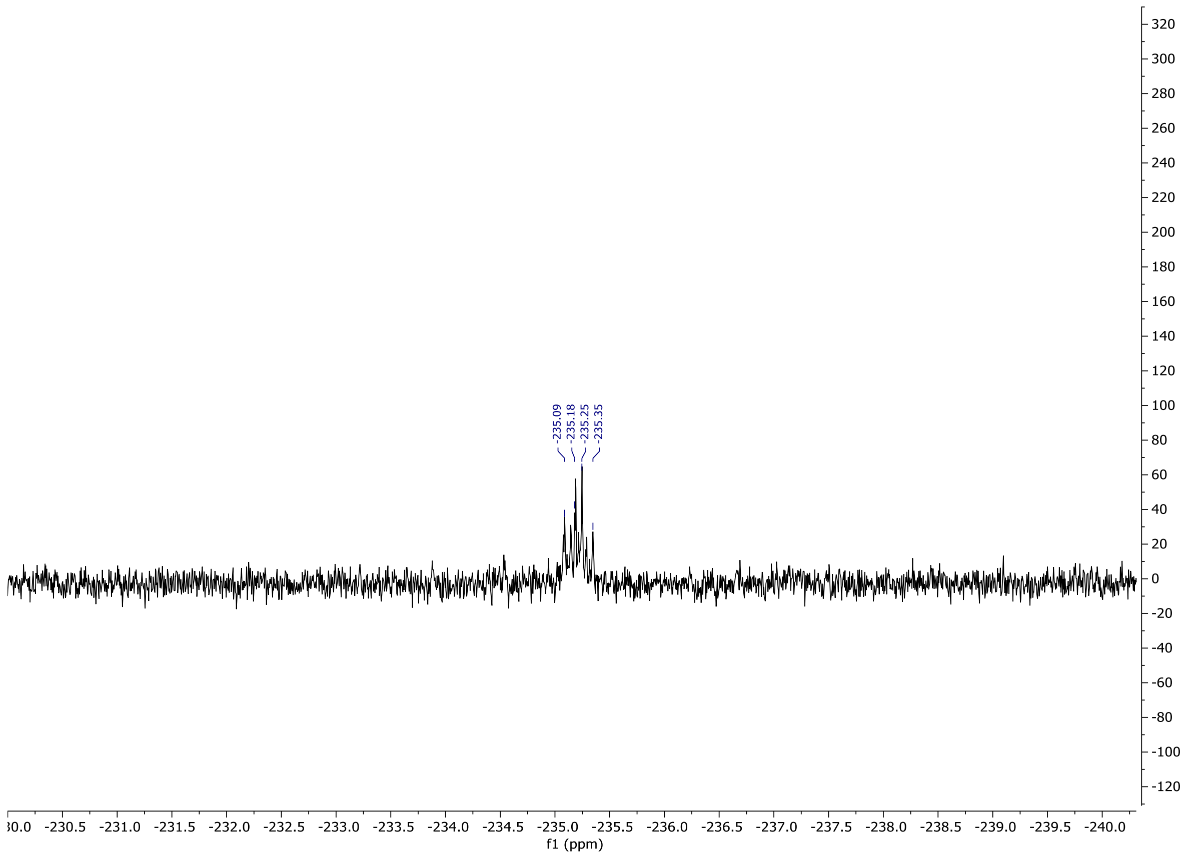

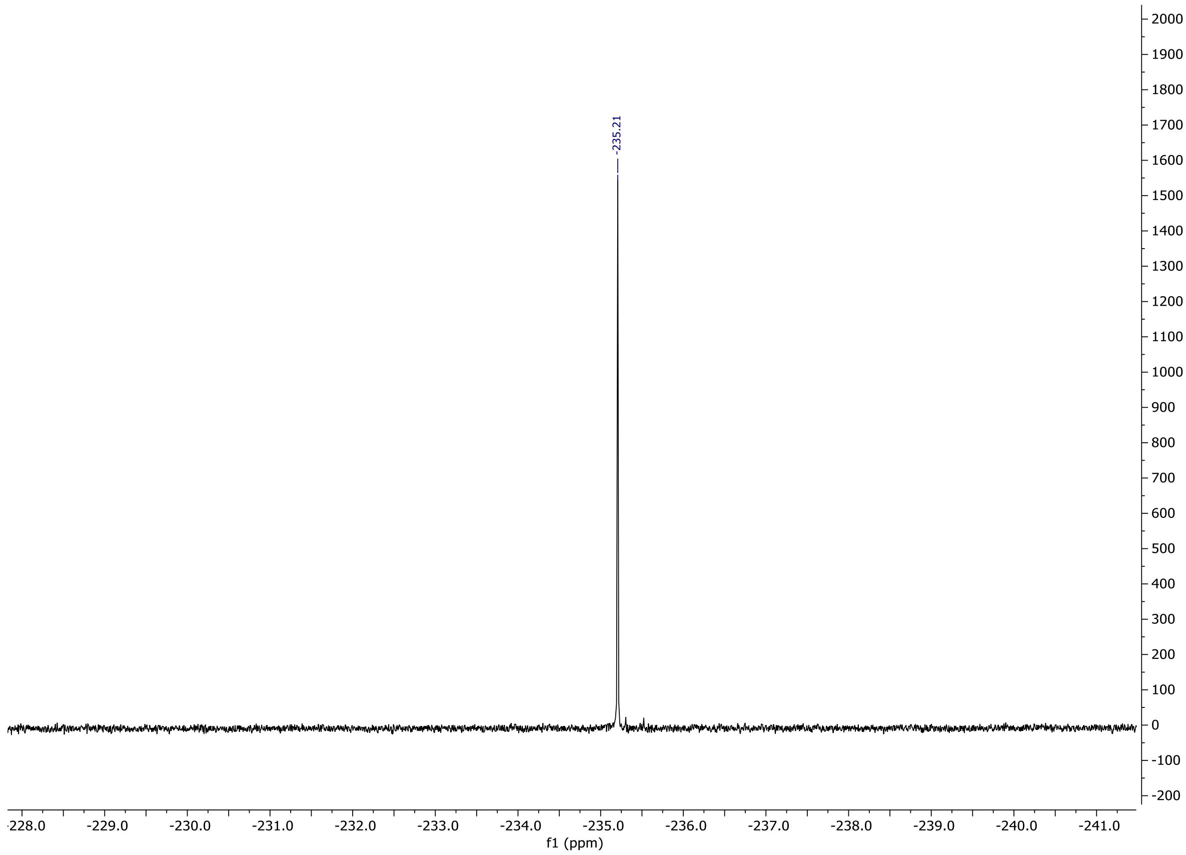

Gal6F-β(1,3)-6FGlcNAc-N_3 (19)

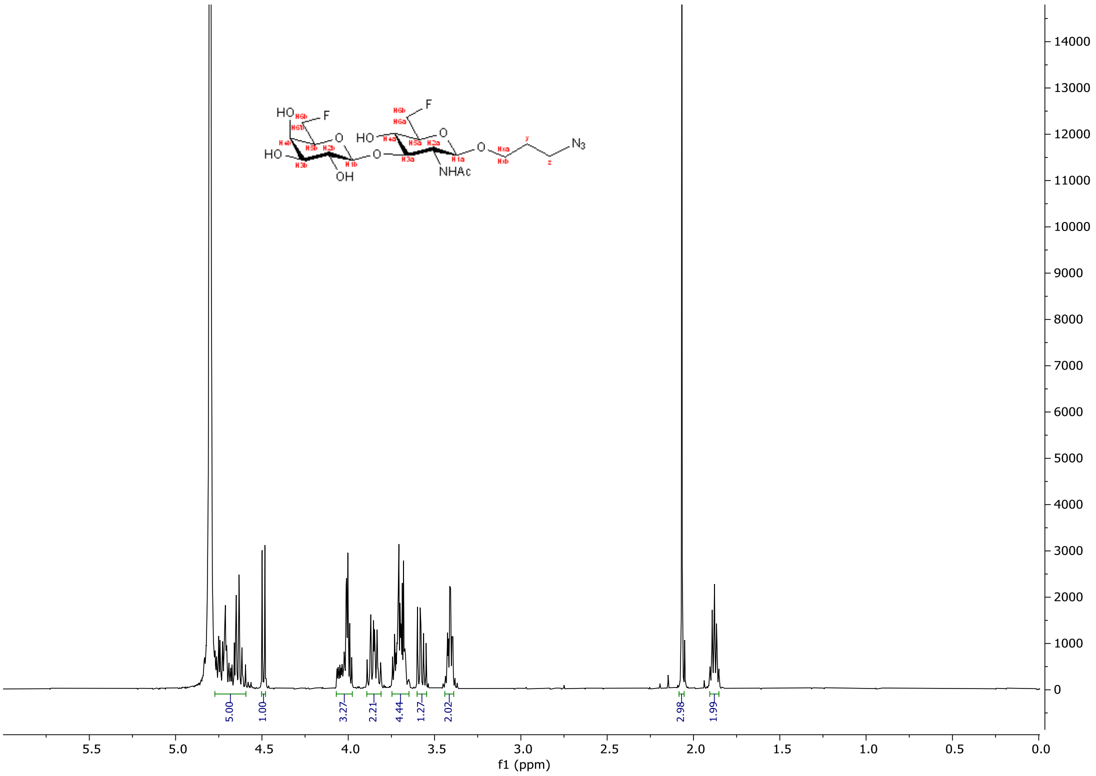

5.00
1.00
3.27
2.21
4.44
1.27
2.02
2.98
1.99
f1 (ppm)

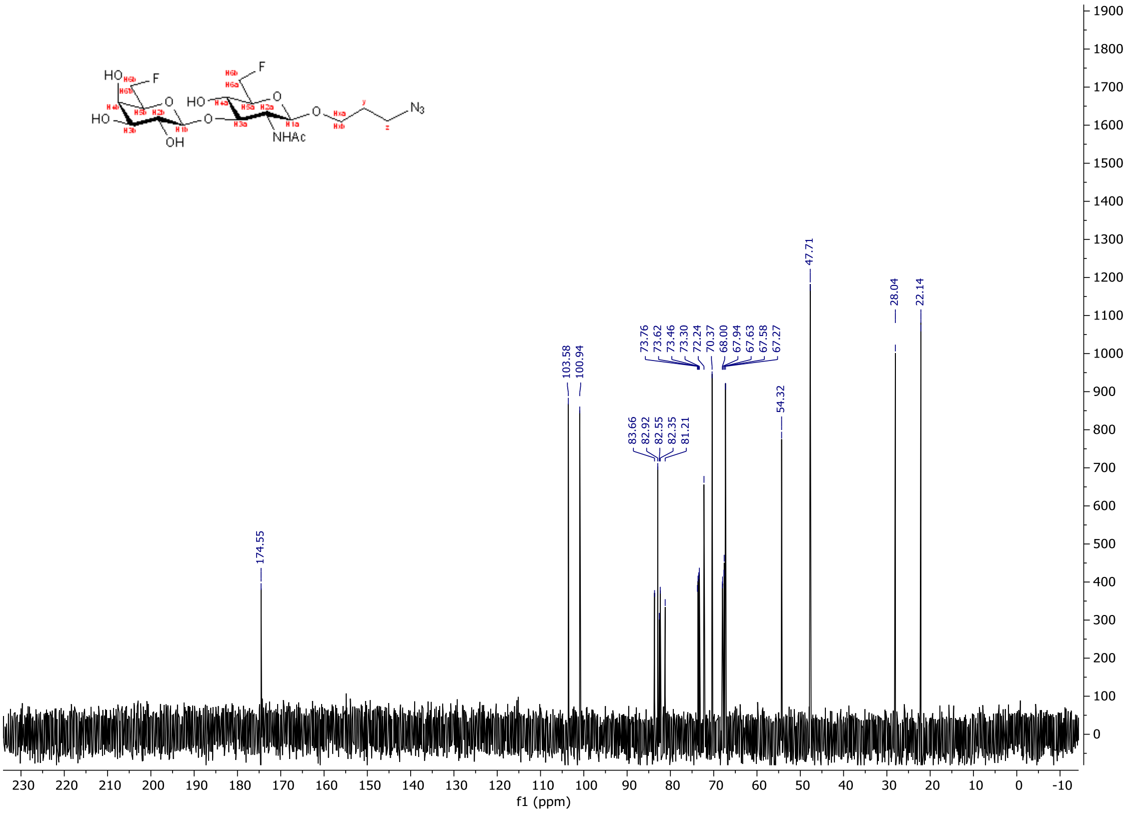

174.55
103.58
100.94
83.66
82.92
82.55
82.35
81.21
73.76
73.62
73.46
73.30
72.24
70.37
68.00
67.94
67.63
67.58
67.27
54.32
47.71
28.04
22.14
f1 (ppm)

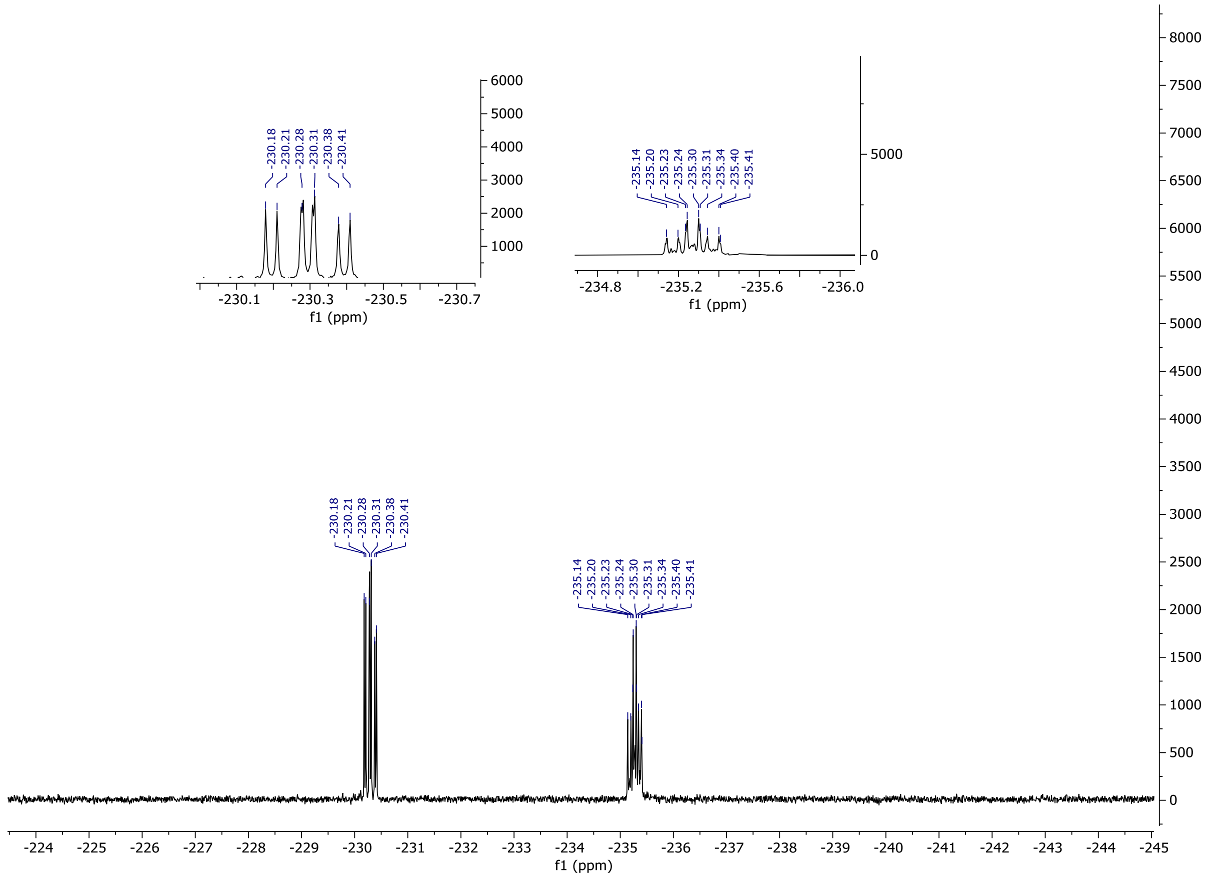
-230.18
-230.21
-230.28
-230.31
-230.38
-230.41
-235.14
-235.20
-235.23
-235.24
-235.30
-235.31
-235.34
-235.40
-235.41
f1 (ppm)

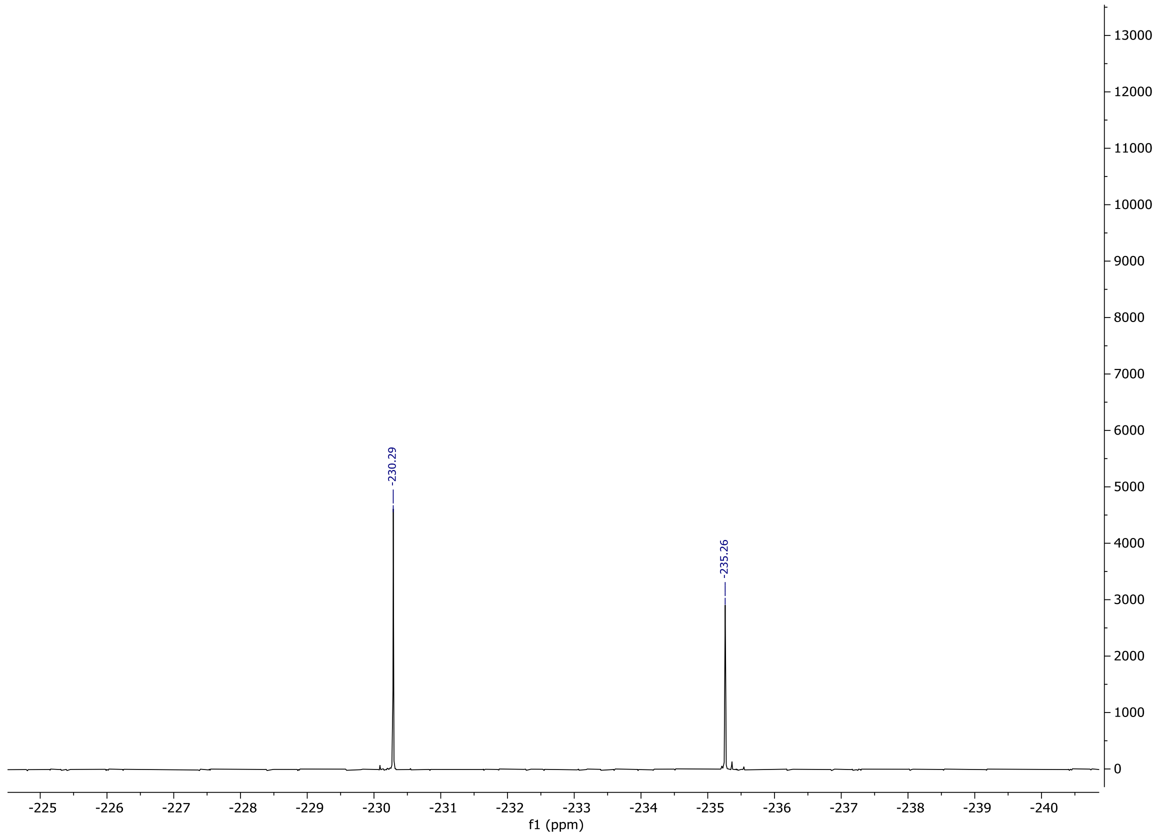
-230.29
-235.26
f1 (ppm)

Gal-β(1,3)-6FGlcNTFA-N_3 (20)

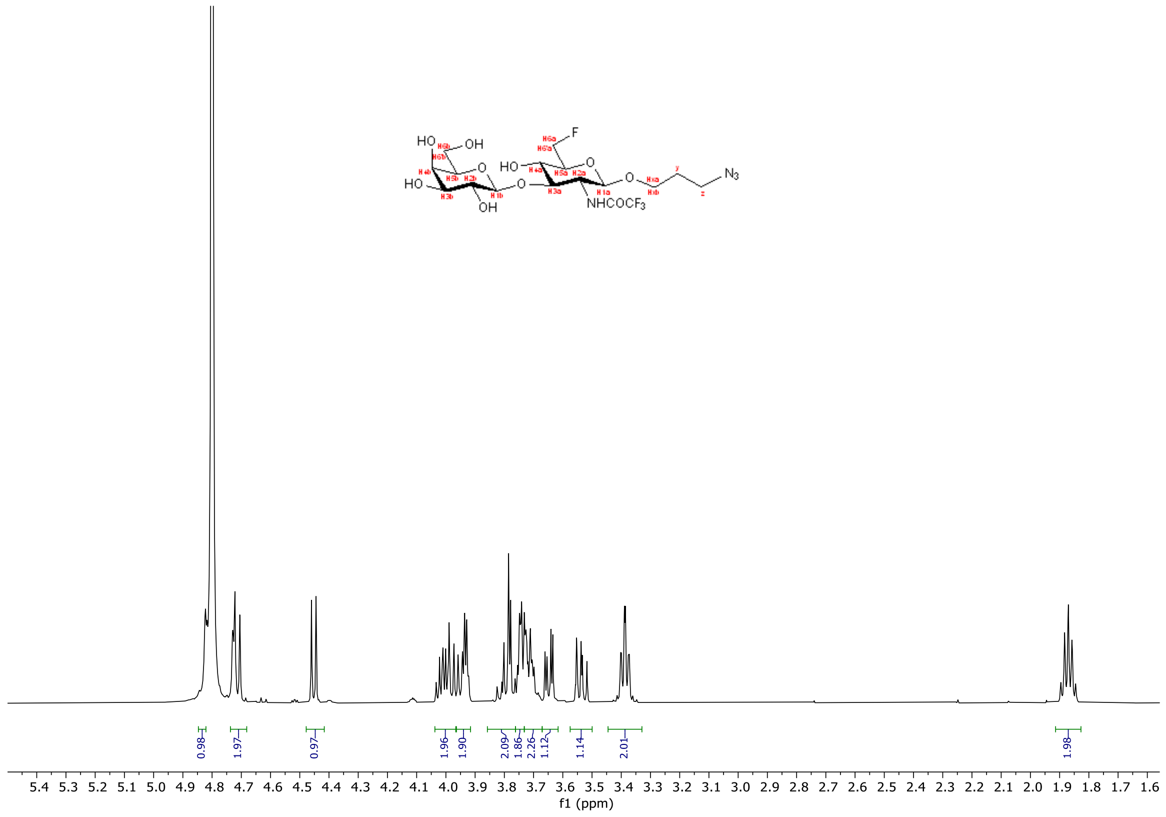

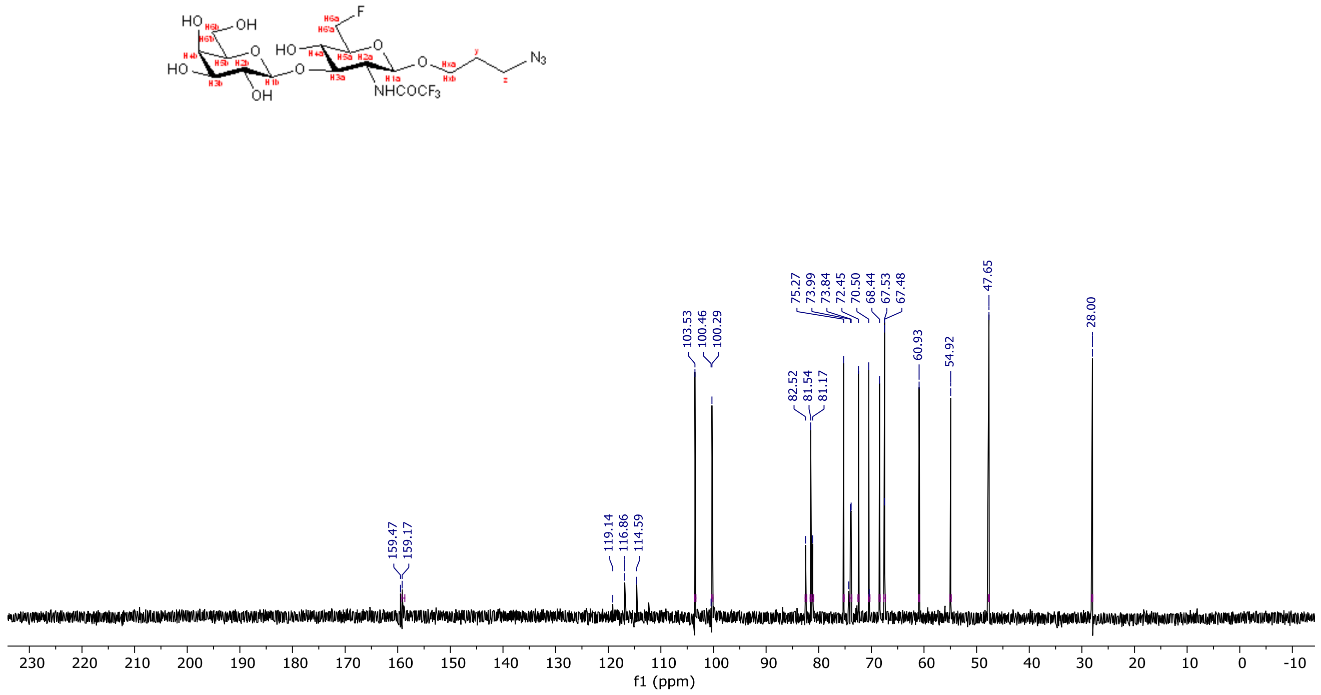

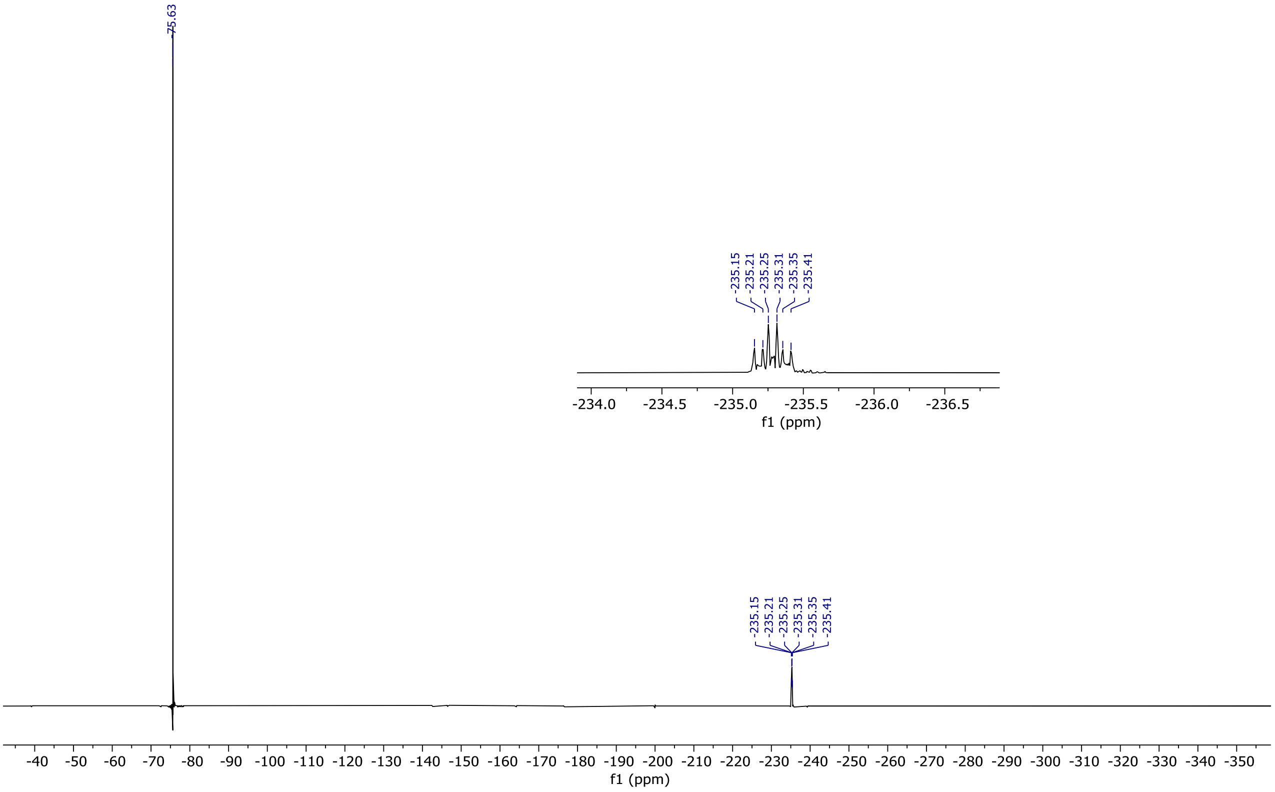

-75.63
-235.15
-235.21
-235.25
-235.31
-235.35
-235.41
-234.0 -234.5 -235.0 -235.5 -236.0 -236.5
f1 (ppm)
-40 -50 -60 -70 -80 -90 -100 -110 -120 -130 -140 -150 -160 -170 -180 -190 -200 -210 -220 -230 -240 -250 -260 -270 -280 -290 -300 -310 -320 -330 -340 -350
f1 (ppm)

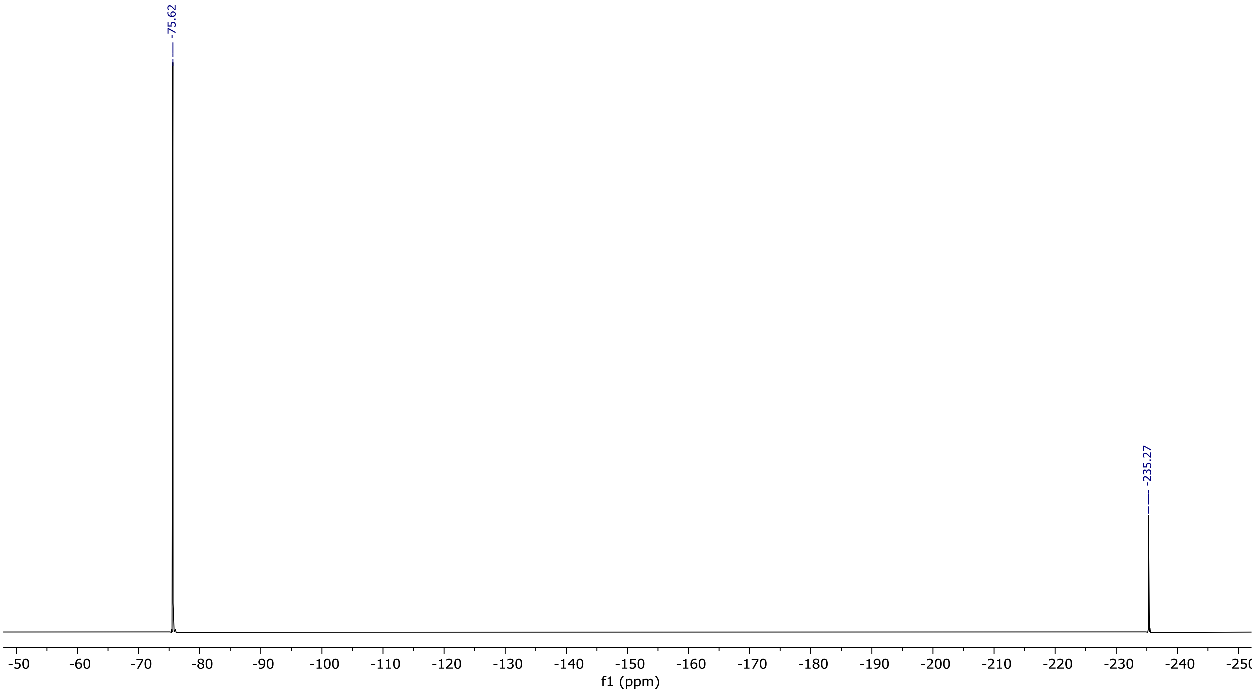

-75.62
-235.27
-50 -60 -70 -80 -90 -100 -110 -120 -130 -140 -150 -160 -170 -180 -190 -200 -210 -220 -230 -240
f1 (ppm)

Gal6F-β(1,3)-6FGlcNTFA-N₃ (21)

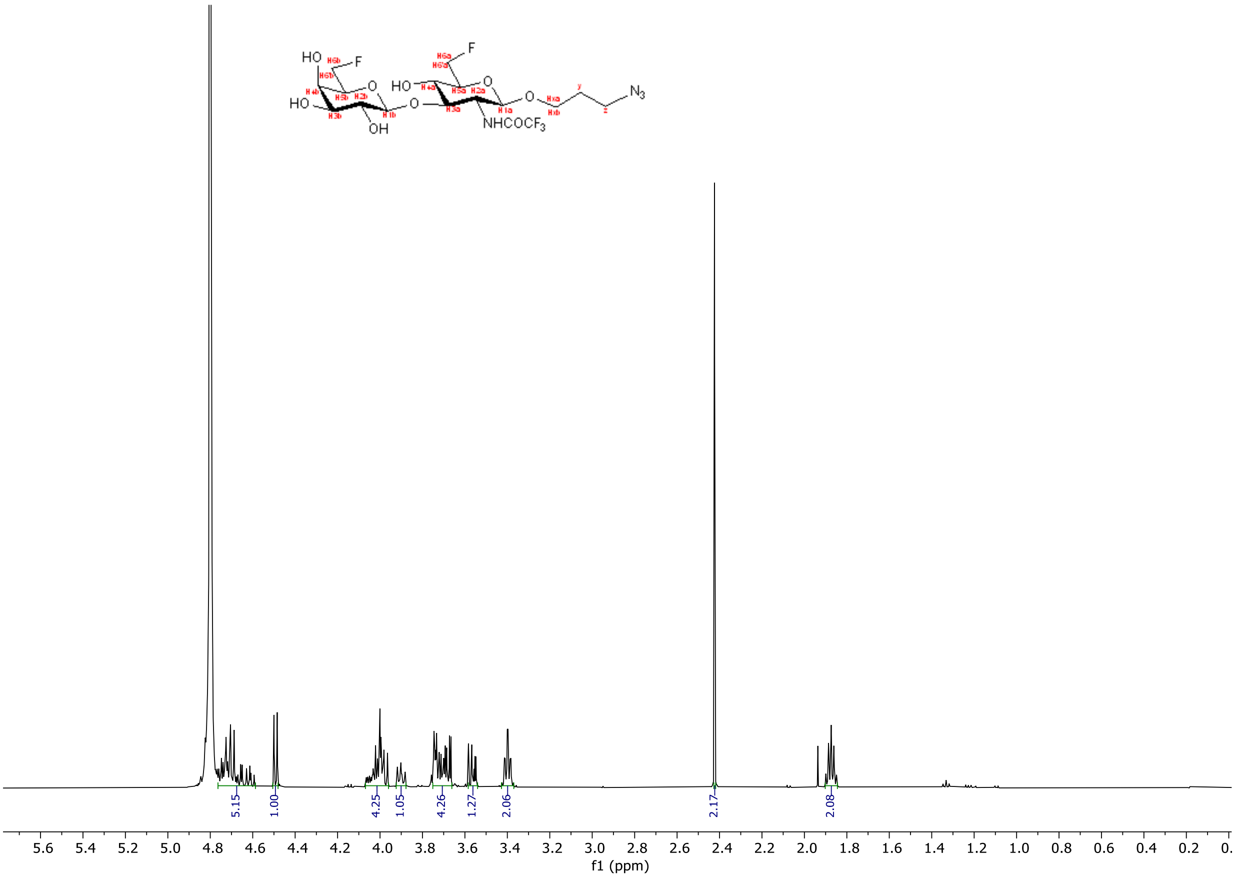

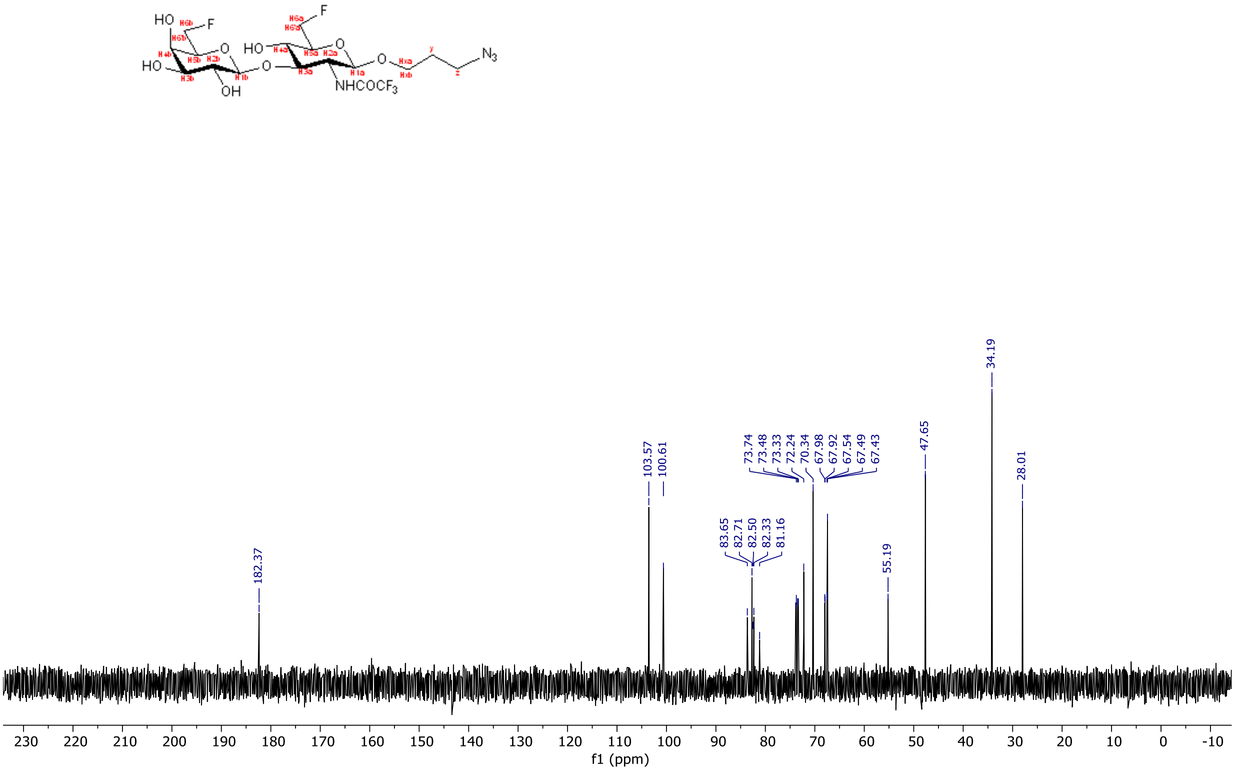

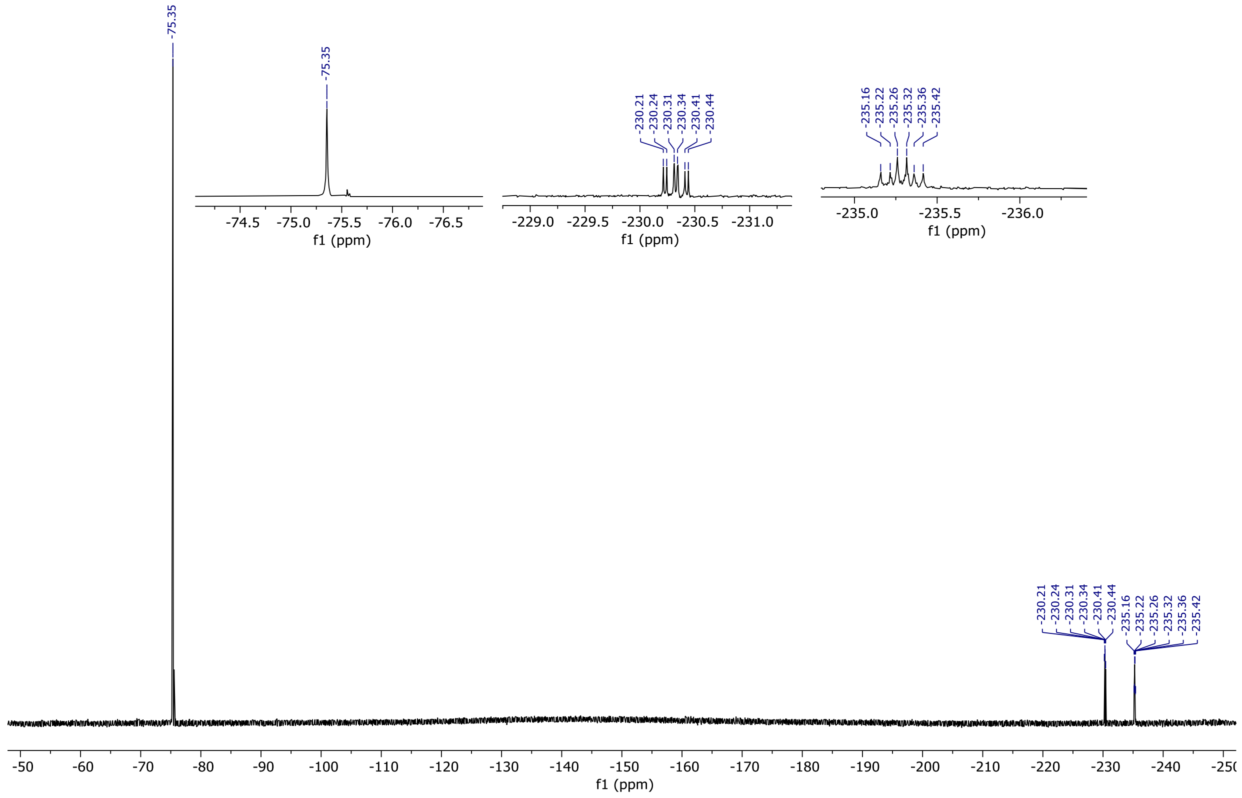
-75.35
-75.35
-74.5 -75.0 -75.5 -76.0 -76.5
f1 (ppm)
-230.21 -230.24 -230.31 -230.34 -230.41 -230.44
-229.0 -229.5 -230.0 -230.5 -231.0
f1 (ppm)
-235.16 -235.22 -235.26 -235.32 -235.36 -235.42
-235.0 -235.5 -236.0
f1 (ppm)
-230.21 -230.24 -230.31 -230.34 -230.41 -230.44 -235.16 -235.22 -235.26 -235.32 -235.36 -235.42
-50 -60 -70 -80 -90 -100 -110 -120 -130 -140 -150 -160 -170 -180 -190 -200 -210 -220 -230 -240
f1 (ppm)

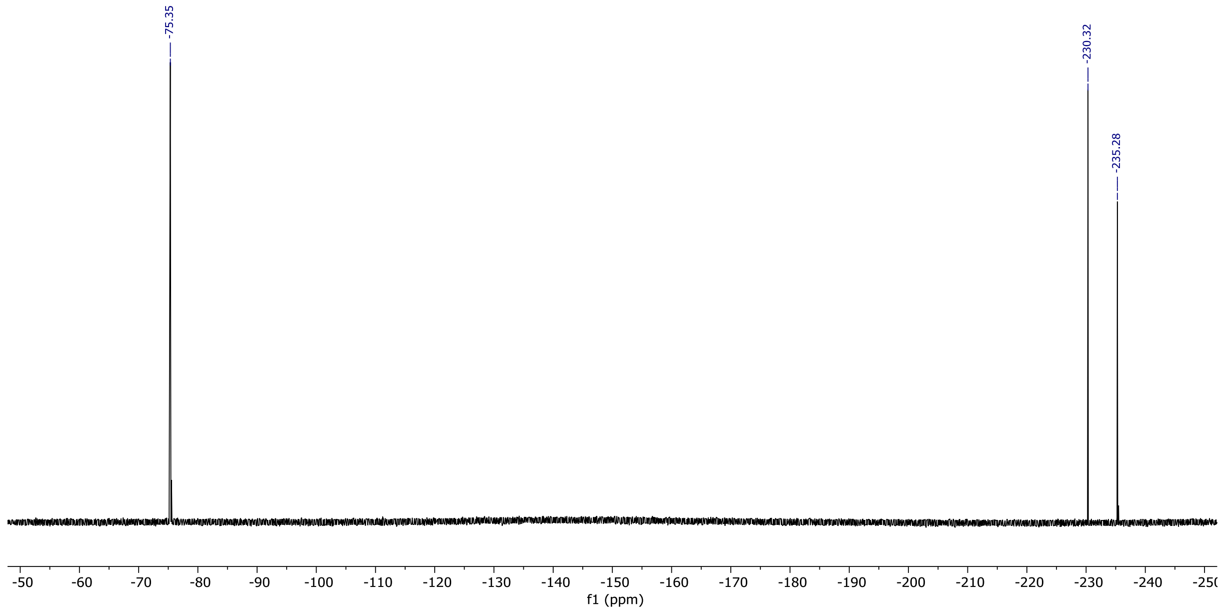
-75.35
-230.32
-235.28
-50 -60 -70 -80 -90 -100 -110 -120 -130 -140 -150 -160 -170 -180 -190 -200 -210 -220 -230 -240
f1 (ppm)

Gal-β(1,3)-6,6diFGlcNAc-N_3 (22)

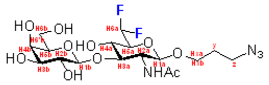

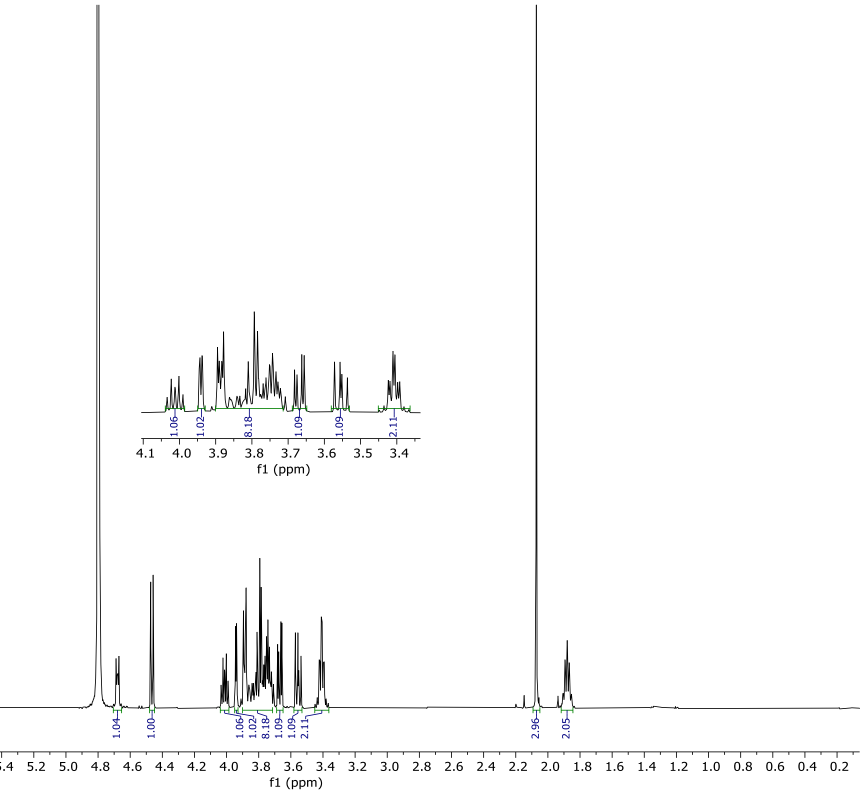

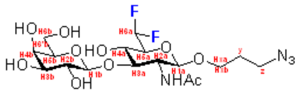

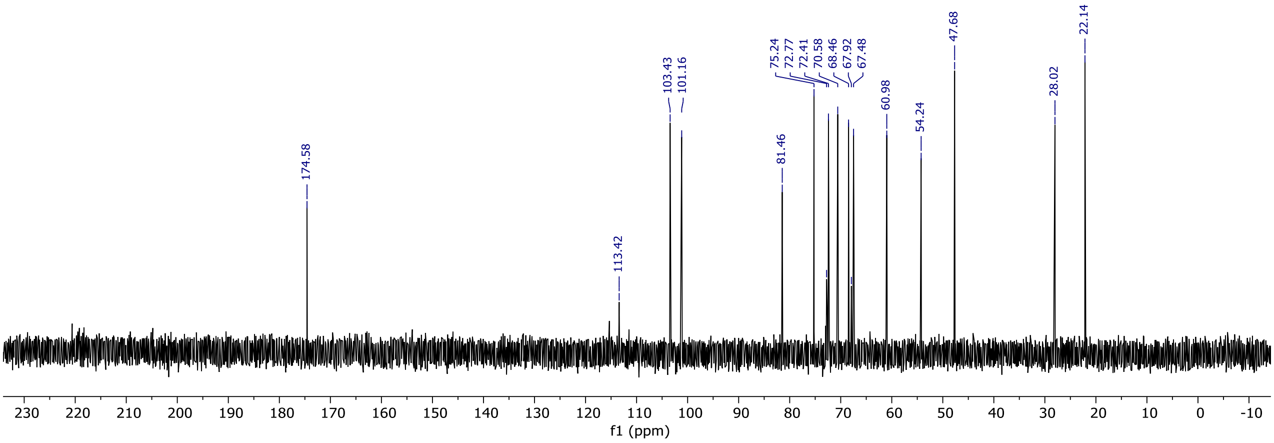

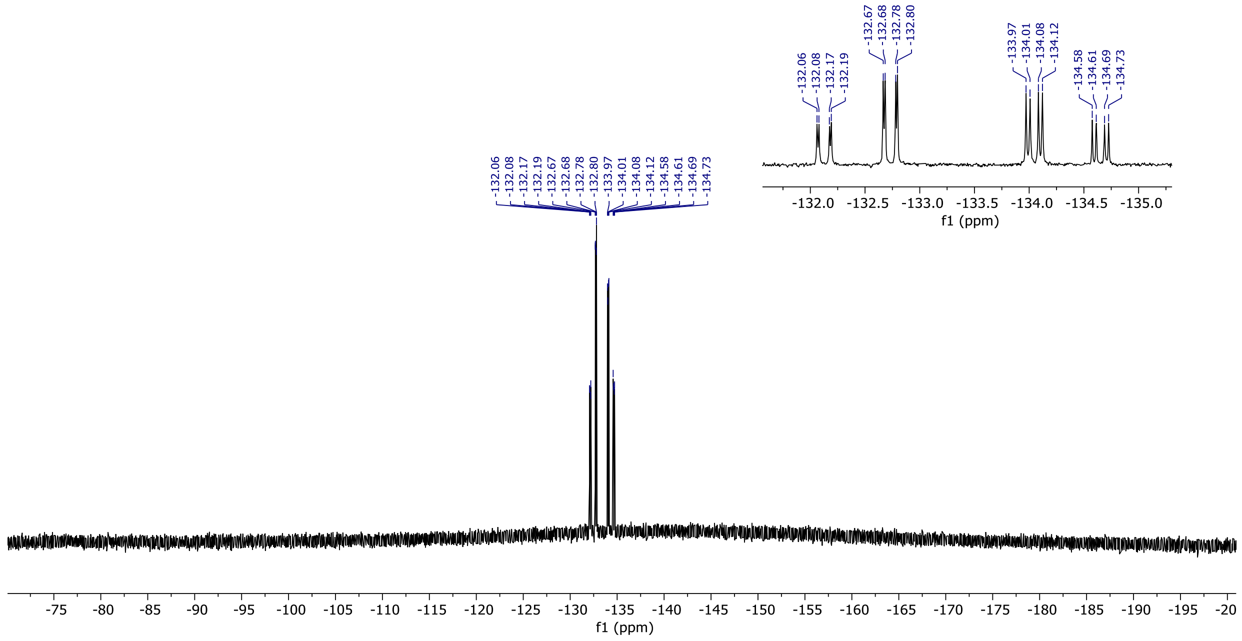
-132.06
-132.08
-132.17
-132.19
-132.67
-132.68
-132.78
-132.80
-133.97
-134.01
-134.08
-134.12
-134.58
-134.61
-134.69
-134.73
f1 (ppm)

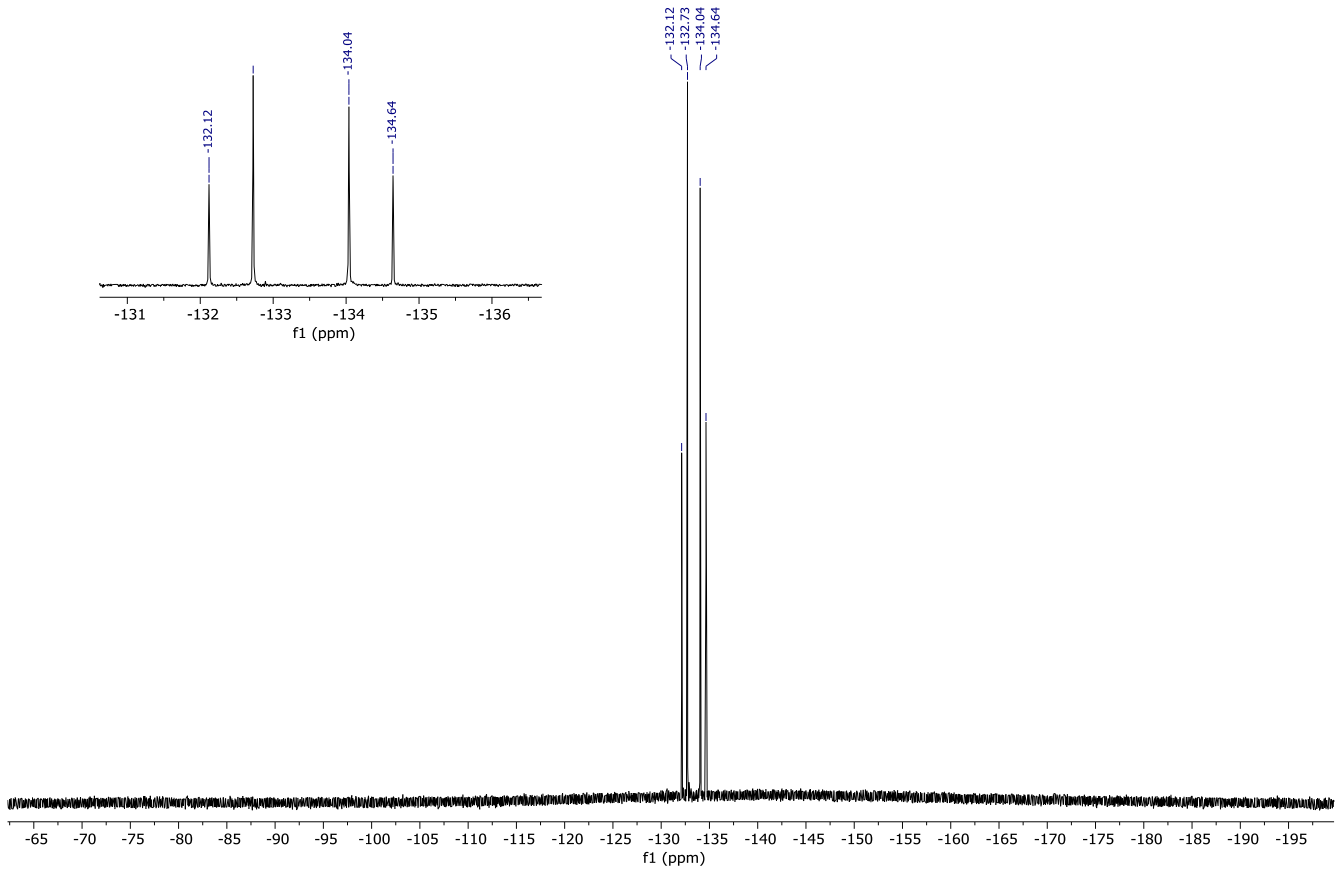
-132.12
-132.73
-134.04
-134.64
f1 (ppm)

Galβ1,3-6,6diFGlcNTFA-N₃ (23)

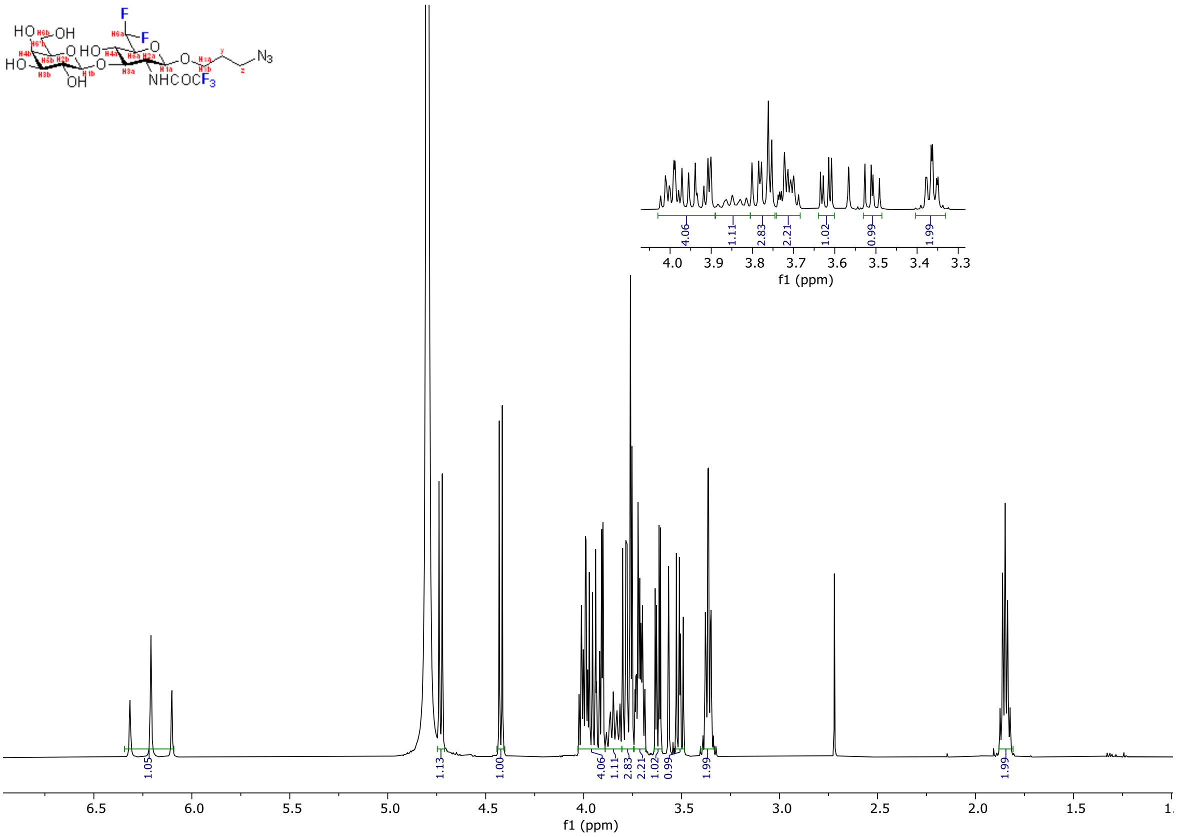

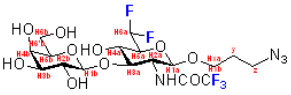

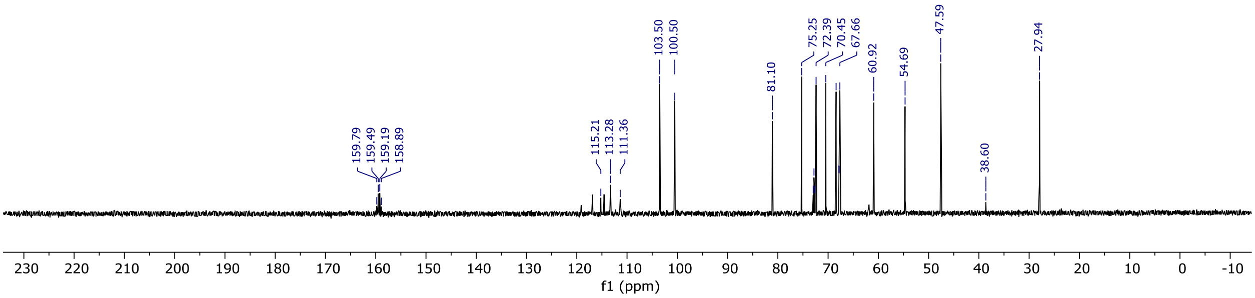

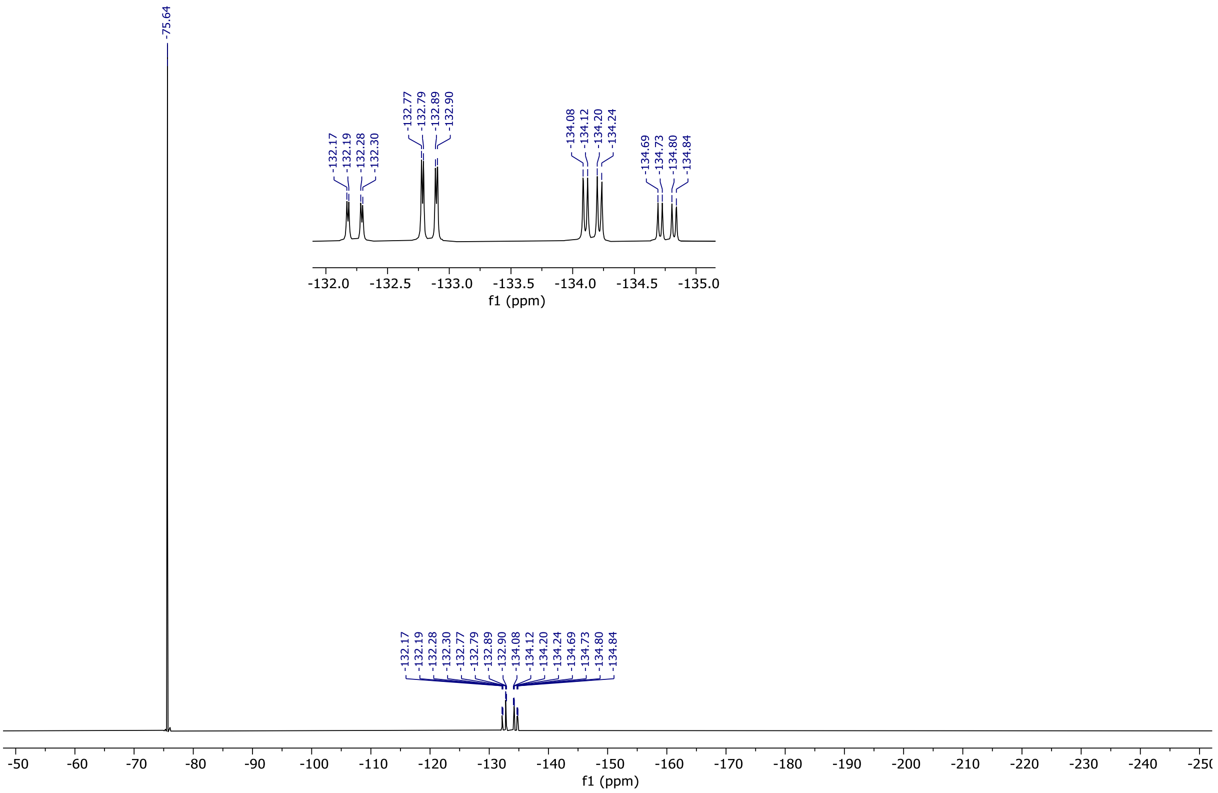
-75.64
-132.17
-132.19
-132.28
-132.30
-132.77
-132.79
-132.89
-132.90
-134.08
-134.12
-134.20
-134.24
-134.69
-134.73
-134.80
-134.84
f1 (ppm)

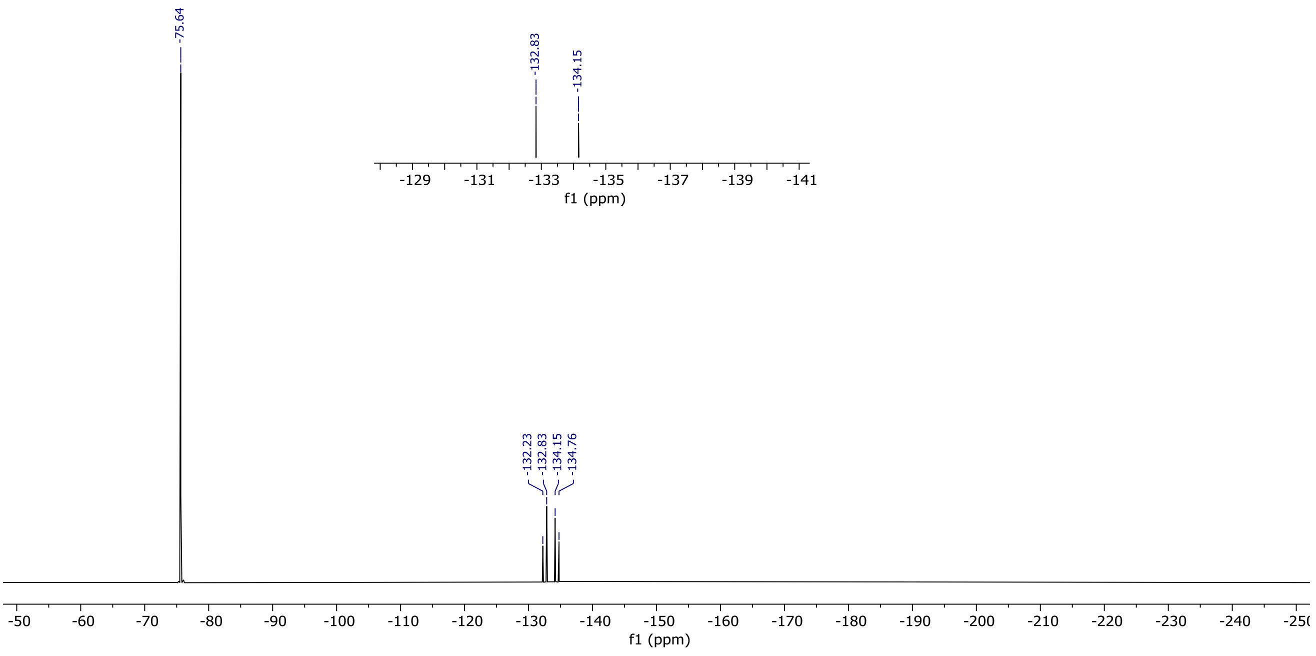
-75.64
-132.83
-134.15
-132.23
-134.76
f1 (ppm)

Experimental procedures 2. Polymer and Nanoparticle Synthesis and Testing

Polymerization of hydroxyethyl acrylamide using PFP-DMP

N-Hydroxyethyl acrylamide (HEA) (0.5 g, 4.34 mmol), Pentafluorophenyl 2-(Dodecylthiocarbonothioylthio)-2-methylpropionic acid (PFP-DMP) (0.092 g, 0.17 mmol), 4,4′-Azobis(4-cyanovaleric acid) (ACVA) (0.0097 g, 0.034 mmol were dissolved in 50:50 Toluene:Methanol (4 mL). Mesitylene (150 µL) was added as an internal reference. An aliquot was taken for NMR analysis in $CDCl_3$. The solution was degassed under N_2 for 30 mins. The reaction was stirred at 70 °C for 90 mins. An aliquot was taken for NMR analysis in MeOD. The reaction was rapidly cooled in liquid nitrogen and precipitated into diethyl ether. The polymer was reprecipitated into diethyl ether from methanol twice to yield a yellow polymer product that was dried under vacuum. 96 % conversion by NMR, M_n (Theoretical) = 3400 g.mol^{-1} M_n (SEC) = 5800 g.mol^{-1} M_n/M_w (SEC) = 1.16.

Gold nanoparticle synthesis

55 nm gold nanoparticles were synthesized by a modified step growth method developed by Bastús et al.[18] A solution of 2.2 mM sodium citrate in Milli-Q water (150 mL) was heated under reflux for 15 min under vigorous stirring. After boiling had commenced, 1 mL of $HAuCl_4$ (25 mM) was injected. The color of the solution changed from yellow to bluish gray and then to soft pink in 10 min, 1 mL was taken for DLS and UV/Vis analysis. Immediately after the synthesis of the Au seeds and in the same reaction vessel, the reaction was cooled until the temperature of the solution reached 90 °C. Then, 1 mL of a $HAuCl_4$ solution (25 mM) was injected. After 20 min, the reaction was finished. This process was repeated twice. After that, the sample was diluted by adding 85 mL of MilliQ water and 3.1 mL of 60 mM sodium citrate. This solution was then used as a seed solution, and three further portions of 1.6 mL of 25 mM $HAuCl_4$ were added with 20 min between each addition. Following completion of this step 1 mL was taken for DLS and UV/Vis analysis. The sample was diluted by adding 135 mL of MilliQ water and 4.9 mL of 60 mM sodium citrate. This solution was then used as a seed solution, and the process was repeated with three further additions of 2.5 mL of 25 mM $HAuCl_4$, this solution was analyzed by DLS and UV/Vis and target size of 35 nm was reached and the solution was cooled. After that, the sample was diluted by adding 215 mL of MilliQ water and 7.8 mL of 60 mM sodium citrate. This solution was then used as a seed solution, and three further portions of 3.9 mL of 25 mM $HAuCl_4$ were added with 20 min between each addition. Following completion of this step aliquots were taken for DLS and UV/Vis analysis. This solution was stored in the dark and used without further purification.

Functionalization of PHEA with DBCO

PFP-PHEA (500 mg, 0.15 mmol), dibenzocylclooctyne-amine (81 mg, 0.29 mmol) were dissolved in 2 mL DMF. The reaction was stirred at room temperature for 16 h. The polymer was precipitated into diethyl ether from methanol three times and dried under vacuum. The resulting polymer was an off white solid. IR indicated loss of C=O stretch corresponding to the PFP ester.

Capture of glycans onto DBCO-PHEA

In a typical reaction, DBCO-PHEA (1 mg, 0.32 μmol) and azidopropyl-linked glycan (2 eq) was dissolved in 1 mL milliQ water and left to react overnight on a tube roller. The solution was used immediately for immobilization onto AuNPs.

Gold nanoparticle functionalization using glyco-PHEA polymers

100 μL of 1 mg.mL^{-1} of polymer solution was added to 1 mL of OD 1 particles and left for 30 minutes at room temperature on a tube roller. After 30 mins, particles were centrifuged at 7000 rpm the supernatant was removed and resuspended in 1 mL milliQ H_2O. This was repeated a further two times to ensure complete removal of any unattached polymer. Stability was confirmed by incubating in 10 mM HEPES buffer for 30 mins.

Lectin-induced aggregation studies by Absorbance

A stock solution of the lectin was made up (1 mg.mL^{-1} for SBA and 0.01 mg.mL^{-1} for Galectin-3) in 10 mM HEPES buffer with 0.15 M NaCl, 0.1 mM CaCl2 and 0.01 mM MnCl2. 25 μL serial dilution was made up in the same buffer in a clear, flat bottom, half-area 96-well microtitre plate. 25 μL of the glycoAuNP were added to each well and incubated at room temperature for 30 mins. After 30 minutes, an absorbance spectrum was recorded from 450 nm -700 nm with 10 nm intervals.

Biolayer interferometry

Biolayer Interferometry was carried out on ForteBio Octet Red96 (Forte Bio, USA). Assays were performed in black 96 well half area plates. Assays were carried out at 30 °C and agitated at 1,000 rpm. Streptavidin (SA) biosensor tips (Forte Bio, USA) were hydrated in milliQ H_2O water for at least 10 mins prior to use. A stable baseline was established in milliQ water for 1 minute. The biosensors were functionalized by loading with 10 μg/mL biotinylated Galectin-3 in PBS for 5 mins followed by a 1 minute equilibration step in 10 mM HEPES with 0.15 M NaCl, 0.1 mM $CaCl_2$ and $MnCl_2$ to remove and unbound protein and to establish a stable baseline. Following protein immobilization, the binding association with glycoAuNPs was carried out in 10 mM HEPES with 0.15 M NaCl, 0.1 mM $CaCl_2$ and $MnCl_2$, for 30 minutes followed by dissociation in 10 mM HEPES with 0.15 M NaCl, 0.1 mM $CaCl_2$ and $MnCl_2$ for 10 minutes.

Lectin-induced aggregation studies by Dynamic Light Scattering (DLS)

25 μL of lectin was added to 25 μL of the glycoAuNPs in a disposable, ultralow volume plastic cuvette to give the indicated concentrations. Average diameter measurements were taken every minute seconds for 1 hour at 37 °C.

Nanoparticle Characterization

Table S1: Characterization of unfunctionalized and functionalized AuNPs used in this study.

Code	Glycan	UVmax[(a)] (nm)	A_{SPR}/A_{450}[(b)]	Dh[(c)] (nm)	$D_{h\ (DLS)}$[(d)] (nm)
$AuNP_{55}$	-	533	2.06	55	56.7 ± 1.4
**15**-$PHEA_{25}$@$AuNP_{55}$	Gal β1,3 GlcNAc	540	2.06	68	63.8 ± 0.9
**16**-$PHEA_{25}$@$AuNP_{55}$	3FGal β1,3 GlcNAc	538	2.09	64	64.2 ± 1.3
**17**-$PHEA_{25}$@$AuNP_{55}$	6FGal β1,3 GlcNAc	540	2.01	68	60.0 ± 1.5
**18**-$PHEA_{25}$@$AuNP_{55}$	Gal β1,3 6FGlcNTFAc	539	2.06	66	62.1 ± 1.1
**19**-$PHEA_{25}$@$AuNP_{55}$	6FGal β1,3 6FGlcNTFAc	540	2.09	68	62.4 ± 1.7
**20**-$PHEA_{25}$@$AuNP_{55}$	Gal β1,3 6FGlcNAc	539	2.08	66	62.0 ± 1.3
**21**-$PHEA_{25}$@$AuNP_{55}$	6FGal β1,3 6FGlcNAc	538	2.08	64	60.7 ± 0.8
**22**-$PHEA_{25}$@$AuNP_{55}$	Gal β1,3 6,6diFGlcNAc	540	2.09	68	62.4 ± 1.5
**23**-$PHEA_{25}$@$AuNP_{55}$	Gal β1,3 6,6diFGlcNTFAc	540	2.06	68	63.4 ± 1.6

(a) SPR absorption maximum; (b) Absorbance ratio of SPR to 450 nm; (c) Estimated from UV-Vis[6]; (d) From dynamic light scattering.

XPS

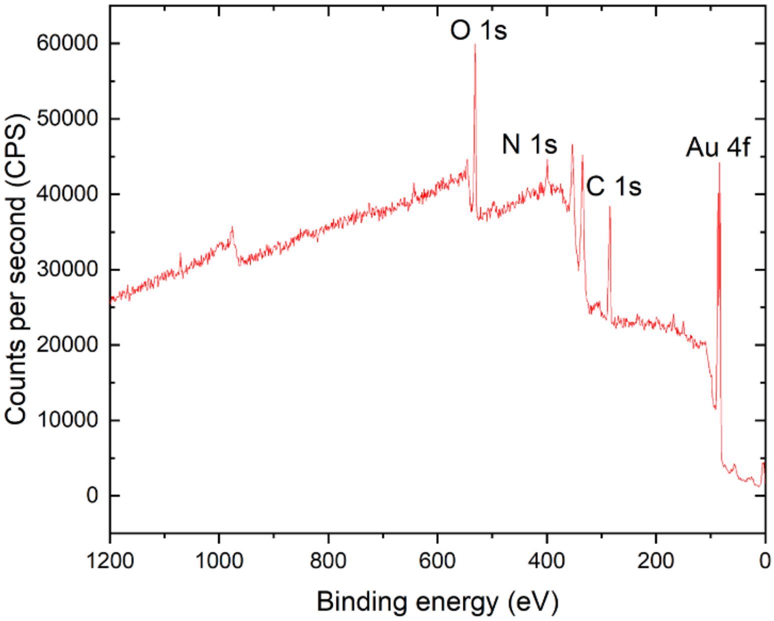

Figure S1. Representative XPS survey scan of glycopolymer functionalised AuNP Gal β1,3 6,6diF GlcNAc (**22**)-PHEA25@AuNP55)

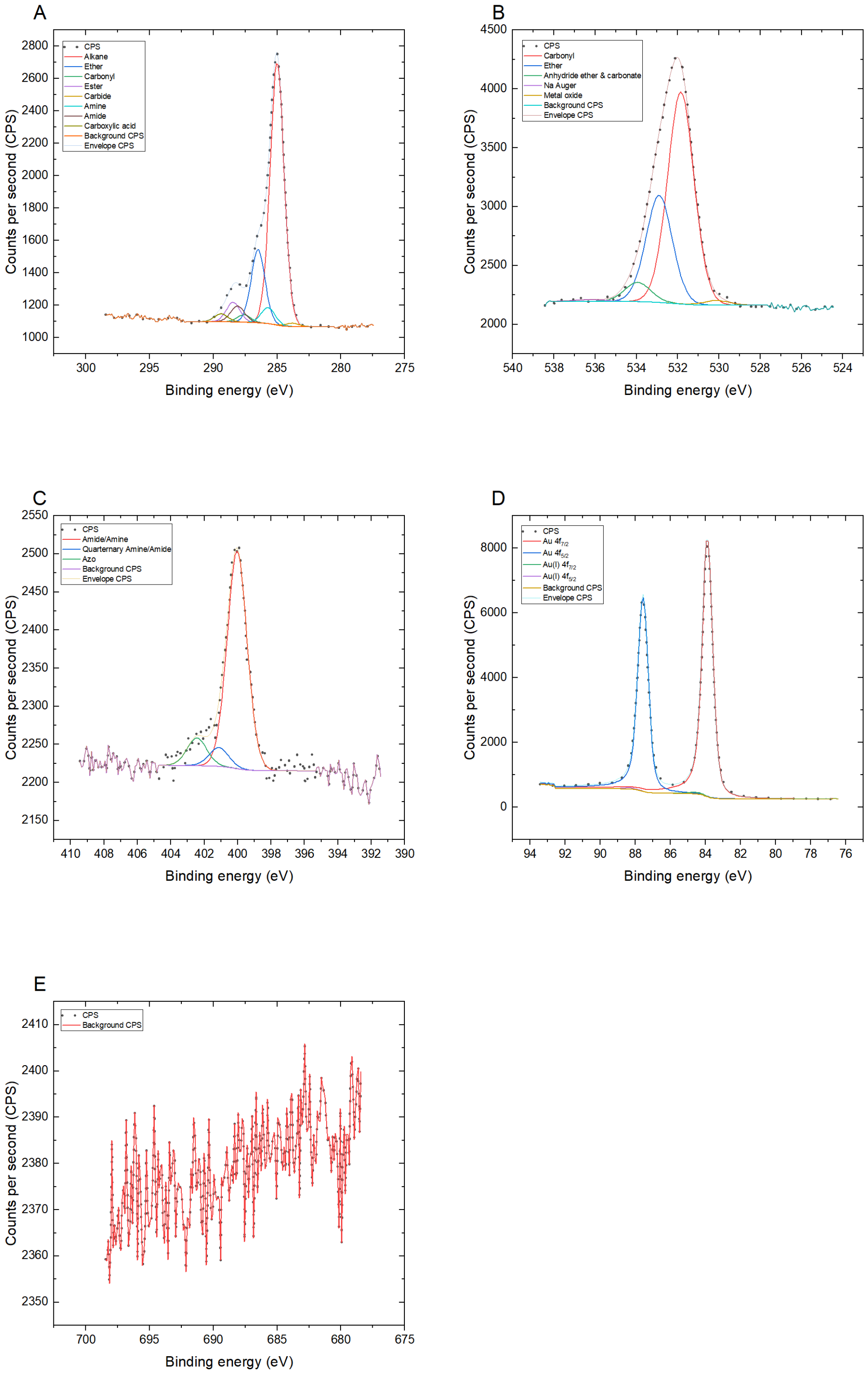

Figure S2. XPS of Gal β1,3GlcNAc **(15)** **-**PHEA$_{25}$@AuNP$_{55}$ A) C 1*s* B) O 1*s* C) N 1*s* D) Au 4*f* and F 1*s*

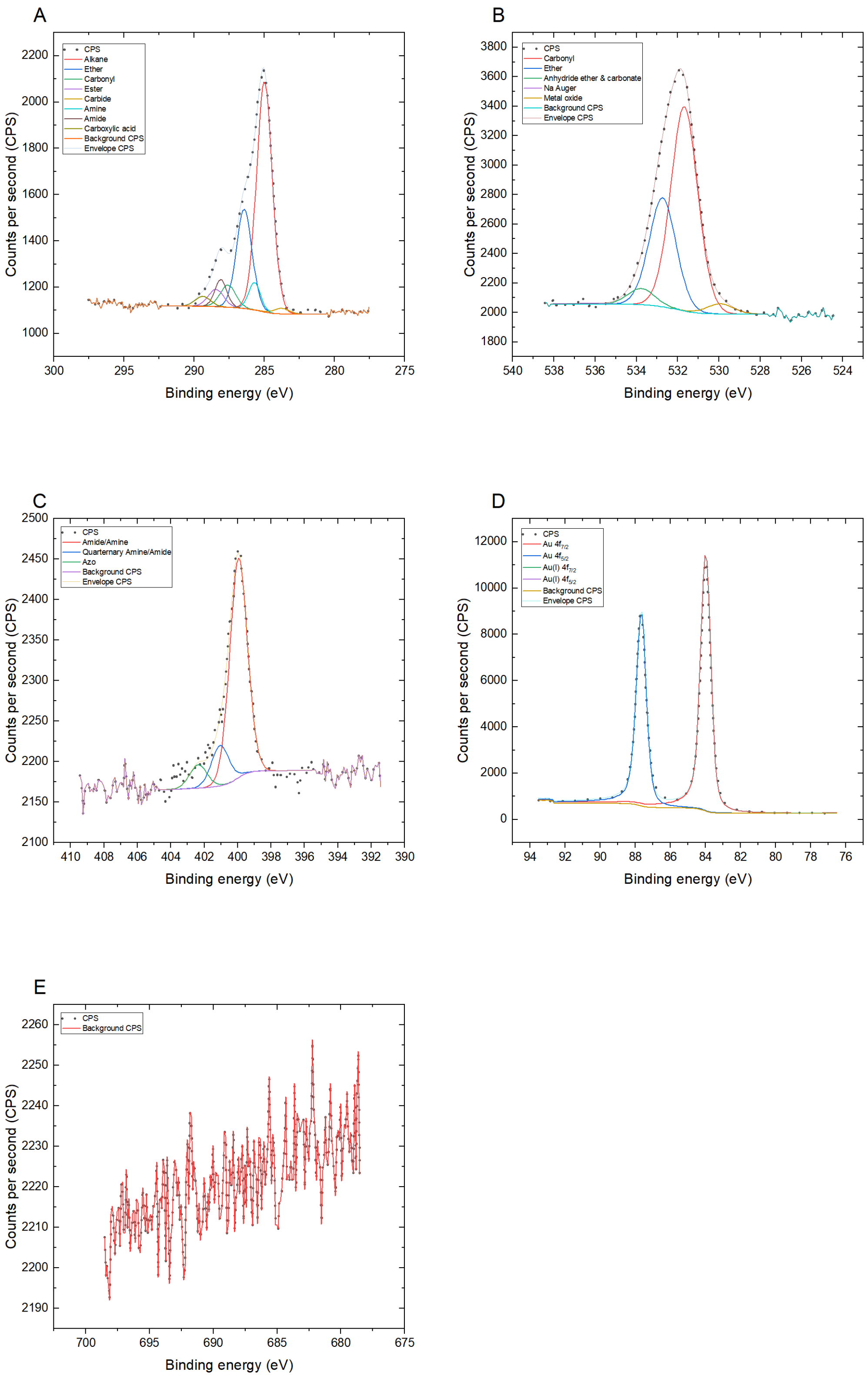

Figure S3. XPS of 3FGal β1,3 GlcNAc (**16**)-PHEA$_{25}$@AuNP$_{55}$ A) C 1*s* B) O 1*s* C) N 1*s* D) Au 4*f* and F 1*s*

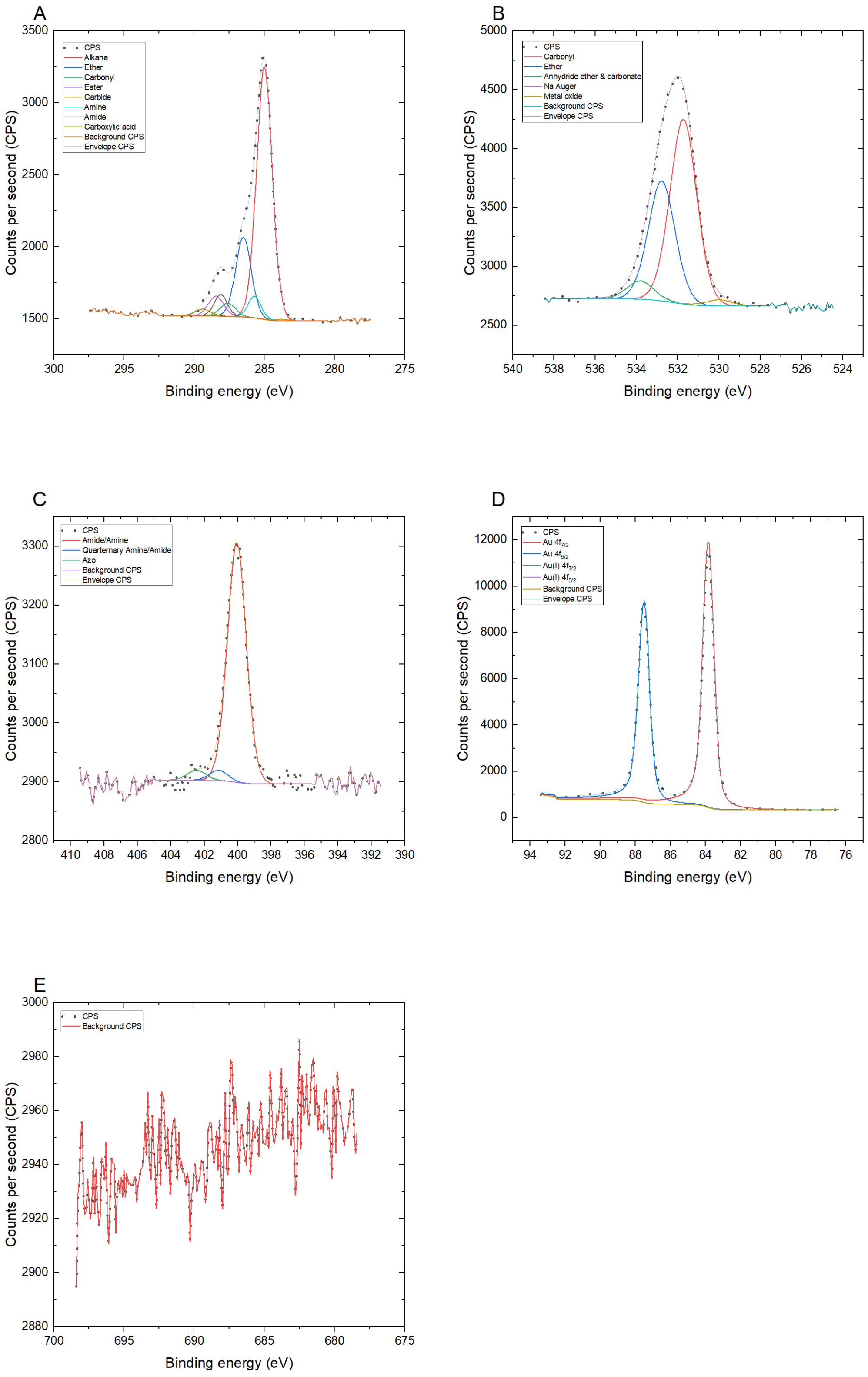

Figure S4. XPS of 6FGal β1,3 GlcNAc (**17**)-PHEA$_{25}$@AuNP$_{55}$ A) C 1*s* B) O 1*s* C) N 1*s* D) Au 4*f* and F 1*s*

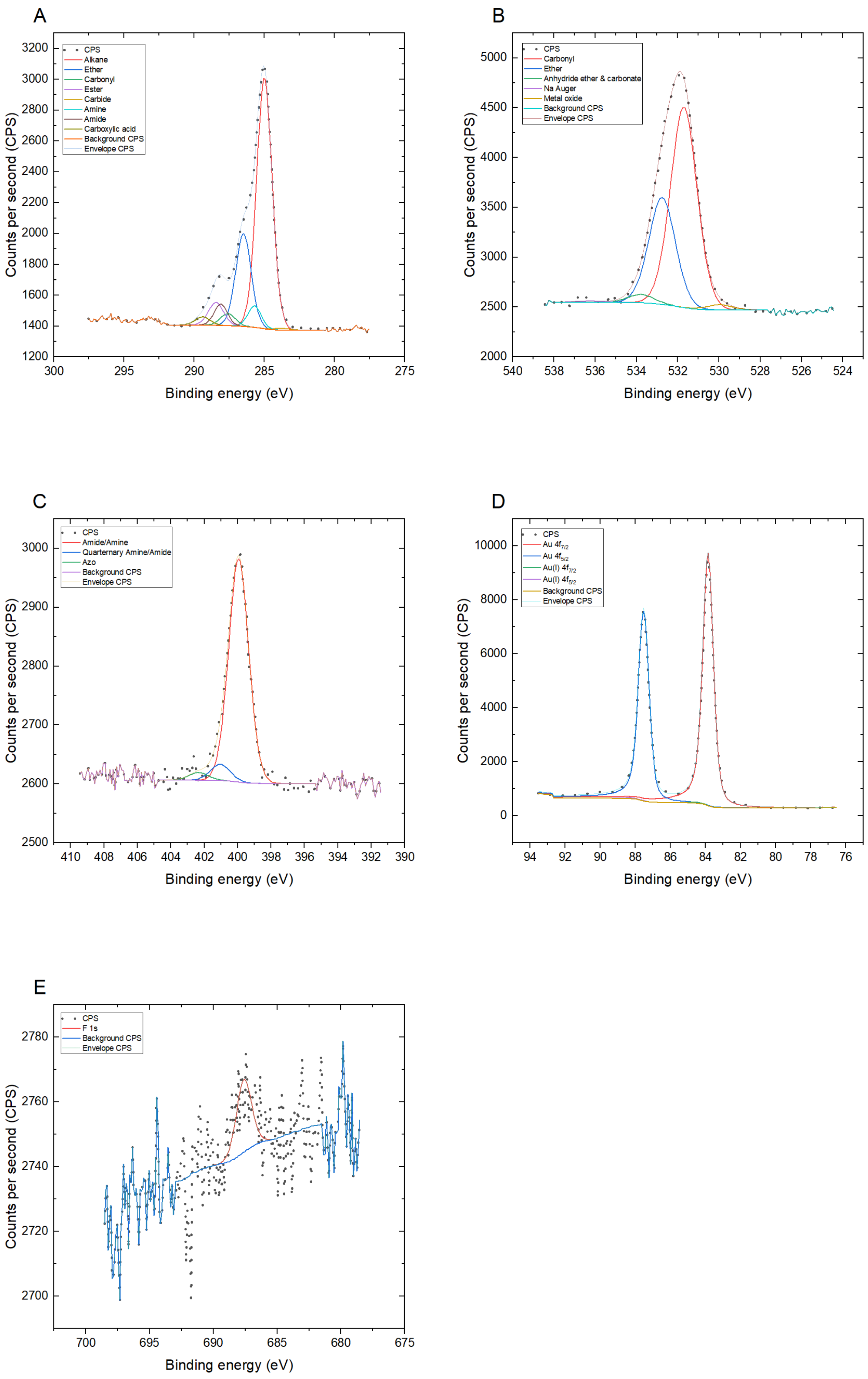

Figure S5. XPS of Gal β1,3 6,6diFGlcNAc (**22**)-PHEA$_{25}$@AuNP$_{55}$ A) C 1*s* B) O 1*s* C) N 1*s* D) Au 4*f* and F 1*s*

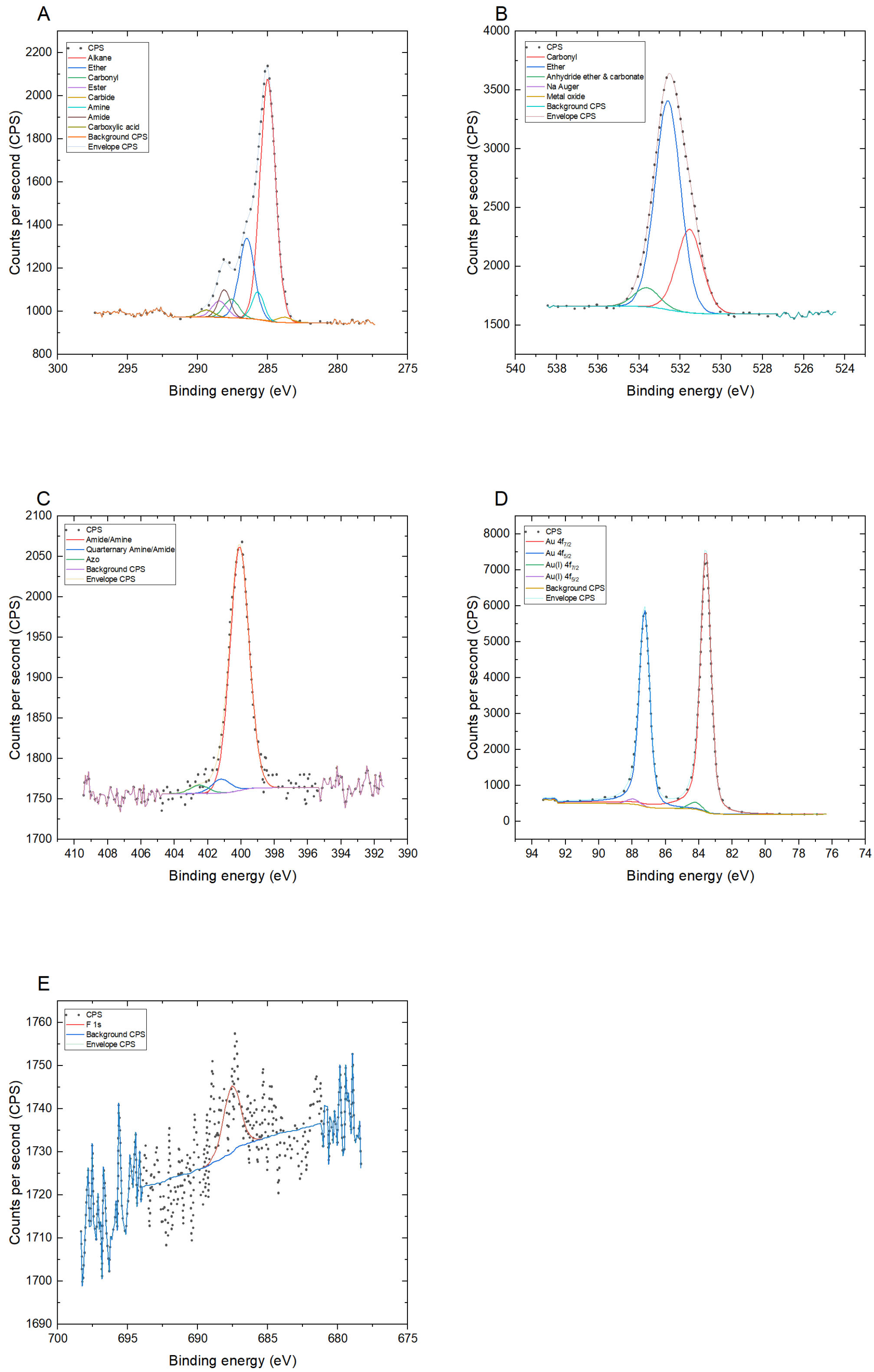

Figure S6. XPS of Gal β1,3 6,6diFGlcNTFAc (**23**)-$PHEA_{25}$@$AuNP_{55}$ A) C 1*s* B) O 1*s* C) N 1*s* D) Au 4*f* and F 1*s*

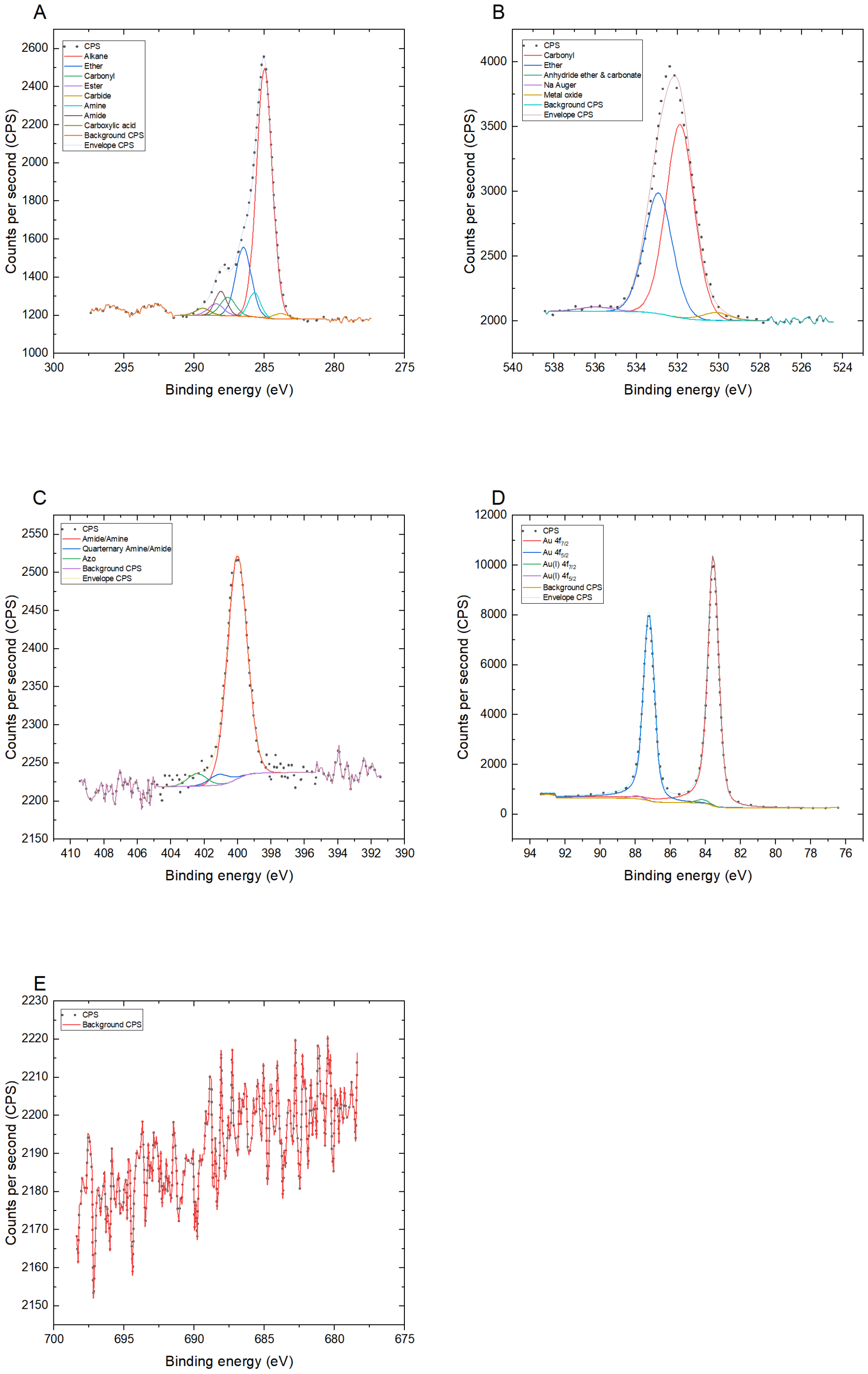

Figure S7. XPS of Gal β1,3 6FGlcNAc (**20**)-PHEA$_{25}$@AuNP$_{55}$ A) C 1*s* B) O 1*s* C) N 1*s* D) Au 4*f* and F 1*s*

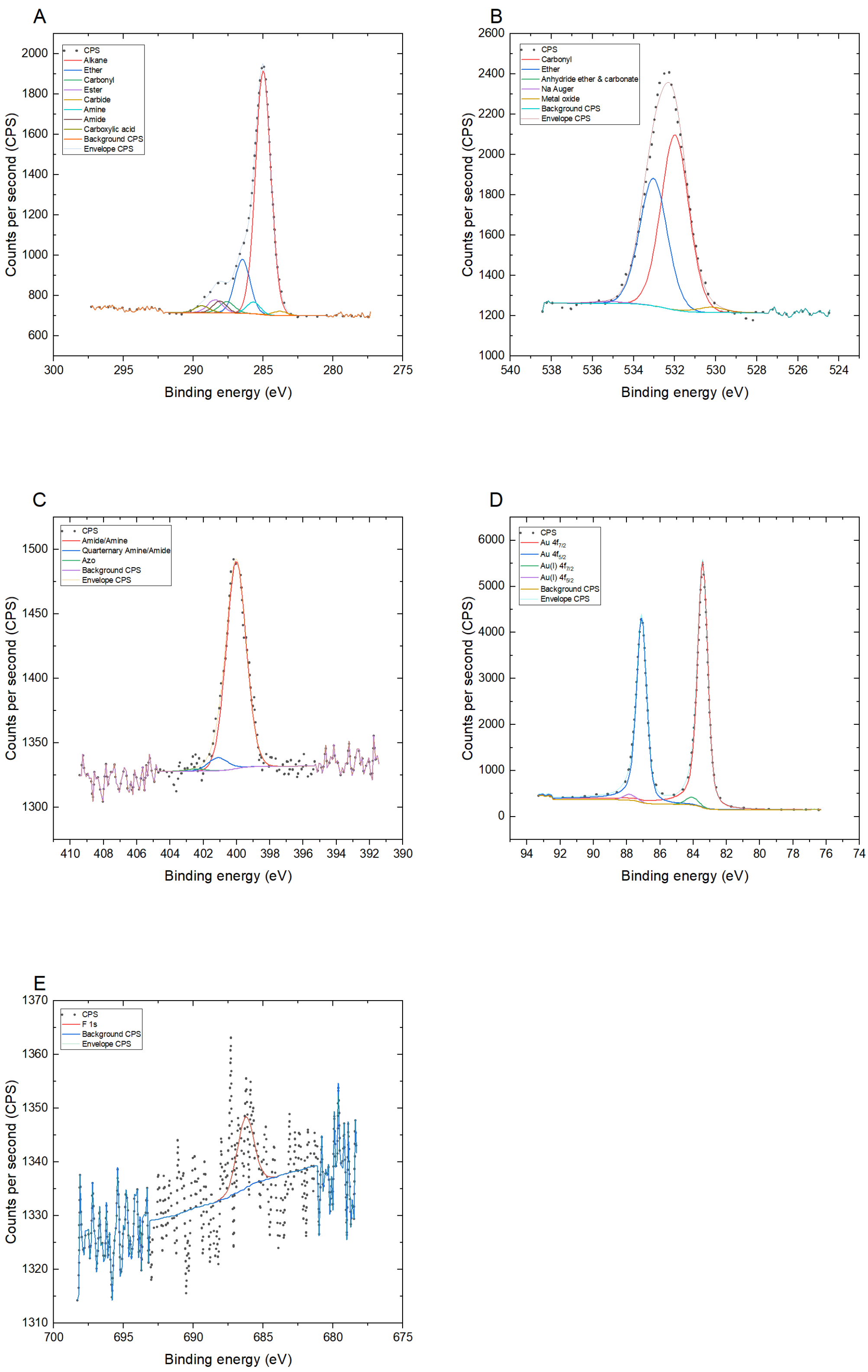

Figure S8. XPS of 6FGal β1,3 6FGlcNAc (**21**)-PHEA$_{25}$@AuNP$_{55}$ A) C 1*s* B) O 1*s* C) N 1*s* D) Au 4*f* and F 1*s*

Table S2. Elemental composition of nanoparticles determined by XPS

Particle Composition		Elemental Percentage Compositions (%)				Elemental ratios	
Code	Glycan	C 1*s*	O 1*s*	N 1*s*	Au 4*f*	N 1*s*/C 1*s*	N 1*s*/Au 4*f*
**15**-$PHEA_{25}$@$AuNP_{55}$	Gal β1,3 GlcNAc	16.85	26.04	1.542	55.57	0.092	0.027
**16**-$PHEA_{25}$@$AuNP_{55}$	3FGal β1,3 GlcNAc	11.69	19.25	1.237	67.82	0.106	0.018
**17**-$PHEA_{25}$@$AuNP_{55}$	6FGal β1,3 GlcNAc	15.08	19.51	1.409	64	0.093	0.022
**22**-$PHEA_{25}$@$AuNP_{55}$	Gal β1,3 6,6diFGlcNAc	16.31	25.04	1.574	57.08	0.097	0.028
**23**-$PHEA_{25}$@$AuNP_{55}$	Gal β1,3 6,6diFGlcNTFAc	14.3	25.21	1.57	58.91	0.110	0.027
**20**-$PHEA_{25}$@$AuNP_{55}$	Gal β1,3 6FGlcNAc	12.83	21.44	1.262	64.47	0.098	0.020
**21**-$PHEA_{25}$@$AuNP_{55}$	6F-Gal β1,3 6FGlcNAc	18.16	21.82	1.148	58.85	0.063	0.020

F 1*s* was not included in the elemental percentage compositions due to the very low percentages making quantitative analysis difficult

Aggregation assays

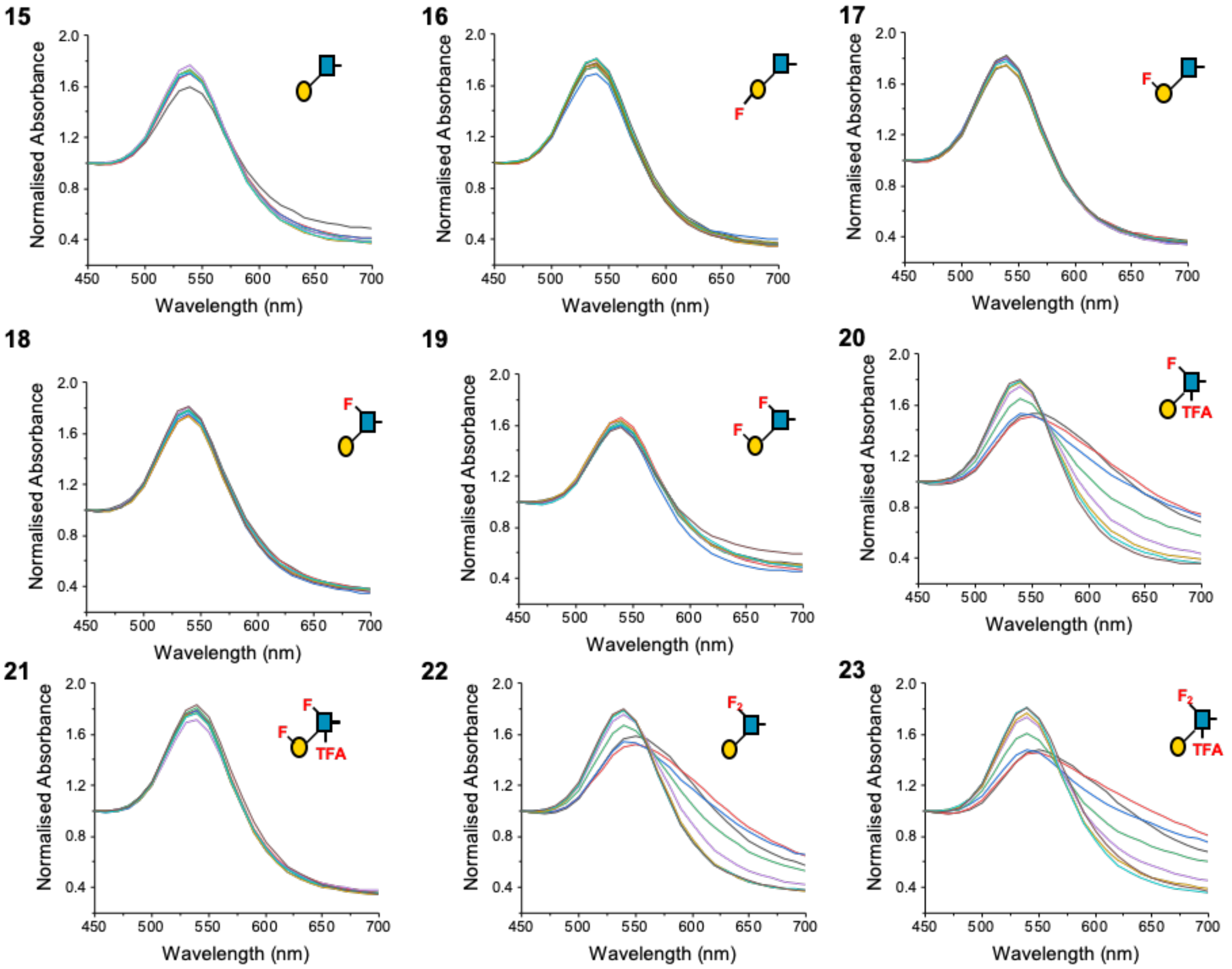

Figure S9: UV-Vis spectra of particles (Glycan-PHEA$_{25}$@AuNP$_{55}$) in response to Galectin-3 (starting at 1 mg.mL^{-1}). Labelled with their molecule number from the main text with their scientific notation for glycans) (SNFG) inlaid.

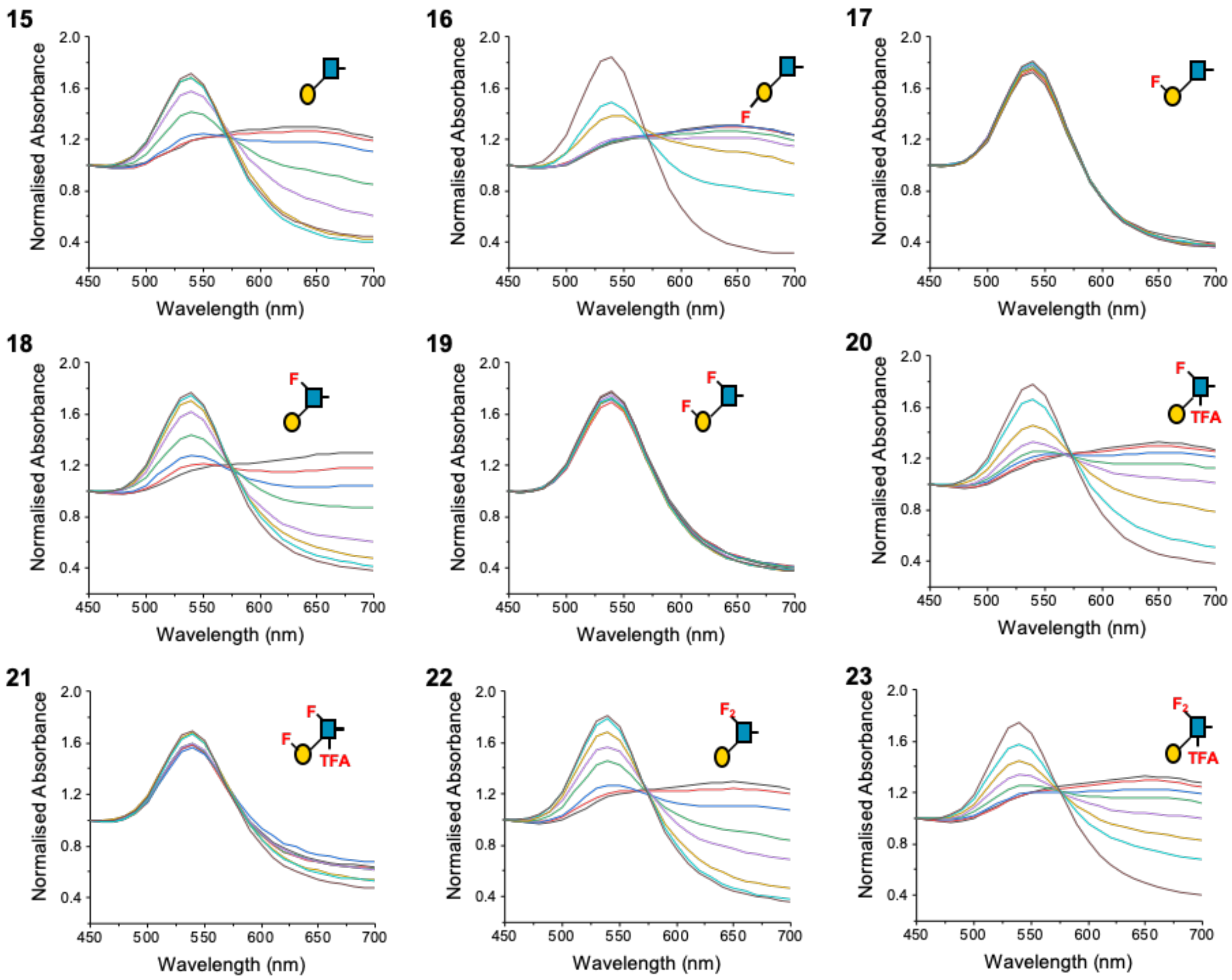

Figure S10: UV-Vis spectra of particles (Glycan-$PHEA_{25}$@$AuNP_{55}$) in response to Galectin-3 (starting at 0.01 $mg.mL^{-1}$). Labelled with their molecule number from the main text with their scientific notation for glycans) (SNFG) inlaid.

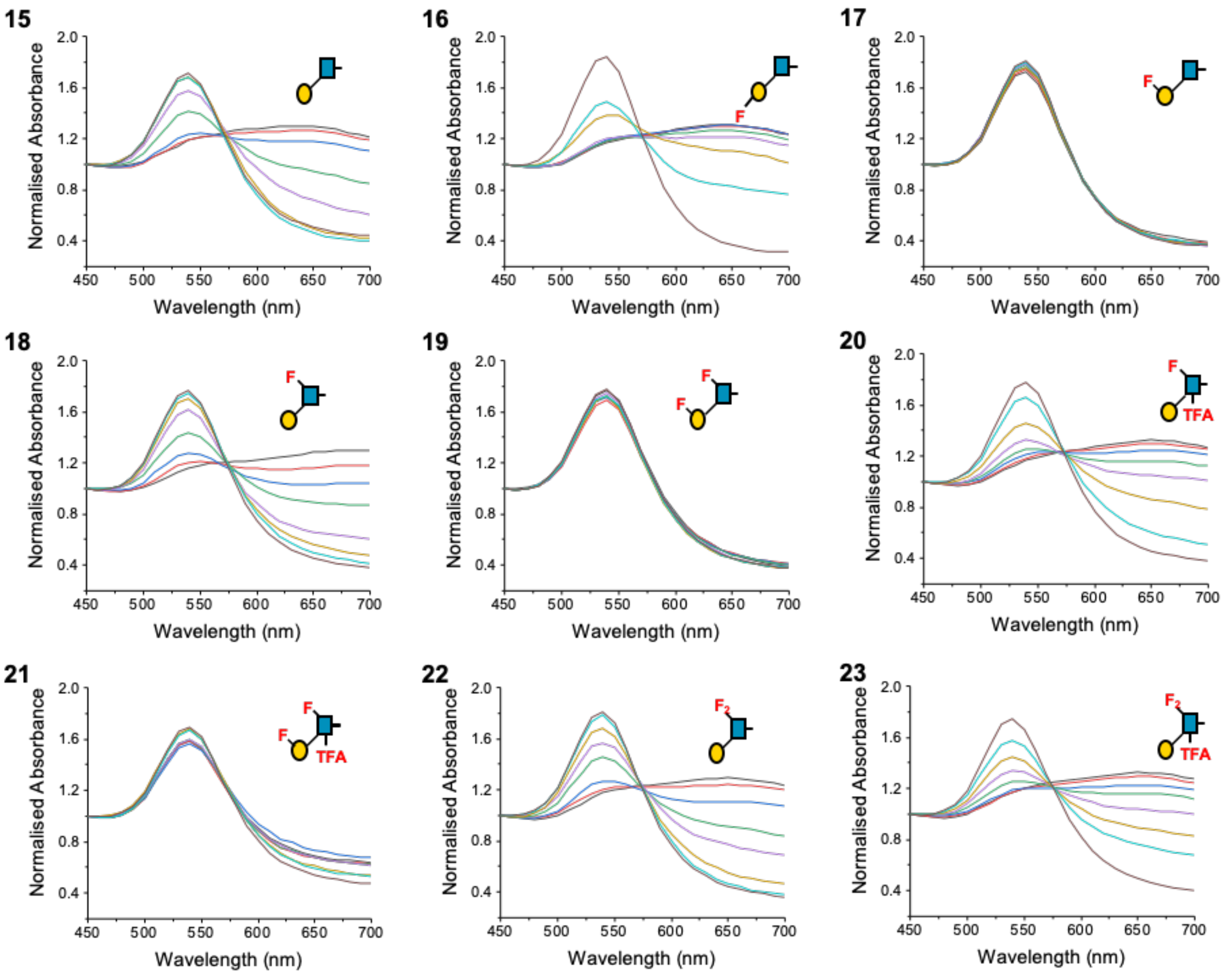

Figure S11: UV-Vis spectra of particles (Glycan-PHEA$_{25}$@AuNP$_{55}$) in response to Galectin-7 (starting at 0.01 mg.mL^{-1}). Labelled with their molecule number from the main text with their scientific notation for glycans) (SNFG) inlaid.

Table S3: Apparent dissociation constant (K_d apparent) in nM for each glycoAuNP and lectin combination determined by Abs_{700}. ND (not determined) means they showed no interaction in the concentration range used and have a value could not be extracted

Code	Glycan	Galectin-3 K_d (nM)	Galectin-7 K_d (nM)	SBA K_d (nM)
**15**-$PHEA_{25}$@$AuNP_{55}$	Gal β1,3 GlcNAc	15.9 ± 0.6	ND	ND
**16**-$PHEA_{25}$@$AuNP_{55}$	3FGal β1,3 GlcNAc	6.0 ± 0.7	45.1 ± 1.8	ND
**17**-$PHEA_{25}$@$AuNP_{55}$	6FGal β1,3 GlcNAc	ND	ND	ND
**18**-$PHEA_{25}$@$AuNP_{55}$	Gal β1,3 6FGlcNTFAc	9.8 ± 1.2	ND	884.6 ± 8.9
**19**-$PHEA_{25}$@$AuNP_{55}$	6FGal β1,3 6FGlcNTFAc	ND	61.6 ± 1.9	ND
**20**-$PHEA_{25}$@$AuNP_{55}$	Gal β1,3 6FGlcNAc	30.0 ± 4.1	ND	ND
**21**-$PHEA_{25}$@$AuNP_{55}$	6FGal β1,3 6FGlcNAc	ND	89.4 ± 3.2	ND
**22**-$PHEA_{25}$@$AuNP_{55}$	Gal β1,3 6,6diFGlcNAc	9.4 ± 1.5	ND	886.7 ± 9.9
**23**-$PHEA_{25}$@$AuNP_{55}$	Gal β1,3 6,6diFGlcNTFAc	14.0 ± 0.8	62.3 ± 2.9	844.5 ± 8.4

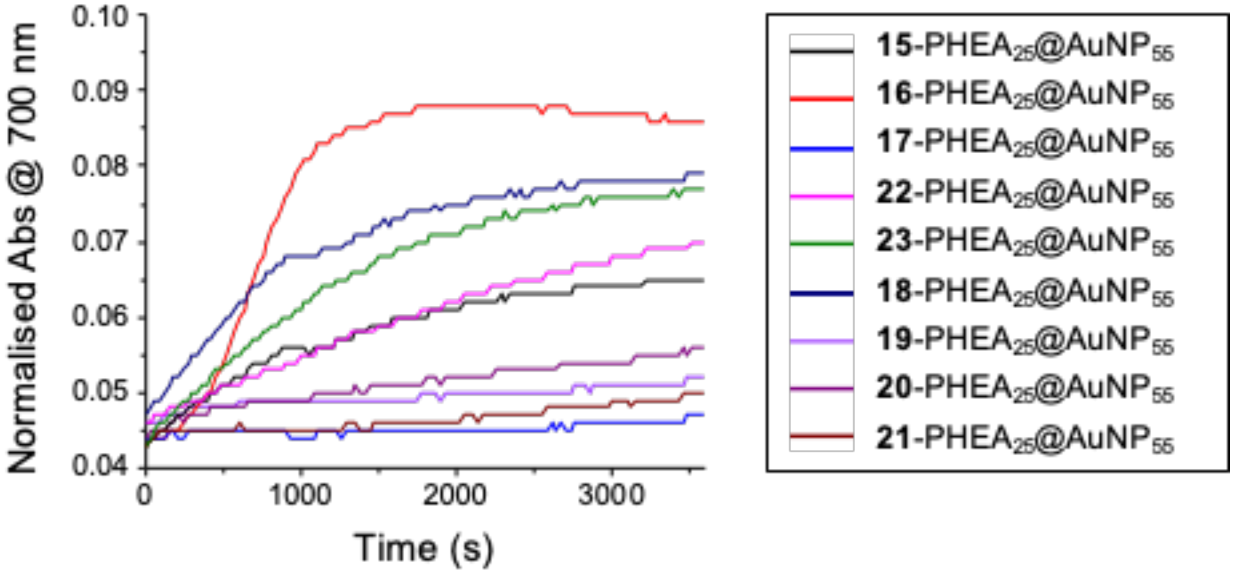

Figure S12: Kinetic of aggregation: increase in Abs_{700} by UV-Vis over time due to aggregation induced by addition of 1 ug.mL^{-1} Galectin-3.

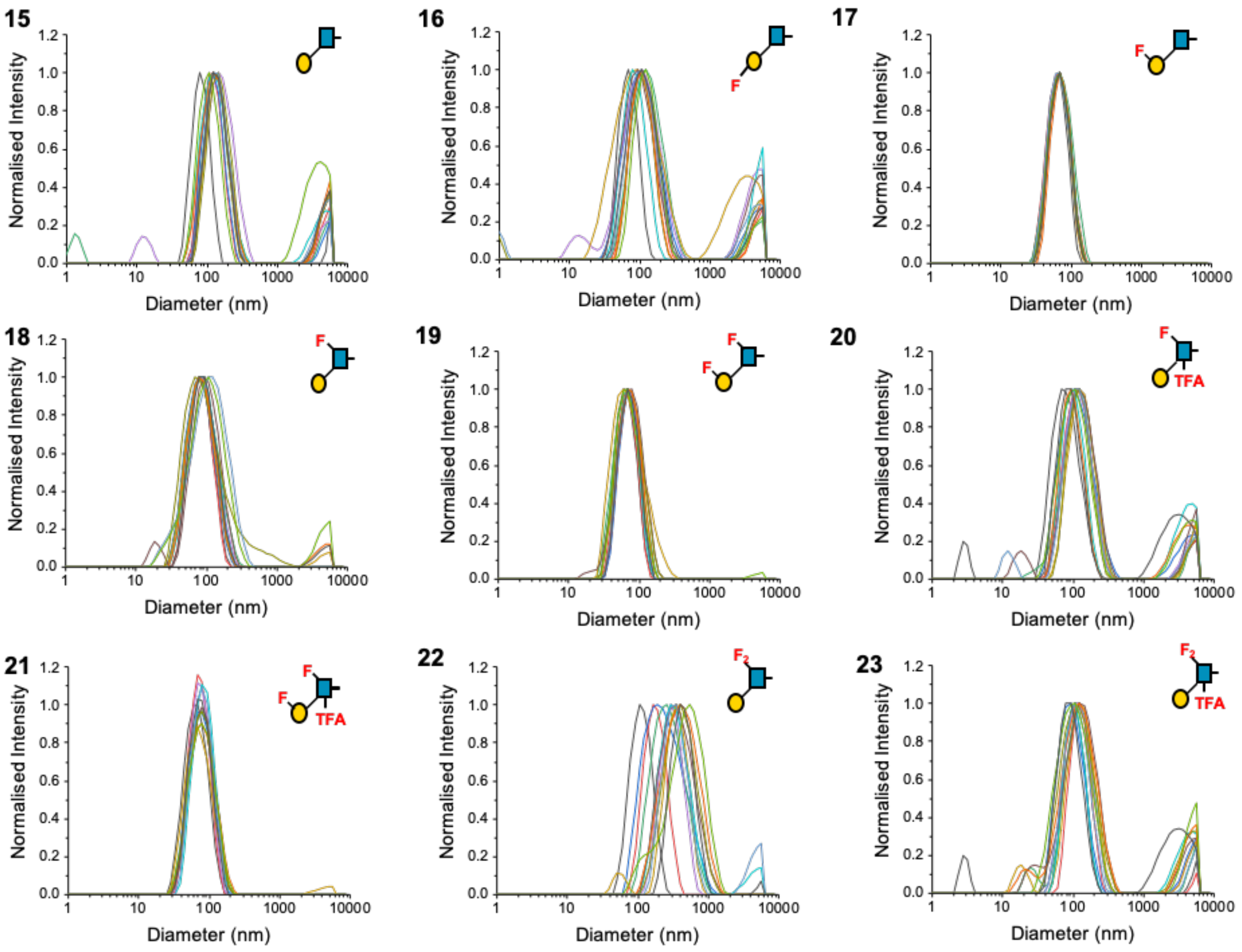

Figure SX: Kinetic of aggregation: increase in particle size distribution by DLS over time due to aggregation induced by addition of 1 ug.mL^{-1} Galectin-3.

References:

1. Keenan, T. *et al*. The characterisation of a galactokinase from Streptomyces coelicolor. *Carbohydrate research* **472**, 132-137 (2019).
2. Valverde, P. *et al*. Chemoenzymatic synthesis of 3-deoxy-3-fluoro-L-fucose and its enzymatic incorporation into glycoconjugates. *Chemical Communications* (2020).
3. Pangborn, A.B., Giardello, M.A., Grubbs, R.H., Rosen, R.K. & Timmers, F.J. Safe and convenient procedure for solvent purification. *Organometallics* **15**, 1518-1520 (1996).
4. Huang, Y., Shaw, M.A., Mullins, E.S., Kirley, T.L. & Ayres, N. Synthesis and anticoagulant activity of polyureas containing sulfated carbohydrates. *Biomacromolecules* **15**, 4455-4466 (2014).
5. Fukase, K. *et al*. Synthesis and biological activities of lipid A analogs possessing β-glycosidic linkage at 1-position. *Bulletin of the Chemical Society of Japan* **76**, 485-500 (2003).

6. Mandal, S., Sharma, N. & Mukhopadhyay, B. H2SO4-silica promoted direct formation of β-glycosides of N-acetyl glycosylamines under microwave conditions. *Synlett* **2009**, 3111-3114 (2009).
7. Huang, K. *et al*. Enzymatic synthesis of N-acetyllactosamine from lactose enabled by recombinant β1, 4-galactosyltransferases. *Organic & biomolecular chemistry* (2019).
8. Choudhury, I., Minoura, N. & Uzawa, H. Chemoenzymatic synthesis of the sialyl-α-(2→ 3′)-lactosamine trisaccharide with a 3-aminopropyl group as a spacer at the reducing end. *Carbohydrate research* **338**, 1265-1270 (2003).
9. Yu, H. *et al*. Highly efficient chemoenzymatic synthesis of β1–3-linked galactosides. *Chemical Communications* **46**, 7507-7509 (2010).
